# Recurrence prediction using circulating tumor DNA in patients with early-stage non-small cell lung cancer after treatment with curative intent: A retrospective validation study

**DOI:** 10.1371/journal.pmed.1004574

**Published:** 2025-04-15

**Authors:** Milou M. F. Schuurbiers, Christopher G. Smith, Koen J. Hartemink, Robert C. Rintoul, Davina Gale, Kim Monkhorst, Bas L. R. Mandos, Anna L. Paterson, Daan van den Broek, Nitzan Rosenfeld, Michel M. van den Heuvel, On Behalf of the LEMA Study Group and the LUCID Study Group

**Affiliations:** 1 Department of Pulmonary Diseases, Radboud University Medical Center, Nijmegen, Netherlands; 2 NeoGenomics, Inc., Fort Myers, Florida, United States of America; 3 Department of Surgery, The Netherlands Cancer Institute, Amsterdam, Netherlands; 4 Department of Oncology, University of Cambridge, Cambridge, United Kingdom; 5 Royal Papworth Hospital NHS Foundation Trust, Cambridge, United Kingdom; 6 Cancer Research UK Cambridge Centre, Cambridge, United Kingdom; 7 Cancer Research UK Cambridge Institute, University of Cambridge, Cambridge, United Kingdom; 8 Department of Pathology, The Netherlands Cancer Institute, Amsterdam, Netherlands; 9 Department of Histopathology, Cambridge University Hospitals, Cambridge, United Kingdom; 10 Department of Laboratory Medicine, The Netherlands Cancer Institute, Amsterdam, Netherlands; 11 Barts Cancer Institute, Queen Mary University of London, London, United Kingdom; Washington University in St Louis, UNITED STATES OF AMERICA

## Abstract

**Background:**

Despite treatment with curative intent, many patients with localized non-small cell lung cancer (NSCLC) develop recurrence. The current challenge is to identify high-risk patients to guide adjuvant treatment. Identification of residual disease by detection of circulating tumor DNA (ctDNA) may allow more accurate clinical decision-making, but its reliability in NSCLC is not established. We aimed to build on previous data to validate a tissue-informed personalized ctDNA assay, to predict recurrence in patients with early-stage disease.

**Methods and findings:**

Tumor tissue and plasma was collected from patients with stage 0–III NSCLC enrolled to LEMA (**L**ung cancer **E**arly **M**olecular **A**ssessment trial, NCT02894853). Serial plasma was collected before and after definitive treatment, with the latter including key timeframes of interest (1–3 days post-treatment, between 14 and 122 days after treatment end, and ≥14 days after treatment end). Somatic mutations identified by tumor exome sequencing were used to design patient-specific assays, to analyze ctDNA. Results were compared and combined with an independent dataset (LUCID; **LU**ng **C**ancer C**I**rculating Tumour **D**na study, NCT04153526). In LEMA, 130 patients (57% male; median age 66 years (range 44–82); 69% adenocarcinoma, 22% squamous cell carcinoma (SCC); 3%/49%/19%/29% with stage 0/I/II/III) were treated with curative intent. Tumor tissue originated from surgical resection or diagnostic biopsy in 118 and 12 patients respectively. LUCID included 88 patients (51% male; median age 72 years (range 44–88); 63% adenocarcinoma, 31% SCC; 49%/28%/23% with stage I/II/III). Before treatment, ctDNA was detected in 48% LEMA and 51% LUCID patients. Sensitivity, specificity, positive and negative predictive value of ctDNA detection post-treatment (≥1 positive sample ≥14 days after treatment end) to predict recurrence were 61%, 97%, 92% and 84% for LEMA and 64%, 96%, 90% and 83% for LUCID. In the combined cohort, ctDNA detection after treatment was associated with shorter recurrence-free survival (hazard ratio (HR) 11.4 (95% confidence interval (CI) [7.0,18.7]; *p* < 0.001)) and overall survival (HR 8.1 (95% CI [4.6,14.2]; *p* < 0.001)), accounting for guarantee-time bias. Of note, a key limitation of this work was the irregular sample collection schedules, during routine follow-up visits, of both studies.

**Conclusions:**

ctDNA detection predicted recurrence in independent retrospective cohorts with notable reproducibility, including near-identical detection rates and predictive values, confirming its ability to differentiate patients at high- versus low risk of recurrence. Our results support the potential of tissue-informed ctDNA analysis as a decision-support tool for adjuvant therapy in NSCLC.

## Author summary

**Why was this study done?:**

- Circulating tumor DNA (ctDNA) has emerged as a potentially powerful tool in the treatment of patients with cancer.
- After curative treatment, there is a risk that patients with early-stage lung cancer are treated sub-optimally; toxic therapy is sometimes administered to patients that don’t require additional treatment, while it is withheld from patients that would benefit from further intervention to prevent disease relapse.
- The authors have previously shown that ctDNA might be able to help healthcare providers decide which patients need additional therapy. Here they aimed to validate and build on this previous data by testing a new, independent group of patients.

**What did the researchers do and find?:**

- An independent cohort (the **L**ung cancer **E**arly **M**olecular Assessment, LEMA, study), comprising ~130 patients with early-stage lung cancer, was tested using the same ctDNA methodology, to determine the reproducibility of the original results. The results were notable in their similarity, showing some of the same associations with similar magnitude.
- The two datasets were combined to give the authors an even larger dataset to attempt to explore associations between ctDNA and clinical, biological and technical variables.
- Key findings include that; ctDNA is prognostic (i.e., detection is linked to patient survival) and predictive (i.e., detection indicates which patients’ disease are most likely to return and/or respond to treatment). However, ctDNA detection might be influenced by the biology of the patient’s tumor.

**What do these findings mean?:**

- These findings add to mounting data that ctDNA can positively contribute to patient care, by indicating the patients with worse outcomes, and guiding if and when treatment should be administered.
- Whilst encouraging, more work is needed before ctDNA can be routinely utilized in clinical practice. Fortunately, technological advancements continue to improve the detection and quantification of ctDNA, while large prospective clinical studies will provide stronger evidence for the absolute benefit it offers.
- A key limitation of this work was the irregular collection of blood samples from patients from both cohorts – ideally samples would be collected according to a regular and extended schedule, over an extended period, e.g., every 3 months over 5 years.

## Abstract 3

Milou MF Schuurbiers, Christopher G Smith and colleagues validate a tissue-based personalized ctDNA assay to predict recurrence in patients with non-small cell lung cancer in a new independent group of patients.

## Introduction

At diagnosis, 40%−50% of patients with non-small cell lung cancer (NSCLC) present with stage I-III disease [1,2]. Stage I–II disease is often treated with surgery or radiotherapy, while stage III patients may be offered surgery or chemoradiotherapy [3]. The 5-year overall survival (OS) rates for patients treated with curative intent remain low [4]. For patients with resected stage II–III NSCLC, adjuvant chemotherapy is recommended. In patients with stage I disease, the 5-year recurrence rate is up to 29%, indicating some may benefit from additional treatment. Adjuvant chemotherapy is often offered, with an efficacy of 4%–5% absolute survival benefit at 5-years [3,5–7]. Some adjuvant therapies are guided by a patient’s biomarker status (e.g., osimertinib for resected stage IB-III patients harboring EGFR activating mutations) [8,9]. Furthermore, approvals have increased access to immune checkpoint inhibitors in patients with stage IB–IIIA disease, with limited restriction [10–13]. As a result of these, and other expected perioperative or (neo-)adjuvant drugs for stage II–III disease, overtreatment may become increasingly common.

There is a clinical unmet need to identify patients at high risk of relapse. Detection of molecular residual disease (MRD) is a promising strategy to select patients for adjuvant treatment and to monitor disease course after treatment. Circulating tumor DNA (ctDNA) has been demonstrated to improve risk stratification in advanced- [14–16] and early-stage NSCLC [17,18]. Several studies have shown that patients with post-operatively detected ctDNA have a higher probability of recurrence (79%−100%) [19–26]. However, residual ctDNA levels in post-treatment plasma can be very low, resulting in a variable sensitivity of assays to predict recurrence, ranging from 30% to 85% [20,22–24,27,28]. As shown by Gale *and colleagues*, this may be improved through the use of tissue-informed personalized assays, based on somatic variants detected in matched tumor tissue, that can detect ctDNA to fractional concentrations as low as 0.0011% in patients who eventually relapsed [29]. Moreover, repeated surveillance testing for detection of MRD increased the chance of ctDNA detection in patients who developed recurrence while retaining a clinical specificity of >98.5%.

Despite this encouraging data, there remain questions about the reliability of ctDNA-based assays in NSCLC. The current study (**L**ung cancer **E**arly **M**olecular **A**ssessment trial, LEMA) aimed to validate a tissue-informed personalized ctDNA assay, which was previously assessed in the Lung cancer Circulating tumor DNA (LUCID) cohort [29], and predict recurrence in patients with early-stage NSCLC treated with curative intent. Furthermore, the LEMA cohort was combined with LUCID to assess the potential role for ctDNA analysis as a decision support tool for adjuvant therapy in NSCLC.

## Methods

### Patients

The multi‐centre Lung Early Molecular Assessment (LEMA) study, approved by the medical ethics committee of the Netherlands Cancer Institute (REC: NL54778.031.15, ClinicalTrials.gov; NCT02894853), enrolled treatment-naïve patients suspected of NSCLC who provided written informed consent. Details of the LEMA study and wider cohort can be found in the publication of Schouten and colleagues [30]. Briefly, this multi-center, prospective study aimed to explore the performance of protocolized molecular profiling of tissue and plasma, in the standard-of-care setting. Patients were included in this analysis based on (i) stage 0–III NSCLC diagnosis, (ii) availability of plasma samples and (iii) availability of tumor tissue specimens from surgical resection or diagnostic biopsies (S1 Fig). Results were compared against, and subsequently combined with results from LUCID (REC: 14/WM/1072, ClinicalTrials.gov Identifier: NCT04153526), which also included treatment naïve patients with stage IA-IIIB NSCLC treated with curative intent, analyzed using the same assay (both RaDaR version 1.0), as previously described [29]. Sample collection schedules for LEMA and LUCID are indicated in S2 Fig. Surveillance imaging was performed according to national guidelines (*i.e.,* a CT-scan every 6 months for the first 2 years after curative intent treatment, and after this yearly for a period of 3 years).

### Tissue and plasma analyses

DNA was extracted from formalin-fixed paraffin-embedded (FFPE) tissue sections and whole exome sequencing (WES) was performed. Plasma samples were collected before and after treatment. The RaDaR assay is based upon personalized multiplex PCR amplification of cell-free DNA (cfDNA). Tumor-specific variants, identified by WES of the primary tumor, were ranked and prioritized for inclusion in patient-specific custom panels targeting up to 48 amplicons (S2 Table). This variant-specific panel was combined with a fixed primer panel covering common population-specific single nucleotide polymorphisms, for internal sample quality control, and then applied to plasma and buffy coat DNA samples. Single nucleotide variants (SNVs) found by panel sequencing in buffy coat DNA were filtered to exclude germline mutations, mosaicism, or variants arising from clonal hematopoiesis of indeterminate potential. A proprietary statistical model was used to determine evidence of ctDNA presence (ctDNA positive) or absence (ctDNA negative), and an estimated variant allele fraction (eVAF) was calculated. Additional details are provided in the S1 Methods.

### Statistical analysis

To validate the tissue-informed ctDNA assay, detection results from LEMA were compared against the published results of LUCID [29]. Analyses were based on detection of ctDNA during four different pre-specified time windows: at baseline (pre-treatment), 1–3 days after treatment end, ≥14 days after treatment end (observation/follow-up), and during ‘landmark’, defined as the first (positive) sample collected in a time-frame between 2 weeks and 4 months from the end of definitive treatment (excluding adjuvant treatment). The window ≥14 days after treatment includes the landmark sample and all subsequent serial plasma samples, and patients were regarded as ctDNA-positive if ≥1 sample(s) was positive.

Sensitivity, specificity, positive predictive value (PPV), and negative predictive value (NPV) of ctDNA detection for recurrence prediction were calculated. Confidence intervals (CI) for sensitivity and specificity are provided as Clopper–Pearson CI, while CI for predictive values are provided as logit CI, except when the predictive value was 0 or 100%, in which case a Clopper–Pearson CI is provided. To evaluate the potential influence of clinical variables on ctDNA detection, a multivariable logistic regression analysis was performed to estimate odds ratios (ORs) with 95% confidence intervals (CIs). A concordance probability estimate (CPE) was calculated (excluding ties) to determine the extent to which stage and ctDNA status individually contribute to recurrence prediction. The chi-squared test was used to determine independence of observations between groups.

Kaplan–Meier curves were created for recurrence-free survival (RFS) and OS, and the log-rank test was used for comparison between groups. Hazard ratios (HRs) were calculated using Cox proportional hazards regression analyses. RFS was calculated from treatment end date to the date of recurrence of the first primary tumor, or time of death (if not preceded by a second primary tumor), whichever came first, or censored at most recent follow-up. OS was calculated from the treatment end date-to-date of death (by any cause) or censored at most recent follow-up. To account for guarantee-time bias (or immortal-time bias) [31], ctDNA status was treated as a time-dependent covariate for ‘landmark’ and ‘follow up’ (≥14 days) survival analyses. All analyses were executed in R version 4.0.4 using ‘ggplot’, ‘survival’ and ‘survminer’ packages [32–34].

## Findings

### Panel design and patient characteristics

Tissue-informed personalized panels were designed and validated for 130/134 treatment-naïve LEMA patients with stage 0–IIIC disease, for whom tumor specimens were available, including 118/121 (97.5%) patients with resected tissue material (three patients failed panel QC) and 12/13 (92.3%) for whom only biopsy material was available (one patient failed whole exome sequencing; S1 Fig and S1 Table). A median of 178 (interquartile range 122–303) SNVs per patient were identified by WES in tissue samples.

The 130 LEMA patients underwent surgery (90.8%), chemoradiotherapy (6.1%), or stereotactic radiotherapy (3.1%). Fifty (38%) received adjuvant treatment (S1 Table). Approximately half were diagnosed with stage I disease (52.3%) and the predominant histologic subtype was adenocarcinoma (68.5%; Table 1). Patients were followed for a median 1,250 days (until last follow up or death; range 50–2024), with 94%, 87% and 69% having at least 1, 2 or 3 years of observation respectively. Disease recurrence was reported in 42 patients (32.3%). These clinical characteristics were comparable to the LUCID cohort [29], and LEMA was considered as an independent validation cohort (Table 1).

**Table 1:** Clinical characteristics of the LEMA (*n* = 130), LUCID (*n* = 88), and combined cohorts (*n* = 218).

	LEMA cohort *N* = 130	LUCID cohort *N* = 88	Total cohort *N* = 218
Median age (years) at diagnosis, range	66 (44-82)	72 (44-88)	69 (44-88)
Sex, *n*			
Male	74 (56.9%)	45 (51.1%)	119 (54.6%)
Female	56 (43.1%)	43 (48.9%)	99 (45.4%)
Stage, *n*			
0	4 (3.1%)	0	4 (1.8%)
I	64 (49.2%)	43 (48.9%)	107 (49.1%)
II	24 (18.5%)	25 (28.4%)	49 (22.5%)
III	38 (29.2%)	20 (22.7%)	58 (26.6%)
Histology, *n*			
Adenocarcinoma	89 (68.5%)	55 (62.5%)	144 (66.1%)
Squamous cell carcinoma	29 (22.3%)	27 (30.7%)	56 (25.7%)
Other	12 (9.2%)	6 (6.8%)	18 (8.2%)
Treatment*, *n*			
Surgery	118 (90.8%)	69 (78.4%)	187 (85.8%)
Chemoradiotherapy	8 (6.1%)	18 (20.5%)	26 (11.9%)
Stereotactic radiotherapy	4 (3.1%)	1 (1.1%)	5 (2.3%)
Smoking status, *n*			
Never smoker	6 (4.6%)	8 (9.1%)	14 (6.4%)
Former smoker	85 (65.4%)	63 (71.6%)	148 (67.9%)
Smoker	39 (30.0%)	16 (18.2%)	55 (25.2%)
Unknown	0	1 (1.1%)	1 (0.5%)
Recurrence of the lung tumor	42 (32.3%)	30 (34.1%)	72 (33.0%)
Plasma time points, *n*	445	363	808
Baseline (pre-Tx)	87	78	165
Follow-up	359	285	644

*A statistically significant difference in treatment regimens was observed between the two cohorts (*p* < 0.05); fewer patients in LUCID had surgery and more had chemoradiotherapy as their definitive treatment. All other clinical characteristics assessed had similar prevalence (*p* > 0.05). Plasma time points include blood samples collected before (Baseline or pre-Tx), and at any time after (Follow-up) definitive treatment. ‘Other’ histology includes adenosquamous cell carcinoma, typical carcinoid, large cell neuroendocrine carcinoma of the lung, adenocarcinoma in situ, pleomorphic carcinoma, and ‘not otherwise specified’. *pre-Tx,  pre-treatment;* LEMA,  *Lung cancer Early Molecular Assessment* trial; LUCID,  *LUng Cancer CIrculating Tumour Dna Study.*

### Analysis of ctDNA before treatment

**Fig 1:**
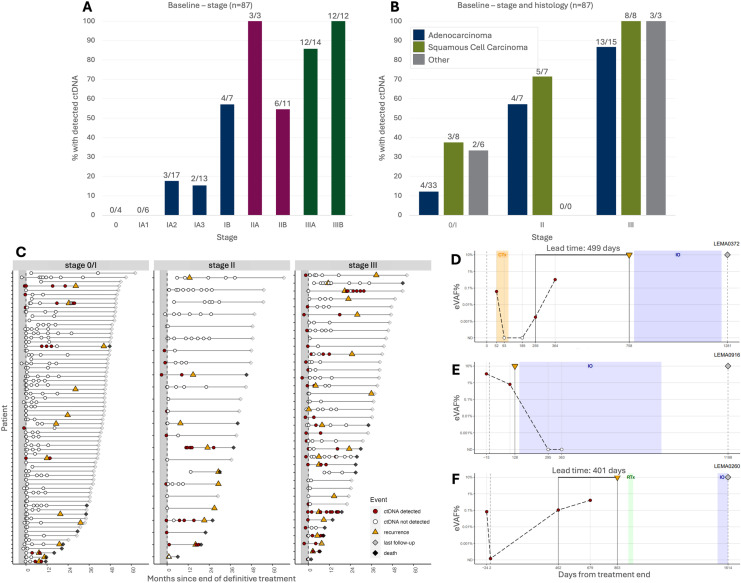
ctDNA detection in the LEMA cohort. **(A, B)** Histograms showing detection rates of ctDNA before treatment (*n* = 87), (A) according to disease stage and (B) according to disease stage and histological subtype. Detection rates are shown in percentages on the y-axis, and the number of samples in each group is indicated above the bars (detected/total). **(C)** Summary of longitudinal plasma monitoring in 130 LEMA patients (445 serial plasma samples) categorized by stage, indicating when ctDNA was detected (red points) or not detected (white points). Clinical recurrence is indicated with an orange triangle. Time is measured from end of curative treatment (day 0, vertical dashed black line) until end of follow-up (gray diamond) or death (black diamond). The gray shaded area indicates the pre-treatment time period. Equivalent figures with treatment annotation are provided in S2A–S2C Fig. LUCID collection is shown in S2D Fig. **(D–F)** Examples of longitudinal monitoring of ctDNA in plasma. (D) Patient LEMA0372 with stage IIIA adenocarcinoma treated with surgery followed by adjuvant chemotherapy. After recurrence palliative immunotherapy was started. (E) Patient LEMA0916 with stage IIIA squamous cell carcinoma treated with surgery only. Recurrence was observed after 128 days for which palliative immunotherapy was administered. (F) Patient LEMA0260 with stage IA squamous cell carcinoma treated with surgery. Ossal and lymph node recurrence was treated with stereotactic radiotherapy which led to disease control for over a year. However, liver metastases occurred and this resulted in the start of palliative immunotherapy. Longitudinal monitoring plots for all LEMA patients are included in S1 Appendix. The vertical dashed gray line indicates the end of definitive treatment. The dynamic dashed black line indicates the inferred trajectory of ctDNA kinetics over time. LEMA,  Lung cancer Early Molecular Assessment trial; LUCID, **LU**ng **C**ancer C**I**rculating Tumour **D**na Study; *CTx*, * chemotherapy; IO, immunotherapy; RTx, radiotherapy; eVAF, estimated Variant Allele Fraction.*

Preoperative ctDNA analysis was successfully performed for 87/99 (88%) patients from LEMA, with sufficient plasma available (S1C Fig). Before treatment, ctDNA was detected in 42 patients (48.3%), with a median eVAF of 0.021%. ctDNA detection rates rose with increasing stage; 0% (0/4) in stage 0, 20.9% (9/43) in stage I, 64.3% (9/14) in stage II, and 92.3% (24/26) in III (Fig 1A and S1 Table). Median eVAF similarly increased with stage; 0.012% (0.0017%−0.383%; stage I), 0.02% (0.002%−0.712%; stage II), and 0.06% (0.0008%−9.175%; stage III) (S1 Table). This is comparable to LUCID, where ctDNA was detected in 51.3% (40/78) of pre-treatment samples, including 24.4% (10/41), 77.3% (17/22), and 86.7% (13/15) of patients with stage I, II, and III [29]. In LEMA, detection was higher in patients with squamous cell carcinoma (SCC; 69.6%, 16/23) compared to patients with adenocarcinoma (38.2%, 21/55; Fig 1B and S4 Table), confirming previous observations [29,35]. Higher stage and squamous cell carcinoma were associated with a higher ctDNA detection rate pre-treatment, corrected for smoking history (S4 Table).

### Longitudinal monitoring of ctDNA to detect residual disease and predict recurrence

After treatment, a total of 358/386 (93%) plasma samples were successfully analyzed for MRD (average 2.8 samples per patient, range 1–9) (Figs 1C, S1C and S2A–S2D). ctDNA was detected post-treatment in LEMA in 17.6% (63/358) of samples and 21.3% (27/127) of patients, compared to 18.9% of samples (54/285) and 33.7% of patients (28/83) in LUCID [29].

Considering one landmark sample per patient (*n* = 82, collected a median 35 days with a range of 14–120 days after curative treatment), a sensitivity of 30.4% and specificity of 98.3% were observed in LEMA, with 87.5% PPV and 78.4% NPV. This was broadly comparable to LUCID (50.0%, 100%, 100%, and 78.7%, respectively; S5 Table and S2D Fig) [29]. For serial LEMA samples collected ≥14 days after treatment end, sensitivity improved to 60.5%, while specificity and PPV remained high at 97.4% and 92.0%. Again, LUCID results were similar with sensitivity, specificity and PPV of 64.3%, 95.9%, and 90.0% (S6 Table) [29]. In clinical care, it would be convenient to collect liquid biopsy samples early after surgery during hospital submission. However, in samples collected 1–3 days postoperatively, a low PPV of 33.3% for LEMA and 50% for LUCID was observed (S7 Table).

Given their consistency and reproducibility (S6 and S7 Tables), the LEMA and LUCID datasets were combined (Tables 1 and 2 and S2D Fig), affording greater statistical confidence. Considering both cohorts, the median follow-up was 1,161 days (range 22–2024), with 91%, 82%, and 59% having at least 1, 2, or 3 years of observation respectively. In patients with stage 0 and I disease, only 1 of 83 patients who did not develop a recurrence in the follow-up period had a positive ctDNA sample (*i.e.,* potential false positive), resulting in a PPV of 90.9% and specificity of 98.8%. Meanwhile, in 91 patients with stage II–III disease, there were 16 patients who had a recurrence and no ctDNA detected (*i.e.,* potential false negative), resulting in a NPV of 71.9% and sensitivity of 66.0%. In the subset of patients with ctDNA detected pre-treatment (*n* = 69), the sensitivity and NPV of post-treatment ctDNA to predict recurrence improved to 83.9% and 87.8% (S8 Table).

**Table 2:** Recurrence prediction by ctDNA status post-treatment, categorized in the landmark timeframe and serial samples collected from ≥14 days onwards, in the combined dataset*.

LEMA and LUCID combined*	ctDNA positive (*N*)		ctDNA negative (*N*)		Sens (%, *95% CI*)	Spec (%, *95% CI*)	PPV (%, *95% CI*)	NPV (%, *95% CI*)
	Relapse	No relapse^Σ^	No Relapse	Relapse^ς^				
**Landmark**^**+**^ **(≥14–122 days)**								
All stages (*N* = 139)	17	1	95	26	39.5	99.0	94.4	78.5
					*25.0, 55.6*	*94.4, 100*	*70.0, 99.2*	*74.3, 82.5*
Stage I (*N* = 81)^$^	3	0	66	12	20.0	100	100	84.6
					*4.3, 48.1*	*94.6, 100*	*29.2, 100*	*81.0, 87.6*
Stage II and III (*N* = 58)	14	1	29	14	50.0	96.7	93.3	67.4
					*30.7, 69.4*	*82.8, 99.9*	*66.3, 99.0*	*58.7, 75.1*
**Serial samples**^**+**^ **(≥14 days)**								
All stages (*N* = 193)	41	4	123	25	62.1	96.9	91.1	83.1
					*49.3, 73.8*	*92.1, 99.1*	*79.3, 96.5*	*78.3, 89.7*
Stage I (*N* = 102)^$^	10	1	82	9	52.6	98.8	90.9	90.1
					*28.9, 75.6*	*93.5, 100*	*57.7, 98.7*	*85.0, 95.2*
Stage II and III (*N* = 91)	31	3	41	16	66.0	93.2	91.2	71.9
					*50.7, 79.1*	*81.3, 98.6*	*77.3, 96.9*	*63.1, 79.4*

*Results of the combined LEMA and LUCID cohorts in the landmark (≥14–122 days post-treatment) and serial timeframe (≥14 days post-treatment) are presented in S5 and S6 Tables, respectively.

Σ Representing potential false positives.

ς Representing potential false negatives.

+ In the landmark timeframe, only one sample per patient, the first (positive) within 14–122 days after the treatment end date, was considered. In the serial analyses, a patient was regarded as ctDNA-positive if at least one sample ≥14 days after end of treatment was ctDNA positive.

$ Due to the small number, patients with stage 0 disease were grouped with patients with stage I patients.

*Sens, Sensitivity; Spec, Specificity; PPV, Positive Predictive Value; NPV, Negative Predictive Value; CI, Confidence Interval;* LEMA, Lung cancer Early Molecular Assessment trial; LUCID, **LU***ng* **C***ancer C***I***rculating Tumour* **D***na Study.*

**Fig 2:**
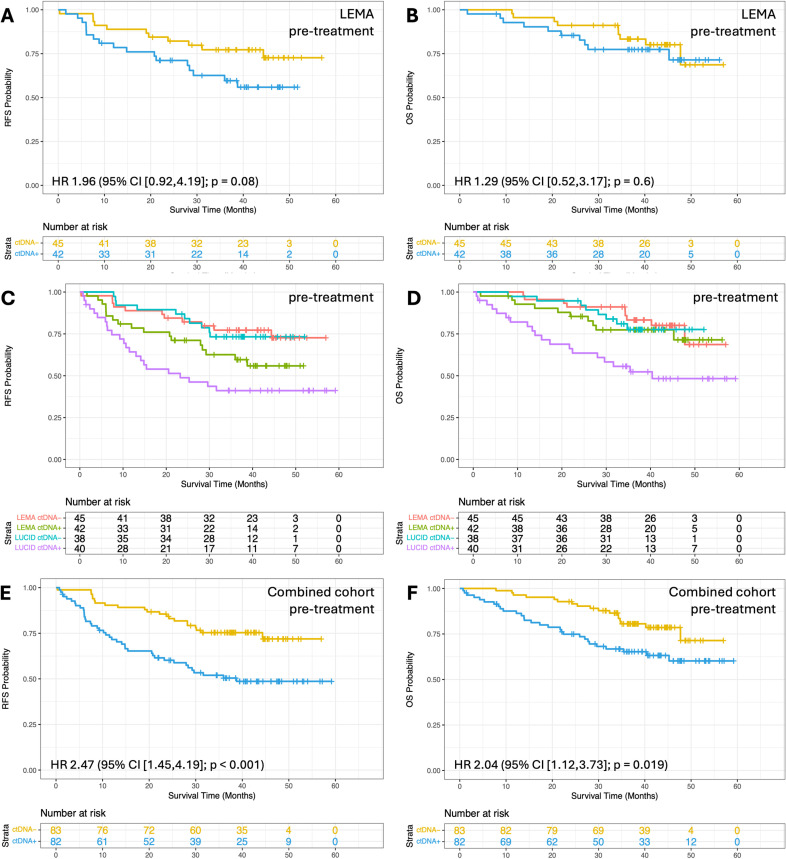
Survival analysis based on ctDNA detection pre-treatment in LEMA alone, and in the combined datasets. Kaplan–Meier analysis showing the fraction of patients without events as a function of time. Patient subgroups are defined based on ctDNA detection at different time windows. **(A)** Recurrence-free survival (RFS) and **(B)** Overall survival (OS) for LEMA patients with (blue line) or without (yellow line) ctDNA detected pre-treatment. The number of patients remaining at risk are shown below each graph (*n* = 87). **(C)** RFS and **(D)** OS for LEMA and LUCID cohorts. Patients with ctDNA detected are shown by purple (LUCID) or green (LEMA) lines, and those with ctDNA not detected are shown by blue (LUCID) or red (LEMA) lines (*n* = 87 and 78 for LEMA and LUCID respectively). **(E)** RFS and **(F)** OS for patients in the combined cohort (*n* = 165). All associations remained significant after accounting for guarantee time bias. LEMA, Lung cancer Early Molecular Assessment trial; LUCID, **LU**ng **C**ancer C**I**rculating Tumour **D**na Study; *HR,Hazard Ratio; CI, Confidence Interval; ctDNA+ indicates ctDNA detected; ctDNA− indicates ctDNA not detected.*

For 28 patients, ctDNA was detected in postoperative samples collected before recurrence, with a median lead time between detection and clinical recurrence of 206 days (6.8 months; examples of longitudinal monitoring in Fig 2D–2F). In 25 patients who developed recurrence with no ctDNA detected posttreatment, the median time from the last (negative) sample to recurrence was 262 days (8.6 months). We attempted to explore longitudinal ctDNA dynamics in the same vein as Zhang and colleagues [36], by considering *de novo* ctDNA detection in the months following landmark. However, we were limited in the number of patients with samples regularly available over an extended timeframe. We did, however, observe that amongst patients of any stage, there was a gradual increase in the proportion with ctDNA detected during a given period, with a peak at 12–18 months after landmark (though sample numbers after this were limited; S3A Fig). The same applied when focusing on just patients with stage II–III disease (those at highest risk of recurrence; S3B Fig). This 12–18 month period was highlighted by Zhang and colleagues as when the incidence of ctDNA detection and/or recurrence was at its highest, in their cohort [36].

### Survival analyses

Unlike LUCID [29], pre-treatment ctDNA detection in LEMA did not associate with reduced RFS or OS (HR 2.0 (95% CI [0.9,4.2]; *p* = 0.08) and HR 1.3 (95% CI [0.5,3.2]; *p* = 0.6), respectively; Fig 2A–2D). Whilst the association held when combining the cohorts (RFS; HR 2.5 (95% CI [1.5,4.2]; *p* < 0.001) and OS; HR 2.0 (95% CI [1.1,3.7]; *p* = 0.02)); Fig 2E and 2F), it was not significant upon multivariable analysis (S4A and S4B Fig). Aligning with recent findings[23,37,38], we found that even in patients with very low levels of ctDNA (here considering eVAF <0.01% or <0.008% [37]), RFS and OS were significantly worse, as compared to patients in which no ctDNA was detected (For eVAF < 0.01%, RFS; HR 2.5 (95% CI [1.3,5.0]; *p* = 0.01) and OS; HR 2.3 (95% CI [1.1,4.9]; *p* = 0.031). For eVAF < 0.008%, RFS; HR 2.5 (95% CI [1.2,5.3]; *p* = 0.013) and OS; HR 2.0 (95% CI [0.9,4.6]; *p* = 0.11). S5A–S5D Fig), according to univariate analysis. This applied when assessing all patients, or just those with lung adenocarcinoma (For <0.01% eVAF, RFS; HR 3.3 (95% CI [1.6,7.0]; *p* = 0.002) and OS; HR 3.4 (95% CI [1.5,7.8]; *p* = 0.003). S5E and S5F Fig).

**Fig 3:**
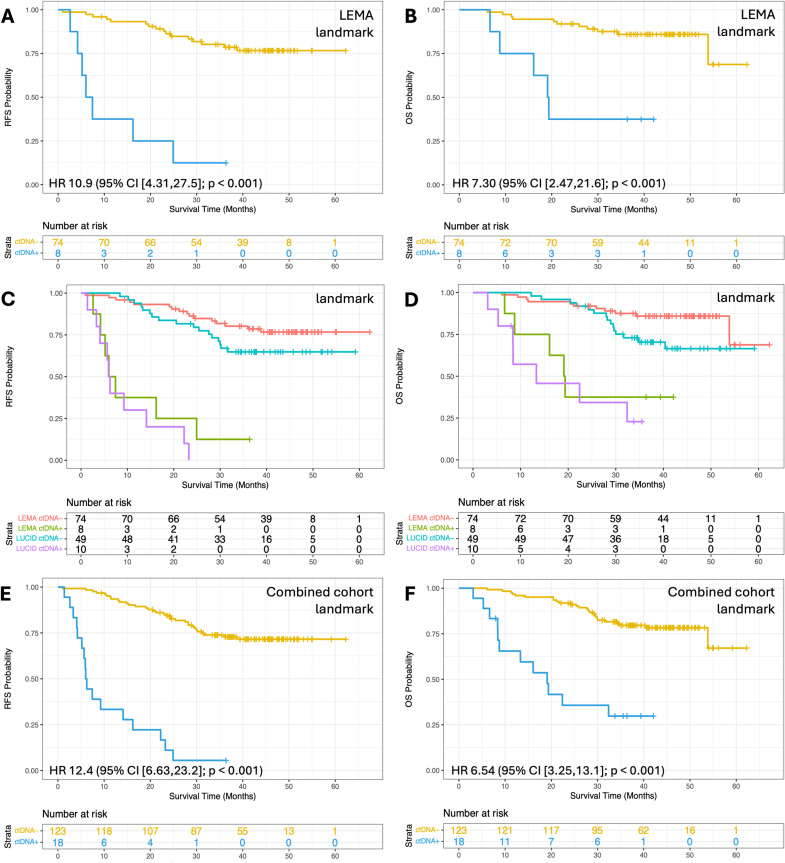
Survival analysis based on ctDNA detection at landmark in LEMA alone, and in the combined datasets. **(A)** Recurrence-free survival (RFS) and **(B)** Overall survival (OS) for LEMA patients split by ctDNA detection at the landmark time point, which is the first plasma sample available in the window of ≥2 weeks and ≤4 months after the end of curative treatment (*n* = 82). **(C)** RFS and **(D)** OS for LEMA and LUCID cohorts. Patients with ctDNA detected are shown by purple (LUCID) or green (LEMA) lines, and those with ctDNA not detected are shown by blue (LUCID) or red (LEMA) lines (*n* = 82 and 59 for LEMA and LUCID respectively). **(E)** RFS and **(F)** OS for patients in the combined cohort (*n* = 141). All associations remained significant after accounting for guarantee time bias. LEMA, Lung cancer Early Molecular Assessment trial; LUCID, **LU**ng **C**ancer C**I**rculating Tumour **D**na Study; *HR, Hazard Ratio; CI, Confidence Interval; ctDNA+ indicates ctDNA detected; ctDNA− indicates ctDNA not detected.*

**Fig 4:**
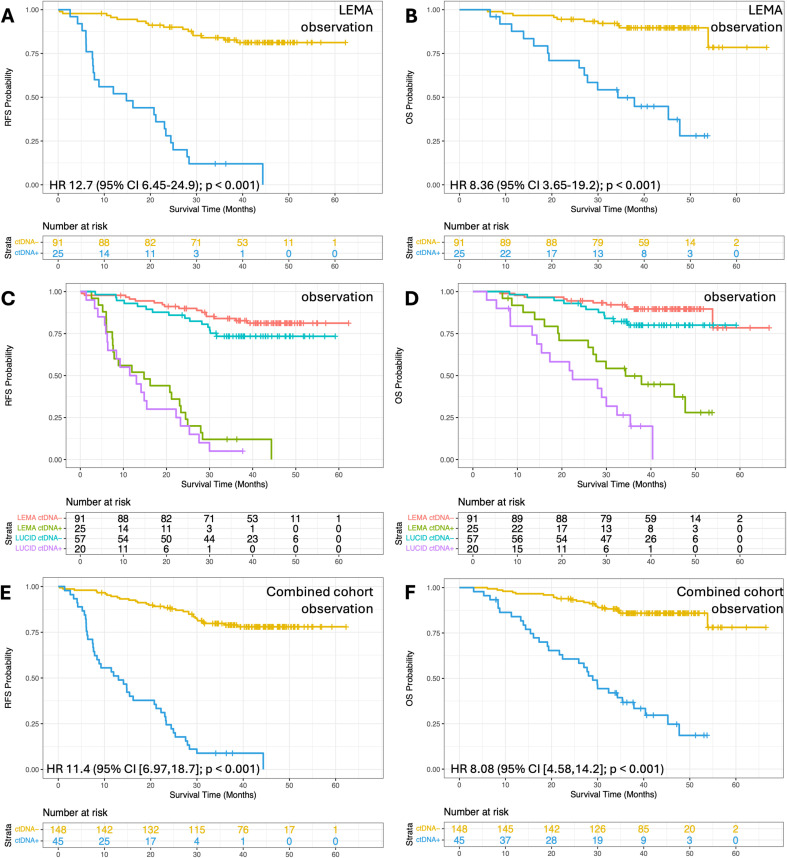
Survival analysis based on ctDNA detection during observation in LEMA alone, and in the combined dataset. **(A)** Recurrence-free survival (RFS) and **(B)** Overall survival (OS) for LEMA patients split by ctDNA detection at any time point ≥14 days after the end of curative treatment (*n* = 116). **(C)** RFS and **(D)** OS for LEMA and LUCID cohorts. Patients with ctDNA detected are shown by purple (LUCID) or green (LEMA) lines, and those with ctDNA not detected are shown by blue (LUCID) or red (LEMA) lines (*n* = 116 and 77 for LEMA and LUCID respectively). **(E)** RFS and **(F)** OS for patients in the combined cohort (*n* = 193). All associations remained significant after accounting for guarantee time bias. LEMA, Lung cancer Early Molecular Assessment trial; LUCID, **LU**ng **C**ancer C**I**rculating Tumour **D**na Study; *HR, Hazard Ratio; CI, Confidence Interval; ctDNA+ indicates ctDNA detected; ctDNA− indicates ctDNA not detected.*

Considering the combined cohorts, the difference in RFS and OS was more evident in samples collected within 1−3 days post-treatment (RFS; HR 8.6 (95% CI [3.8,19.6]; *p* < 0.001) and OS; HR 11.2 (95% CI [4.2,30.2]; *p* < 0.001); S6 Fig), and at the landmark time point (RFS; HR 12.4 (95% CI [6.63,23.2]; *p* < 0.001) and OS; HR 6.5 (95% CI [3.3,13.1]; *p* < 0.001); Fig 3). The latter was consistent between LEMA and LUCID (Fig 3C and 3D), and remained significant upon multivariable analysis (S4C and S4D Fig). In samples collected ≥14 days after treatment, a comparable reduction in RFS and OS was observed (HR 11.4 (95% CI [7.0,18.7]; *p* < 0.001) and HR 8.1 (95% CI [4.6,14.2]; *p* < 0.001), respectively; Fig 4E and 4F). This observation was again consistent between LEMA and LUCID (Fig 4A–4D), and held after multivariable analysis (S4 and S4F Fig), and across disease stages (S7 Fig), and when comparing adenocarcinoma versus SCC (S8 Fig). CPE analysis was used to determine the relative contribution of ctDNA status and disease stage, highlighting the importance of both metrics for recurrence prediction (S9 Table). In pre-treatment ctDNA-positive patients, subsequent ctDNA detection post-treatment differentiated patients with or without recurrence even better (RFS: HR 25.4 (95% CI [9.6,67.1]; *p* = 2,6^−17^); OS: HR 10.5 (95% CI [3.5,31.3]; *p* = 1,7^−7^); S9 Fig). Interestingly, pre-treatment ctDNA status was not prognostic in those patients that were landmark negative (S10 Fig). Whilst ctDNA detection (i.e. ctDNA positive versus negative) associated with survival, ctDNA levels (eVAF) in positive samples did not correlate with survival outcomes, at either pre-treatment or landmark time points (S10 Table).

### Potential false positive and negative predictions of recurrence

In the combined dataset, ctDNA was detected during monitoring in four patients that did not develop recurrence. In two patients, the positive ctDNA sample was collected before or during adjuvant treatment which may have cured their disease, leading to the apparent ‘false positive’ result. When analyzing only serial samples collected after adjuvant treatment, PPV and specificity of recurrence prediction improved to 95.1% and 98.2% (S11 Table).

In 25 patients that developed recurrence but remained ctDNA-negative, we investigated analytical and clinical parameters to explain the ‘false negativity’. Sample and assay quality were ruled out (S11 Fig). Analysis of the time interval between samples and recurrence showed that the proportion of ‘false negative’ ctDNA samples remained the same when limiting to samples collected within 3 or 6 months before recurrence (S12 Table). It was noted that amongst the 17 (of 25) patients that had a pre-treatment ctDNA result, 6 (35%) were ctDNA positive. This compares with a pre-treatment ctDNA positivity rate of 54% in patients that recurred who had a positive post-treatment result (i.e., ‘true positives’). This did not represent a significant difference (*p* > 0.05), though was based on small numbers.

Pathological data showed that patients who developed a recurrence but remained ctDNA-negative, more often had lepidic (42%) or acinar subtypes (42%) compared to ctDNA-positive patients who more often displayed solid (24%) or micropapillary tumors (14%) (*p* = 0.024; S13 Table). Interestingly, no difference was observed in ctDNA detection with regard to tumor diameter or volume when corrected for stage (*P* > 0.05; S13 Table and S12 Fig).

## Discussion

The key study aim was to validate a personalized tissue-informed ctDNA assay for NSCLC recurrence prediction in an independent cohort. Our findings surprised us in the extent and detail of consistency and reproducibility between retrospectively assessed, real-world cohorts from different countries, analyzed years apart (S5–S7 Table), confirming the performance of the assay, and ctDNA as a biomarker, to predict recurrence. When combining cohorts, post-treatment detection of ctDNA was associated with a 11.4-fold higher risk of recurrence and 8.1-fold higher risk of death, underlining the potential of ctDNA to differentiate patients with a high versus low risk of recurrence and survival. In patients with stage I disease, high specificity (100%) and PPV (100%) at landmark were observed, supporting the addition of adjuvant treatment in ctDNA-positive patients. In patients with stage II–III disease, in whom ctDNA was detected before, but not after treatment, the NPV was 88%, which, whilst encouraging, is currently not high enough to signal de-escalation of adjuvant treatment, as demonstrated in early-stage colon cancer [39] Overall, these results show the potential role of ctDNA to guide and support adjuvant treatment decisions.

Though the field is rapidly evolving, the general approach to MRD ctDNA testing can be categorized as either tumor-informed or tumor-agnostic. In theory, and as demonstrated by a recent study [24], tumor-informed assays benefit from improved analytical sensitivity, and associated prognostic implications. Indeed, our results, generated using the tumor-informed RaDaR assay, showed improved clinical performance compared to previous studies of tumor-agnostic ctDNA detection after surgery, that reported specificity ranging from 78% to 98%, and PPVs ranging from 63% to 90% [20,22,24,27,28,36,40]. In recent years, tumor-informed assays have been increasingly applied to early-stage NSCLC (summarized in S14 Table). Though they have shown variable sensitivity, specificity, PPV and NPV for recurrence prediction, direct comparison is challenging due to the diversity of study design and size, patient demographics, treatment, and analysis methods. Some studies have recruited large numbers of patients (>150) [24,36,37,41], and/or have collected samples prospectively [21,23,24,42], and so a clearer picture is emerging of the clinical utility of ctDNA. Also of note, others (in particular the team behind TRACERx, [e.g., 23,35,37,38,43]) have yielded fascinating and impactful biological insights to NSCLC and the utility of ctDNA, including for example, shifts in the clonal composition of relapsing disease [23].

The timing of post-operative blood sampling should be considered, to ensure that MRD testing is both effective and practical. Samples collected 1–3 days after treatment, when patients are still in hospital, would be clinically advantageous. However, whilst able to stratify patients by RFS and OS, samples collected during this timeframe had a PPV of only 53% for recurrence detection, underlining the hypothesis that MRD analysis is best delayed beyond the first 1–2 weeks after resection, due to excess trauma-induced cfDNA [44]. When investigating samples taken ≥14 days after the treatment end date, higher specificity and PPV (97% and 91%) were observed.

A limitation of our study is the variable sample collection schedule, during routine follow-up visits. Whilst this may limit the generalizability of our results, one of the main advantages of our study is that it comprises a real-world cohort which was treated with conventional treatment, according to clinical guidelines. Therefore, the results of our cohort may be better generalizable to real-world practice, as compared to prospective studies, where strict inclusion criteria are often used. Relevant to real-world practice, we are encouraged by the success rate of WES and panel creation across the tissue samples collected from LEMA and LUCID. Despite the consensus that tissue availability and quality may pose a challenge to the use of tissue-informed assays, we observed that sufficient tissue was available and evaluable for WES in 191/191 (100%) of surgical resection specimens, and in 31/33 (94%) of diagnostic biopsies across both cohorts.

Patients who recurred but remained ctDNA negative had a median 262 days between the last sample and radiological progression, potentially explaining ctDNA negativity in a subset of patients with recurrence. However, it is noteworthy that the proportion of ctDNA-negative patients remained the same when restricting analysis to samples collected within 6 or 3 months of recurrence (S12 Table). Alternatively, quantities of ctDNA shed into the peripheral bloodstream could vary based on tumor biological characteristics. We observed lower pre-treatment ctDNA detection in these patients, as compared to those that recurred and were ctDNA positive post-treatment (albeit that this did not represent a significant difference, likely due to low numbers). In addition to confirming lower detection in adenocarcinomas versus SCC, we detected ctDNA more often in histologically aggressive tumors, including micropapillary [45] and solid tumors, while, contrary to previous data [35,36], tumor diameter was not associated with detection (S13 Table). Finally, it is possible that these patients underwent treatment-induced, clonal evolution of their tumor that introduced new resistant clones with a different genomic landscape. However, the variants selected for panel design are likely to represent, or will at least be enriched for, clonal variants that would be expected to persist in such emerging clones.

High clinical specificity for prediction of recurrence (97%) may enable monitoring of MRD by repeated testing, increasing the chance of detecting ctDNA. Sensitivity increased from 38% to 62% when considering one landmark sample versus all follow-up samples. Furthermore, when restricting analysis to patients in whom ctDNA was detected before treatment, detection post-treatment in patients with recurrence improved further (sensitivity 84%), associated with a 25.4-fold higher risk of recurrence.

Based on these results, we believe that guidance of additional treatment after intended curative treatment, by ctDNA analysis, is feasible and can be clinically impactful. This aligns with emerging data showing that ctDNA can be prognostic, e.g., as suggested by ctDNA clearance data from the IMpower010 trial in which patients were treated with atezolizumab [46,47], and in early real-world data [42]. Furthermore, given its high specificity and PPV, a positive ctDNA result has the potential to predict patients most likely to benefit from the increasingly efficacious adjuvant treatments used as standard of care [8,9], including osimertinib [41,48] and immunotherapies (such as atezolizumab, as above [10,11]). With the arrival of more neoadjuvant/perioperative treatment options, there is a potential for overtreatment in patients who may have already been cured prior to adjuvant treatment. How can we identify those patients with residual disease that might merit adjuvant personalized treatment? We believe that highly sensitive ctDNA-MRD technology, such as that used here, might help us to target adjuvant therapy only to the high-risk group, thereby preventing unwarranted toxicity and unnecessary treatments. If patients are immediately treated based on a positive ctDNA result, valuable time could be saved, allowing patients to start treatment in better health. In instances of landmark test negativity, a surveillance approach could be adopted to identify patients that might not need systemic treatment, since test conversion occurs well in advance of radiological recurrence. In order to move from “risk-based” towards “targeted” adjuvant treatment, well-designed, adequately powered, prospective clinical trials in which patients are under surveillance and receive additional treatment based on a positive ctDNA result are needed [49]. These will inform national guidelines with high-quality evidence.

It is also noteworthy that ctDNA detection and dynamics have equal promise for the monitoring and management of neoadjuvant/perioperative treatment of NSCLC [12,50–53].In CheckMate 816, ctDNA clearance was shown to be an early predictor of favorable outcomes after treatment with nivolumab, and was more often detected in patients that achieved a pathological complete response (pCR) [12]. In the AEGEAN study, ctDNA clearance during neoadjuvant treatment with durvalumab and chemotherapy was demonstrated to be associated with both pathological response and event-free survival, and that absence of ctDNA clearance may identify patients unlikely to achieve pCR [52,53]. Here, only two patients (one from LEMA and one from LUCID) received neoadjuvant chemo(radio)therapy. Neither had ctDNA detected, though sampling was limited.

We hypothesize that the subset of patients that recurred but remained ctDNA negative, harbor biologically less aggressive tumors that shed less ctDNA [36], and this is reflected by a potentially lower pre-treatment positivity rate. Missing these patients in MRD detection may not worsen survival outcomes, but this should be confirmed in prospective clinical trials as well. It is likely that more of these patients will be identified as ctDNA-positive post-surgery, as assays become increasingly sensitive, as suggested by recent data comparing an assay based on PhasED-Seq (LoD95 determined as 1 parts per million; clinical sensitivity at landmark of 67%) versus CAPP-Seq (LoD95 determined as 84 parts per million; clinical sensitivity at landmark of 28%) at ~100% specificity [54]. ctDNA detection after surgery/radiotherapy can also support adjuvant clinical trials that would enroll only the subset of patients at high risk of relapse, increasing effect sizes and reducing the numbers needed for statistical power.

In summary, our study provides evidence that ctDNA detection after definitive treatment in patients with early-stage NSCLC, by a personalized tissue-informed assay, indicates relapse in patients who may subsequently benefit from additional treatment. Demonstrated across independent cohorts, ctDNA-based MRD analysis has potential to provide clinical decision support and enhance patient survival outcomes.

## Supporting information

S1 Table Clinical data and ctDNA summary data for the 130 patients in the LEMA study. *NA indicates data Not Available; ND indicates Not Detected*.
(XLSX)

S2 Table Variants included in the RaDaR panels for all 130 LEMA patients. (XLSX)

S3 Table ctDNA RaDaR data for all 445 time points for all 130 LEMA patients. (XLSX)

S4 Table Multivariable logistic regression analysis for ctDNA detection pre-treatment. Multivariable logistic regression analysis for ctDNA detection pre-treatment (*n* = 165). Explored variables include histology, gender, smoking status and stage category, with output presented as odds ratio and 95% confidence intervals. *CI, Confidence Interval.*
(DOCX)

S5 Table Recurrence prediction and landmark ctDNA detection. Recurrence prediction by ctDNA detection in the landmark timeframe in the combined (*N* = 139), LEMA (*N* = 82) and LUCID (*N* = 57) cohorts. The landmark timeframe includes the first (positive) sample collected between 2 weeks and 4 months from the end of treatment. *A patient was regarded as ctDNA-positive if at least one sample in the specified time window was positive for ctDNA. Due to the small number, patients with stage 0 disease were grouped with patients with stage I disease. ^**Σ**^Representing potential false positives. ^**ς**^Representing potential false negatives. *Sens, Sensitivity; Spec,  Specificity; PPV, Positive Predictive Value; NPV, Negative Predictive Value; CI, Confidence Interval.*
(DOCX)

S6 Table Recurrence prediction and longitudinal ctDNA detection (≥14 days post-treatment). Recurrence prediction by ctDNA detection in serial samples collected ≥14 days post-treatment, in the combined cohort (*N* = 193), LEMA (*N* = 116) and LUCID (*N* = 77) cohort. *A patient was regarded as ctDNA-positive if at least one sample in the specified time window was positive for ctDNA. Due to the small number, patients with stage 0 disease were grouped with patients with stage I disease. ^**Σ**^Representing potential false positives. ^**ς**^Representing potential false negatives. *Sens, Sensitivity; Spec, Specificity; PPV, Positive Predictive Value; NPV, Negative Predictive Value; CI, Confidence Interval.*
(DOCX)

S7 Table Recurrence prediction and ctDNA detection 1–3 days after curative treatment. Recurrence prediction by ctDNA detection post-treatment when including only samples obtained 1–3 days after the end date of the curative intended treatment in the combined (*N* = 80), LEMA (*N* = 32) and LUCID (*N* = 48) cohort. *A patient was regarded as ctDNA-positive if at least one sample in the specified time window was positive for ctDNA. Due to the small number, patients with stage 0 disease were grouped with patients with stage I disease. ^**Σ**^Representing potential false positives. ^**ς**^Representing potential false negatives. *Sens, Sensitivity; Spec, Specificity; PPV, Positive Predictive Value; NPV, Negative Predictive Value; CI, Confidence Interval.*
(DOCX)

S8 Table Recurrence prediction and ctDNA detection ≥14 days post-treatment in pre-treatment positive patients. Recurrence prediction by ctDNA detection ≥14 days post-treatment in patients that were ctDNA positive pre-treatment. *A patient was regarded as ctDNA-positive if at least one sample in the specified time window was positive for ctDNA. In the landmark timeframe, only one sample per patient, the first (positive) sample within 2 weeks to 4 months after the treatment end date, was considered. ^**Σ**^Representing potential false positives. ^**ς**^Representing potential false negatives. *Sens, Sensitivity; Spec, Specificity; PPV, Positive Predictive Value; NPV, Negative Predictive Value; CI, Confidence Interval.*
(DOCX)

S9 Table Exploration of relative contribution of ctDNA status and disease stage to the recurrence prediction. Concordance Probability Estimate (CPE) analysis to determine the relative contribution of ctDNA status and disease stage to the recurrence prediction ability at different time points. *OS, Overall Survival; RFS, Recurrence Free Survival.*
(DOCX)

S10 Table Baseline and landmark ctDNA eVAF, and survival outcomes. Assessment of effect of eVAF at baseline in ctDNA positive samples, and landmark on survival outcomes. *OS, Overall Survival; RFS, Recurrence Free Survival*.
(DOCX)

S11 Table Exploration of the effect of adjuvant treatment on survival analysis. Exploration of the effect of exclusion (i.e., treating end of curative treatment as *T* = 0) or inclusion (i.e., treating end of adjuvant treatment as *T* = 0) of adjuvant treatment in the analysis. *In the landmark timeframe, only one sample per patient, the first (positive) within 14–122 days after the treatment end date, was considered. In the serial analyses, a patient was regarded as ctDNA-positive if at least one sample ≥14 days after end of treatment was ctDNA positive. ^Σ^Representing potential false positives. ^ς^ Representing potential false negatives. *Sens, Sensitivity; Spec, Specificity; PPV, Positive Predictive Value; NPV, Negative Predictive Value; CI, Confidence Interval.*
(DOCX)

S12 Table Analysis of time interval between samples and recurrence. Analysis of the time interval between sample collection and disease recurrence. Shown are the number of events to occur within a specific time frame relative to recurrence; 1, at any time relative to recurrence; 2, within 6 months prior to recurrence; 3, within 3 months prior to recurrence.
(DOCX)

S13 Table Exploration of clinical and biological characteristics in patients that did or did not recur, and ctDNA detection ≥14 days post-treatment. Characteristics of patients with and without recurrence, categorized by ctDNA prediction of recurrence ≥14 days post-treatment. Statistical analyses was performed using the Pearson Chi-Squared Test, or when appropriate the Fisher–Freeman–Halton Exact Test, for categorical variables and the independent samples *T* test for nominal variables. Statistical analyses were not performed for patients without recurrence because of a sample size ≤5 patients. We acknowledge that numbers for these analyses are small and therefore these findings should be interpreted with caution. *Within both LEMA and LUCID cohorts, on occasion mixed histological subtypes were observed within the same tumor (e.g., an adenocarcinoma with both lepidic and acinar components) (*N* = 7). For LEMA, the relative composition of these tumors was known, allowing for determination of the predominant subtype and inclusion of these cases based on that subtype. For the LUCID cohort, this data was not available and, as such, the cases with mixed histological subtypes were excluded from this analysis. ^*AIS,  adenocarcinoma* in situ*; MIA, minimally invasive adenocarcinoma*. ^+^Additional information about the location of metastasis was available only for the LEMA cohort. ^&^Other locations of metastases include kidney, liver or multiple locations.
(DOCX)

S14 Table Summary of select studies of ctDNA utility for longitudinal (post-treatment) MRD detection. Summary of studies of post-treatment ctDNA in patients with early-stage NSCLC, and its predictive ability for recurrence detection. Studies are split by assay type; either tumor-informed or tumor-agnostic. Sensitivity, specificity, positive predictive value (PPV), and negative predictive value (NPV) are provided for each study (patient number shown in parenthesize), as well as median lead time from initial ctDNA detection to recurrence. Some values are inferred or omitted where data is unavailable or unclear. Some data are based on conference proceedings, without peer-review, and so should be interpreted with caution. This list is not exhaustive, but highlights the wide array of studies and assays that have explored ctDNA in this setting, and the diversity in predictive performance (though many values are based on relatively small numbers). References are provided at the end of this Supporting information document. *PPV, Positive Predictive Value; NPV, Negative Predictive Value.*
(DOCX)

S1 Fig **(A)** Study design. **(B)** Flow diagram depicting the selection of the MRD patient cohort. **(C)** Flow diagram depicting the LEMA patient cohort, sample availability, and results of ctDNA analysis, categorized by clinical outcome.
(PDF)

S2 Fig Longitudinal plasma monitoring in (A) stage 0 and I, (B) stage II, and (C) stage III LEMA patients. **(D)** Plasma monitoring in LEMA and LUCID, split by stage. Figures indicate when ctDNA was detected (red points) or not detected (white points). Clinical recurrence is indicated with an orange triangle. Time is measured from end of curative treatment (day 0) until end of follow-up (gray diamond). Treatment periods and type are indicated by coloured highlights.
(PDF)

S3 Fig Summary of ctDNA detection in the months following landmark, in patients with any stage **(A)** or stage II–III **(B)** disease. Time is considered as 6-month windows after landmark (0–6 months, 6–12 months, etc.). The top row indicates the raw count of patients with at least one ctDNA positive sample (red) or no ctDNA positive sample(s) (white), regardless of whether ctDNA and/or recurrence had occurred before that point. The bottom row shows the same data as a percentage of the total number of patients.
(PDF)

S4 Fig Cox regression analysis exploring the association of multiple covariables with survival. Associations are depicted as forest plots with hazard ratio (HR), 95% confidence interval and *p*-values indicated for each variable. The number of observations in each ‘category’ are indicated in parentheses. Data are based on the combined LEMA and LUCID cohorts. Multivariable analysis of clinical covariates and ctDNA detection at baseline (pre-treatment), with **(A)** recurrence-free survival (RFS) and **(B)** overall survival (OS). The equivalent data are presented considering detection at landmark **(C, D)**, and all longitudinal sampling **(E, D)**.
(PDF)

S5 Fig Survival analysis based on pre-treatment ctDNA detection at low eVAF vs. ctDNA not detected. Kaplan–Meier analysis showing the fraction of patients without events as a function of time. Patient subgroups are defined based on ctDNA detection at eVAF <0.01% or <0.008% (blue) vs. ctDNA not detected (yellow) at the pre-treatment time point. The number of patients remaining at risk are shown below each graph. **(A)** Recurrence-free survival (RFS) and **(B)** overall survival (OS) for combined LEMA and LUCID patients split by ctDNA detection at eVAF < 0.01% vs. ND. **(C, D)** eVAF < 0.008% vs ND. **(E, F)** eVAF < 0.01% in LUAD patients only. *ND, not detected; LUAD, lung adenocarcinoma.*
(PDF)

S6 Fig Survival analysis based on ctDNA detection in plasma collected within 1–3 days after curvative treatment. Kaplan–Meier analysis showing the fraction of patients without events as a function of time. Patient subgroups are defined based on ctDNA detection in samples collected within 1–3 days after curative treatment. Patients with ctDNA detected are shown by blue lines, and those with ctDNA not detected are shown by yellow lines. The number of patients remaining at risk are shown below each graph. **(A)** Recurrence-free survival (RFS) and **(B)** overall survival (OS) for LEMA patients split by ctDNA detection within 1–3 days after the end of curative treatment. **(C, D)** for LUCID patients. **(E, F)** for the combined cohorts.
(PDF)

S7 Fig Survival analysis based on ctDNA detection categorized by stage in the combined dataset. Recurrence free survival analysis of the combined cohort for patients split by ctDNA detection at any time point ≥2 weeks after the end of curative treatment, categorized by stage 0/I (**A**, *n* = 98), stage II (**B**, *n* = 39) and stage III (**C**, *n* = 52). Shown are equivalent data for Overall survival analysis **(D, E, F)**, respectively.
(PDF)

S8 Fig Survival analysis based on ctDNA detection categorized by tumor histology. Recurrence survival analysis of the combined cohort for patients split by ctDNA detection at pretreatment **(A, B)**, or at any time point ≥2 weeks after the end of curative treatment **(C, D)**, categorized by tumor histology; Adenocarcinoma (A, *n* = 102; C, *n* = 132), Squamous Cell Carcinoma (B, *n* = 48; D, *n* = 47). The equivalent Overall survival analysis data are also shown, with ctDNA detection at pretreatment **(E, F)**, or at any time point ≥2 weeks after the end of curative treatment **(G, H)**, categorized by Adenocarcinoma (E, *n* = 102; G, *n* = 132), Squamous Cell Carcinoma (F, *n* = 48; H, *n* = 47).
(PDF)

S9 Fig Survival analysis based on ctDNA detection in patients with positive ctDNA pretreatment in the combined dataset. Kaplan–Meier analysis showing the fraction of patients without events as a function of time. Patient subgroups are defined based on ctDNA detection at different time windows. Patients with ctDNA detected are shown by blue lines, and those with ctDNA not detected are shown by yellow lines. The number of patients remaining at risk are shown below each graph. **(A)** RFS and **(B)** OS including only patients with positive ctDNA pretreatment, split by ctDNA detection at any time point ≥14 days after the end of curative treatment (*n* = 69). **(C)** RFS and **(D)** OS including only patients with positive ctDNA pretreatment, split by ctDNA detection at the landmark time point, which is the first plasma sample available in the window of ≥2 weeks and ≤4 months after the end of curative treatment (*n* = 49).
(PDF)

S10 Fig Survival analysis of the predictive value of pretreatment ctDNA in patients that were ctDNA negative at the landmark time point in the combined dataset. **(A)** RFS and **(B)** OS stratified by, split by ctDNA detection at the landmark and pretreatment time points (*n* = 105).
(PDF)

S11 Fig Theoretical ctDNA detection limits. Evaluation of tumor variants that were successfully included in the ctDNA panel vs. the assay input in copies. The dashed vertical lines indicate the minimum (2,000 copies) and maximum (20,000 copies) input amounts for RaDaR. The dashed horizontal lines indicate the minimum (8) and maximum (48) number of variants targeted per panel. Diagonal lines represent the lower limit of detection (LoD). Only samples of patients with negative ctDNA while developing recurrence (i.e., potential false negative samples; FN, represented by the red points) and samples of patients with positive ctDNA while not developing recurrence (i.e., potential false positive samples; FP, represented by the blue points) are shown. **(A)** Samples included in the landmark timeframe. **(B)** Samples collected during the follow-up period starting ≥14 days after the end date of curative treatment.
(PDF)

S12 Fig Comparison of tumor volume with ctDNA levels at baseline. Exploration of the relationship between tumor volume (mm3) and ctDNA levels (eVAF, %) at baseline. Note, tumor volumetric data were only available for the LUCID cohort. Disease stage is indicated by point color.
(PDF)

S1 Methods Additional information concerning sample collection, tissue processing and analysis by whole exome sequencing, and RaDaR personalized ctDNA sequencing assay analysis. Also includes a discussion of the value of tumor-informed vs. tumor-agnostic approaches to ctDNA detection, and impact on clinical performance.
(DOCX)

S1 Appendix Longitudinal time course figure for each patient in the LEMA cohort with plasma ctDNA data. Figures are grouped according to detection status during the observation timeframe (≥days post-treatment), and how that related to recurrence status, i.e., true positives, false negatives, false positives, and true negatives.
(PDF)

S1 Text Appendix providing the full list of collaborators, and members of the LUCID and LEMA study groups. (DOCX)

## Abbreviations

cfDNA: cell-free DNA
CI: confidence interval
CPE: concordance probability estimate
ctDNA: circulating tumor DNA
CTx: Chemotherapy
eVAF: estimated variant allele fraction
FFPE: formalin-fixed paraffin-embedded
HR: hazard ratio
IO: immunotherapy
LEMA: **L**ung cancer **E**arly **M**olecular **A**ssessment trial
LUAD: lung adenocarcinoma
LUCID: **LU**ng **C**ancer C**I**rculating Tumour **D**na study
MRD: molecular residual disease
ND: not detected
NPV: negative predictive value
NSCLC: non-small cell lung cancer
OR: odds ratio
OS: overall survival
pCR: pathological complete response
PPV: positive predictive value
Pre-Tx: pre-treatment (baseline)
RFS: recurrence-free survival
RTx: radiotherapy
Sens: sensitivity
SCC: squamous cell carcinoma
Spec: specificity
SNV: single nucleotide variant
WES: whole exome sequencing
