# Supplementary material for: Recurrence prediction using circulating tumor DNA in patients with early-stage non-small cell lung cancer after treatment with curative intent: A retrospective validation study
Source: PLoS Med. 2025 Apr 15;22(4):e1004574. doi: 10.1371/journal.pmed.1004574 (PMC12021277; doi:10.1371/journal.pmed.1004574)
Supplement: S4 Table — Multivariable logistic regression analysis for ctDNA detection pre-treatment (n = 165). Explored variables include histology, gender, smoking status and stage category, with output presented as odds ratio and 95% confidence intervals. CI, Confidence Interval. (DOCX) [file pmed.1004574.s004.docx]

**S4 Table** Multivariable logistic regression analysis for ctDNA detection pre-treatment

|  | **Odds ratio** | **Lower limit 95% CI** | **Upper limit 95% CI** |
| --- | --- | --- | --- |
| Gender (female) | 0.6 | 0.2 | 1.4 |
| Histology |  |  |  |
| Adenocarcinoma (reference) | 1.0 | - | - |
| Squamous cell carcinoma | 8.5 | 1.9 | 38.6 |
| Other | 7.2 | 2.7 | 19.2 |
| Stage |  |  |  |
| Stage I (reference) | 1.0 | - | - |
| Stage II | 8.8 | 3.3 | 23.9 |
| Stage III | 51.8 | 13.7 | 195.1 |
| Smoking status |  |  |  |
| Never (reference) | 1.0 | - | - |
| Ex-smoker | 1.6 | 0.3 | 10.3 |
| Current smoker | 1.6 | 0.3 | 8.4 |

Multivariable logistic regression analysis for ctDNA detection pre-treatment (n=165). Explored variables include histology, gender, smoking status and stage category, with output presented as odds ratio and 95% confidence intervals. *CI = Confidence Interval.*
