# Supplementary material for: Recurrence prediction using circulating tumor DNA in patients with early-stage non-small cell lung cancer after treatment with curative intent: A retrospective validation study
Source: PLoS Med. 2025 Apr 15;22(4):e1004574. doi: 10.1371/journal.pmed.1004574 (PMC12021277; doi:10.1371/journal.pmed.1004574)
Supplement: S5 Table — Recurrence prediction by ctDNA detection in the landmark timeframe in the combined (N = 139), LEMA (N = 82) and LUCID (N = 57) cohorts. The landmark timeframe includes the first (positive) sample collected between 2 weeks and 4 months from the end of treatment. *A patient was regarded as ctDNA-positive if at least one sample in the specified time window was positive for ctDNA. Due to the small number, patients with stage 0 disease were grouped with patients with stage I disease. ΣRepresenting potential false positives. ςRepresenting potential false negatives. Sens, Sensitivity; Spec, Specificity; PPV, Positive Predictive Value; NPV, Negative Predictive Value; CI, Confidence Interval. (DOCX) [file pmed.1004574.s005.docx]

**S5 Table** Recurrence prediction and landmark ctDNA detection.

| **Detection of ctDNA within the landmark timeframe** | **ctDNA positive*** (*N*) | | | **ctDNA negative** (*N*) | | | **Sens** (%, *95% CI*) | **Spec** (%, *95% CI*) | **PPV** (%, *95% CI*) | **NPV** (%, *95% CI*) |
| --- | --- | --- | --- | --- | --- | --- | --- | --- | --- | --- |
|  | **Relapse** | | **No relapse^Σ^** | **No relapse** | | **Relapse^ς^** |  |  |  |  |
| **LEMA and LUCID combined** |  | | |  | | |  |  |  |  |
| All stages (*N*=139) | 17 | | 1 | 95 | | 26 | 39.5  *25.0,55.6* | 99.0  *94.3,100* | 94.4  *70.0,99.2* | 78.5  *74.1,82.3* |
| - Stage I (*N*=81) | 3 | | 0 | 66 | | 12 | 20.0  *4.3,48.1* | 100  *94.6,100* | 100  *29.2,100* | 84.6  *81.0,87.6* |
| - Stage II and III (*N*=58) | 14 | | 1 | 29 | | 14 | 50.0  *30.7,69.4* | 96.7  *82.8,99.9* | 93.3  *66.3,99.0* | 67.4  *58.7,75.1* |
| **LEMA cohort** |  | | |  | | |  |  |  |  |
| All stages (*N*=82) | 7 | 1 | | 58 | 16 | | 30.4  *13.2,52.9* | 98.3  *90.9,99.7* | 87.5  *47.7,98.2* | 78.4  *73.4,82.6* |
| - Stage I (*N*=47) | 1 | 0 | | 39 | 7 | | 12.5  *0.3,52.7* | 100  *91.0,100* | 100  *2.5,100* | 84.8  *81.1,87.9* |
| - Stage II and III (*N*=35) | 6 | 1 | | 19 | 9 | | 40.0  *16.3,67.7* | 95.0  *75.1,99.9* | 85.7  *44.6,97.8* | 67.9  *58.0,76.4* |
| **LUCID cohort** |  | | |  | | |  |  |  |  |
| All stages (*N*=57) | 10 | 0 | | 37 | 10 | | 50.0  *27.2,72.8* | 100  *90.5,100* | 100  *69.2,100* | 78.7  *70.5,85.2* |
| - Stage I (*N*=34) | 2 | 0 | | 10 | 5 | | 28.6  *3.7,71.0* | 100  *69.2,100* | 100  *15.8,100* | 66.7  *55.6,76.2* |
| - Stage II and III (*N*=23) | 8 | 0 | | 27 | 5 | | 61.5  *31.6,86.1* | 100  *87.2,100* | 100  *63.1,100* | 84.4  *73.1,91.5* |

Recurrence prediction by ctDNA detection in the landmark timeframe in the combined (*N*=139), LEMA (*N*=82) and LUCID (*N*=57) cohorts. The landmark timeframe includes the first (positive) sample collected between 2 weeks and 4 months from the end of treatment. * A patient was regarded as ctDNA-positive if at least one sample in the specified time window was positive for ctDNA. Due to the small number, stage 0 patient were grouped with stage I patients. **^Σ^** Representing potential false positives. **^ς^** Representing potential false negatives. *Sens = Sensitivity, Spec = Specificity, PPV = Positive Predictive Value, NPV = Negative Predictive Value, CI = Confidence Interval.*
