# Supplementary material for: Recurrence prediction using circulating tumor DNA in patients with early-stage non-small cell lung cancer after treatment with curative intent: A retrospective validation study
Source: PLoS Med. 2025 Apr 15;22(4):e1004574. doi: 10.1371/journal.pmed.1004574 (PMC12021277; doi:10.1371/journal.pmed.1004574)
Supplement: S6 Table — Recurrence prediction by ctDNA detection in serial samples collected ≥14 days post-treatment, in the combined cohort (N = 193), LEMA (N = 116) and LUCID (N = 77) cohort. *A patient was regarded as ctDNA-positive if at least one sample in the specified time window was positive for ctDNA. Due to the small number, patients with stage 0 disease were grouped with patients with stage I disease. ΣRepresenting potential false positives. ςRepresenting potential false negatives. Sens, Sensitivity; Spec, Specificity; PPV, Positive Predictive Value; NPV, Negative Predictive Value; CI, Confidence Interval. (DOCX) [file pmed.1004574.s006.docx]

**S6 Table** Recurrence prediction and longitudinal ctDNA detection (≥14 days post-treatment)

| **Detection of ctDNA ≥14 days post treatment** | **ctDNA positive*** (*N*) | | **ctDNA negative** (*N*) | | **Sens** (%, *95% CI*) | **Spec** (%, *95% CI*) | **PPV** (%, *95% CI*) | **NPV** (%, *95% CI*) |
| --- | --- | --- | --- | --- | --- | --- | --- | --- |
|  | **Relapse** | **No relapse^Σ^** | **No Relapse** | **Relapse^ς^** |  |  |  |  |
| **LEMA and LUCID combined** |  | |  | |  |  |  |  |
| All stages (*N*=193) | 41 | 4 | 123 | 25 | 62.1  *49.3,73.8* | 96.9  *92.1,99.1* | 91.1  *79.3,96.5* | 83.1  *78.3,87.0* |
| - Stage I (*N*=102) | 10 | 1 | 82 | 9 | 52.6  *28.9,75.6* | 98.8  *93.5,100* | 90.9  *57.7,98.7* | 90.1  *85.0,93.6* |
| - Stage II and III (*N*=91) | 31 | 3 | 41 | 16 | 66.0  *50.7,79.1* | 93.2  *81.3,98.6* | 91.2  *77.3,96.9* | 71.9  *63.1,79.4* |
| **LEMA cohort** |  | |  | |  |  |  |  |
| All stages (*N*=116) | 23 | 2 | 76 | 15 | 60.5  *43.4,76.0* | 97.4  *91.0,99.7* | 92.0  *74.1,97.9* | 83.5  *77.3,88.3* |
| - Stage I (*N*=62) | 6 | 0 | 50 | 6 | 50.0  *21.1,78.9* | 100  *92.9,100* | 100  *54.1,100* | 89.3  *82.6,93.6* |
| - Stage II and III (*N*=54) | 17 | 2 | 26 | 9 | 65.4  *44.3,82.8* | 92.9  *76.5,99.1* | 89.5  *68.5,97.1* | 74.3  *62.8,83.2* |
| **LUCID cohort** |  | |  | |  |  |  |  |
| All stages (*N*=77) | 18 | 2 | 47 | 10 | 64.3  *44.1,81.4* | 95.9  *86.0,99.5* | 90.0  *69.3,97.3* | 82.5  *74.0,88.6* |
| - Stage I (*N*=40) | 4 | 1 | 32 | 3 | 57.1  *18.4,90.1* | 97.0  *84.2,99.9* | 80.0  *34.4,96.8* | 91.4  *81.9,96.2* |
| - Stage II and III (*N*=37) | 14 | 1 | 15 | 7 | 66.7  *43.0,85.4* | 93.8  *69.8,99.8* | 93.3  *67.2,99.0* | 68.2  *53.6,79.9* |

Recurrence prediction by ctDNA detection in serial samples collected ≥14 days post-treatment, in the combined cohort (*N*=193), LEMA (*N*=116) and LUCID (*N*=77) cohort. * A patient was regarded as ctDNA-positive if at least one sample in the specified time window was positive for ctDNA. Due to the small number, stage 0 patient were grouped with stage I patients. **^Σ^** Representing potential false positives. **^ς^** Representing potential false negatives. *Sens = Sensitivity, Spec = Specificity, PPV = Positive Predictive Value, NPV = Negative Predictive Value, CI = Confidence Interval.*
