# Supplementary material for: Recurrence prediction using circulating tumor DNA in patients with early-stage non-small cell lung cancer after treatment with curative intent: A retrospective validation study
Source: PLoS Med. 2025 Apr 15;22(4):e1004574. doi: 10.1371/journal.pmed.1004574 (PMC12021277; doi:10.1371/journal.pmed.1004574)
Supplement: S7 Table — Recurrence prediction by ctDNA detection post-treatment when including only samples obtained 1–3 days after the end date of the curative intended treatment in the combined (N = 80), LEMA (N = 32) and LUCID (N = 48) cohort. *A patient was regarded as ctDNA-positive if at least one sample in the specified time window was positive for ctDNA. Due to the small number, patients with stage 0 disease were grouped with patients with stage I disease. ΣRepresenting potential false positives. ςRepresenting potential false negatives. Sens, Sensitivity; Spec, Specificity; PPV, Positive Predictive Value; NPV, Negative Predictive Value; CI, Confidence Interval. (DOCX) [file pmed.1004574.s007.docx]

**S7 Table** Recurrence prediction and ctDNA detection 1-3 days after curative treatment.

| **Detection of ctDNA within 1-3 days post-treatment** | **ctDNA positive*** (*N*) | | | **ctDNA negative** (*N*) | | **Sens** (%, *95% CI*) | **Spec** (%, *95% CI*) | **PPV** (%, *95% CI*) | **NPV** (%, *95% CI*) |
| --- | --- | --- | --- | --- | --- | --- | --- | --- | --- |
|  | **Relapse** | | **No relapse^Σ^** | **No relapse** | **Relapse^ς^** |  |  |  |  |
| **LEMA and LUCID combined** | |  | | | |  |  |  |  |
| All stages (*N*=80) | 8 | | 7 | 55 | 10 | 44.4  *21.5,69.2* | 88.7  *78.1,95.3* | 53.3  *32.4,73.1* | 84.6  *78.3,89.4* |
| - Stage I (*N*=49) | 3 | | 2 | 40 | 4 | 42.9  *9.9,81.6* | 95.2  *83.8,99.4* | 60.0  *23.2,88.1* | 90.9  *84.0,95.0* |
| - Stage II and III (*N*=31) | 5 | | 5 | 15 | 6 | 45.5  *16.8,76.6* | 75.0  *50.9,91.3* | 50.0  *26.9,73.1* | 71.4  *57.9,81.9* |
| **LEMA cohort** | |  | | | |  |  |  |  |
| All stages (*N*=32) | 2 | | 1 | 24 | 5 | 28.6  *3.7,71.0* | 96.0  *76.7,99.9* | 66.7  *17.4,95.0* | 82.8  *74.9,88.5* |
| - Stage I (*N*=19) | 1 | | 0 | 17 | 1 | 50.0  *1.3,98.7* | 100  *80.5,100* | 100  *2.5,100* | 94.4  *81.0,98.6* |
| - Stage II and III (*N*=13) | 1 | | 1 | 7 | 4 | 20.0  *0.5,71.6* | 87.5  *47.4,99.7* | 50.0  *7.3,92.7* | 63.6  *51.2,74.5* |
| **LUCID cohort** |  | | |  | |  |  |  |  |
| All stages (*N*=48) | 6 | | 6 | 31 | 5 | 54.5  *23.4,83.3* | 83.8  *68.0,93.8* | 50.0  *28.7,71.3* | 86.1  *76.2,92.3* |
| - Stage I (*N*=30) | 2 | | 2 | 23 | 3 | 40.0  *5.3,85.3* | 92.0  *74.0,99.0* | 50.0  *15.3,84.7* | 88.5  *78.8,94.1* |
| - Stage II and III (*N*=18) | 4 | | 4 | 8 | 2 | 66.7  *22.3,95.7* | 66.7  *34.9,90.1* | 50.0  *27.3,72.7* | 80.0  *54.6,93.0* |

Recurrence prediction by ctDNA detection post-treatment when including only samples obtained 1-3 days after the end date of the curative intended treatment in the combined (*N*=80), LEMA (*N*=32) and LUCID (*N*=48) cohort. * A patient was regarded as ctDNA-positive if at least one sample in the specified time window was positive for ctDNA. Due to the small number, stage 0 patient were grouped with stage I patients. **^Σ^** Representing potential false positives. **^ς^** Representing potential false negatives. *Sens = Sensitivity, Spec = Specificity, PPV = Positive Predictive Value, NPV = Negative Predictive Value, CI = Confidence Interval.*
