# Supplementary material for: Recurrence prediction using circulating tumor DNA in patients with early-stage non-small cell lung cancer after treatment with curative intent: A retrospective validation study
Source: PLoS Med. 2025 Apr 15;22(4):e1004574. doi: 10.1371/journal.pmed.1004574 (PMC12021277; doi:10.1371/journal.pmed.1004574)
Supplement: S8 Table — Recurrence prediction by ctDNA detection ≥14 days post-treatment in patients that were ctDNA positive pre-treatment. *A patient was regarded as ctDNA-positive if at least one sample in the specified time window was positive for ctDNA. In the landmark timeframe, only one sample per patient, the first (positive) sample within 2 weeks to 4 months after the treatment end date, was considered. ΣRepresenting potential false positives. ςRepresenting potential false negatives. Sens, Sensitivity; Spec, Specificity; PPV, Positive Predictive Value; NPV, Negative Predictive Value; CI, Confidence Interval. (DOCX) [file pmed.1004574.s008.docx]

**S8 Table** Recurrence prediction and ctDNA detection ≥14 days post-treatment in pre-treatment positive patients.

| **LEMA and LUCID combined** | **ctDNA positive*** (*N*) | | **ctDNA negative** (*N*) | | **Sens** (%, *95% CI*) | **Spec** (%, *95% CI*) | **PPV** (%, *95% CI*) | **NPV** (%, *95% CI*) |
| --- | --- | --- | --- | --- | --- | --- | --- | --- |
|  | **Relapse** | **No relapse^Σ^** | **No relapse** | **Relapse^ς^** |  |  |  |  |
| Positive at ≥14 days and pre-treatment (*N*=69) | 26 | 2 | 36 | 5 | 83.9  *66.3,94.6* | 94.7  *82.3,99.4* | 92.9  *77.0,98.1* | 87.8  *76.3,94.2* |
| Positive at landmark and pre-treatment (*N*=47) | 11 | 1 | 26 | 9 | 55.0  *31.5,76.9* | 96.3  *81.0,99.9* | 91.7  *60.7,98.7* | 74.3  *63.9,82.5* |

Recurrence prediction by ctDNA detection ≥14 days post-treatment in patients that were ctDNA positive pre-treatment. * A patient was regarded as ctDNA-positive if at least one sample in the specified time window was positive for ctDNA. In the landmark timeframe, only one sample per patient, the first (positive) sample within 2 weeks to 4 months after the treatment end date, was considered. **^Σ^** Representing potential false positives. **^ς^** Representing potential false negatives. *Sens = Sensitivity, Spec = Specificity, PPV = Positive Predictive Value, NPV = Negative Predictive Value, CI = Confidence Interval.*
