# Supplementary material for: Recurrence prediction using circulating tumor DNA in patients with early-stage non-small cell lung cancer after treatment with curative intent: A retrospective validation study
Source: PLoS Med. 2025 Apr 15;22(4):e1004574. doi: 10.1371/journal.pmed.1004574 (PMC12021277; doi:10.1371/journal.pmed.1004574)
Supplement: S9 Table — Concordance Probability Estimate (CPE) analysis to determine the relative contribution of ctDNA status and disease stage to the recurrence prediction ability at different time points. OS, Overall Survival; RFS, Recurrence Free Survival. (DOCX) [file pmed.1004574.s009.docx]

**S9 Table** Exploration of relative contribution of ctDNA status and disease stage to the recurrence prediction

|  | **Model including all variables** |  | **Model minus stage** |  | **Model minus ctDNA** |  | **Model minus ctDNA & stage** |  |
| --- | --- | --- | --- | --- | --- | --- | --- | --- |
| **Analysis** | **CPE** | **se.CPE** | **CPE** | **se.CPE** | **CPE** | **se.CPE** | **CPE** | **se.CPE** |
| OS, ctDNA detection at baseline | 0.72 | 0.03 | 0.69 | 0.03 | 0.72 | 0.03 | 0.68 | 0.04 |
| RFS, ctDNA detection at baseline | 0.70 | 0.03 | 0.68 | 0.03 | 0.69 | 0.03 | 0.65 | 0.03 |
| OS, ctDNA detection at landmark | 0.72 | 0.03 | 0.70 | 0.03 | 0.70 | 0.03 | 0.61 | 0.04 |
| RFS, ctDNA detection at landmark | 0.71 | 0.03 | 0.70 | 0.03 | 0.67 | 0.03 | 0.61 | 0.04 |
| OS, ctDNA detection at FU | 0.74 | 0.02 | 0.73 | 0.02 | 0.73 | 0.03 | 0.67 | 0.03 |
| RFS, ctDNA detection at FU | 0.75 | 0.02 | 0.74 | 0.02 | 0.71 | 0.02 | 0.63 | 0.03 |

Concordance Probability Estimate (CPE) analysis to determine the relative contribution of ctDNA status and disease stage to the recurrence prediction ability at different timepoints. *OS = Overall Survival, RFS = Recurrence Free Survival, FU = Follow-up.*
