# Supplementary material for: Recurrence prediction using circulating tumor DNA in patients with early-stage non-small cell lung cancer after treatment with curative intent: A retrospective validation study
Source: PLoS Med. 2025 Apr 15;22(4):e1004574. doi: 10.1371/journal.pmed.1004574 (PMC12021277; doi:10.1371/journal.pmed.1004574)
Supplement: S10 Table — Assessment of effect of eVAF at baseline in ctDNA positive samples, and landmark on survival outcomes. OS, Overall Survival; RFS, Recurrence Free Survival. (DOCX) [file pmed.1004574.s010.docx]

**S10 Table** Baseline and landmark ctDNA eVAF, and survival outcomes.

|  | Hazard Ratio | 95% Confidence Interval | p-value |
| --- | --- | --- | --- |
| RaDaR eVAF at baseline  OS | 1.02 | 0.85,1.24 | 0.8 |
| RaDaR eVAF at baseline  RFS | 1.07 | 0.93,1.24 | 0.4 |
| RaDaR eVAF at landmark  OS | 1.05 | 0.98,1.12 | 0.15 |
| RaDaR eVAF at landmark  RFS | 1.03 | 0.97,1.10 | 0.3 |

Assessment of effect of eVAF at baseline in ctDNA positive samples, and landmark on survival outcomes. *OS = Overall Survival, RFS = Recurrence Free Survival*.
