# Supplementary material for: Recurrence prediction using circulating tumor DNA in patients with early-stage non-small cell lung cancer after treatment with curative intent: A retrospective validation study
Source: PLoS Med. 2025 Apr 15;22(4):e1004574. doi: 10.1371/journal.pmed.1004574 (PMC12021277; doi:10.1371/journal.pmed.1004574)
Supplement: S11 Table — Exploration of the effect of exclusion (i.e., treating end of curative treatment as T = 0) or inclusion (i.e., treating end of adjuvant treatment as T = 0) of adjuvant treatment in the analysis. *In the landmark timeframe, only one sample per patient, the first (positive) within 14–122 days after the treatment end date, was considered. In the serial analyses, a patient was regarded as ctDNA-positive if at least one sample ≥14 days after end of treatment was ctDNA positive. ΣRepresenting potential false positives. ς Representing potential false negatives. Sens, Sensitivity; Spec, Specificity; PPV, Positive Predictive Value; NPV, Negative Predictive Value; CI, Confidence Interval. (DOCX) [file pmed.1004574.s011.docx]

**S11 Table** Exploration of the effect of adjuvant treatment on survival analysis.

| **LEMA and LUCID combined** | **ctDNA positive*** (*N*) | | **ctDNA negative** (*N*) | | **Sens** (%, *95% CI*) | **Spec** (%, *95% CI*) | **PPV** (%, *95% CI*) | **NPV** (%, *95% CI*) |
| --- | --- | --- | --- | --- | --- | --- | --- | --- |
|  | **Relapse** | **No relapse**^Σ^ | **No relapse** | **Relapse**^ς^ |  |  |  |  |
| **Landmark* (≥14 – 122 days)** |  | |  | |  |  |  |  |
| Excluding adjuvant treatment (*N*=139) | 17 | 1 | 95 | 26 | 39.5  *25.0,55.6* | 99.0  *94.3,100* | 94.4  *70.0,99.2* | 78.5  *74.1,82.3* |
| Including adjuvant treatment (*N*=134) | 16 | 0 | 90 | 28 | 36.4  *22.4,52.2* | 100  *96.0,100* | 100  *79.4,100* | 76.3  *72.0,80.1* |
| **Serial samples* (≥14 days)** |  | |  | |  |  |  |  |
| Including adjuvant treatment (*N*=193) | 41 | 4 | 123 | 25 | 62.1  *49.3,73.8* | 96.9  *92.1,99.1* | 91.1  *79.3,94.5* | 83.1  *78.3,87.0* |
| Excluding adjuvant treatment (*N*=178) | 39 | 2 | 112 | 25 | 60.9  *47.9,72.9* | 98.2  *93.8,99.8* | 95.1  *83.0,98.7* | 81.8  *76.7,85.9* |

Exploration of the effect of exclusion (i.e. treating end of curative treatment as T=0) or inclusion (i.e. treating end of adjuvant treatment as T=0) of adjuvant treatment in the analysis. * In the landmark timeframe, only one sample per patient, the first (positive) within 14 to 122 days after the treatment end date, was considered. In the serial analyses, a patient was regarded as ctDNA-positive if at least one sample ≥14 days after end of treatment was ctDNA positive. Σ Representing potential false positives. ς Representing potential false negatives. *Sens = Sensitivity, Spec = Specificity, PPV = Positive Predictive Value, NPV = Negative Predictive Value, CI = Confidence Interval.*
