# Supplementary material for: Recurrence prediction using circulating tumor DNA in patients with early-stage non-small cell lung cancer after treatment with curative intent: A retrospective validation study
Source: PLoS Med. 2025 Apr 15;22(4):e1004574. doi: 10.1371/journal.pmed.1004574 (PMC12021277; doi:10.1371/journal.pmed.1004574)
Supplement: S12 Table — Analysis of the time interval between sample collection and disease recurrence. Shown are the number of events to occur within a specific time frame relative to recurrence; 1, at any time relative to recurrence; 2, within 6 months prior to recurrence; 3, within 3 months prior to recurrence. (DOCX) [file pmed.1004574.s012.docx]

**S12 Table** Analysis of time interval between samples and recurrence

| **LEMA and LUCID combined** | **Relapse*** (*N*) | | **Sensitivity (%)** |
| --- | --- | --- | --- |
|  | **ctDNA +** | **ctDNA -** |  |
| All follow-up samples (*N*=193) | 41 | 25 | 62.1 |
| Only samples ≤6 months of recurrence (*N*=26) | 17 | 9 | 65.4 |
| Only samples ≤3 months of recurrence (*N=16)* | 10 | 6 | 62.5 |

Analysis of the time interval between sample collection and disease recurrence. Shown are the number of events to occ**ur** within a specific time frame relative to recurrence; 1, at any time relative to recurrence; 2, within 6 months prior to recurrence; 3, within 3 months prior to recurrence.
