# Supplementary material for: Recurrence prediction using circulating tumor DNA in patients with early-stage non-small cell lung cancer after treatment with curative intent: A retrospective validation study
Source: PLoS Med. 2025 Apr 15;22(4):e1004574. doi: 10.1371/journal.pmed.1004574 (PMC12021277; doi:10.1371/journal.pmed.1004574)
Supplement: S13 Table — Characteristics of patients with and without recurrence, categorized by ctDNA prediction of recurrence ≥14 days post-treatment. Statistical analyses was performed using the Pearson Chi-Squared Test, or when appropriate the Fisher–Freeman–Halton Exact Test, for categorical variables and the independent samples T test for nominal variables. Statistical analyses were not performed for patients without recurrence because of a sample size ≤5 patients. We acknowledge that numbers for these analyses are small and therefore these findings should be interpreted with caution. *Within both LEMA and LUCID cohorts, on occasion mixed histological subtypes were observed within the same tumor (e.g., an adenocarcinoma with both lepidic and acinar components) (N = 7). For LEMA, the relative composition of these tumors was known, allowing for determination of the predominant subtype and inclusion of these cases based on that subtype. For the LUCID cohort, this data was not available and, as such, the cases with mixed histological subtypes were excluded from this analysis. ^AIS, adenocarcinoma in situ; MIA, minimally invasive adenocarcinoma. +Additional information about the location of metastasis was available only for the LEMA cohort. &Other locations of metastases include kidney, liver or multiple locations. (DOCX) [file pmed.1004574.s013.docx]

**S13 Table** Exploration of clinical and biological characteristics in patients that did or did not recur, and ctDNA detection ≥14 days post-treatment.

| **LEMA and LUCID combined** | **Patients with recurrence** | | | **Patients without recurrence** | |
| --- | --- | --- | --- | --- | --- |
|  | **ctDNA positive**  **(true positives)** | **ctDNA negative (false negatives)** |  | **ctDNA negative**  **(true negatives)** | **ctDNA positive  (false positives)** |
| Stage, *N*=193 |  |  |  |  |  |
| I | 10 (24%) | 9 (36%) |  | 82 (67%) | 1 (25%) |
| II | 7 (17%) | 7 (28%) | p=0.225 | 24 (19%) | 1 (25%) |
| III | 24 (59%) | 9 (36%) |  | 17 (14%) | 2 (50%) |
| Histology, *N*=193 |  |  |  |  |  |
| Adenocarcinoma | 26 (63%) | 22 (88%) |  | 82 (67%) | 2 (50%) |
| Squamous cell carcinoma | 11 (27%) | 3 (12%) | p=0.081 | 31 (25%) | 2 (50%) |
| Other | 4 (10%) | 0 |  | 10 (8%) | 0 |
| Histologic subtype*,  *N*=109 |  |  |  |  |  |
| AIS/MIA^ | 0 | 0 |  | 8 (11%) | 0 |
| Lepidic | 1 (5%) | 5 (42%) |  | 19 (26%) | 0 |
| Papillar | 0 | 1 (8%) |  | 3 (4%) | 0 |
| Acinar | 12 (57%) | 5 (42%) |  | 29 (39%) | 2 (100%) |
| Micropapillar | 3 (14%) | 0 | p=0.024 | 2 (2%) | 0 |
| Solid | 5 (24%) | 1 (8%) |  | 13 (18%) | 0 |
| PL status, *N*=171 |  |  |  |  |  |
| PL0 | 19 (59%) | 11 (61%) |  | 91 (77%) | 1 (33%) |
| PL1 | 5 (16%) | 6 (33%) | p=0.156 | 19 (16%) | 2 (67%) |
| PL2-3 | 8 (25%) | 1 (6%) |  | 8 (7%) | 0 |
| Pathologic T status,  *N*=163 |  |  |  |  |  |
| T1 | 7 (23%) | 1 (7%) |  | 62 (54%) | 1 (33%) |
| T2 | 10 (32%) | 10 (67%) |  | 31 (27%) | 2 (67%) |
| T3 | 6 (19%) | 4 (27%) | p=0.033 | 14 (12%) | 0 |
| T4 | 8 (26%) | 0 |  | 7 (6%) | 0 |
| Pathologic N status,  *N*=164 |  |  |  |  |  |
| N0 | 15 (48%) | 10 (67%) |  | 93 (80%) | 1 (33%) |
| N1 | 8 (26%) | 2 (13%) | p=0.588 | 14 (11%) | 1 (33%) |
| N2 | 8 (26%) | 3 (20%) |  | 9 (8%) | 1 (33%) |
| Median tumour diameter in mm (IQR), *N* =174 |  |  |  |  |  |
| Stage I | 21 (21 – 37) | 21 (15 – 25) | P=0.601 | 21 (14 – 29.5) | - |
| Stage II | 42 (35 – 48) | 40 (37-60) | p=0.740 | 42 (31 – 60) | - |
| Stage III | 52 (365 – 89.5) | 37.5 (22 – 44) | P=0.116 | 61 (31 – 98.5) | 41 (37 – 45) |
| Location metastasis**^+^**,  *N*=36 |  |  |  |  |  |
| Lung | 8 (36%) | 7 (50%) |  | - | - |
| Bone | 7 (32%) | 0 |  | - | - |
| Brain | 2 (9%) | 4 (29%) | p=0.071 | - | - |
| Other^&^ | 5 (23%) | 3 (21%) |  | - | - |
| Tumour differentiation grade, *N*=165 |  |  |  |  |  |
| Grade I-II | 14 (39%) | 7 (39%) | p=1.000 | 25 (23%) | 2 (67%)) |
| Grade III-IV | 22 (61%) | 11 (61%) |  | 83 (77%) | 1 (33%) |

Characteristics of patients with and without recurrence, categorized by ctDNA prediction of recurrence ≥14 days post-treatment. Statistical analyses was performed using the Pearson Chi-Square Test, or when appropriate the Fisher-Freeman-Halton Exact Test, for categorical variables and the independent samples T-test for nominal variables. Statistical analyses were not performed for patients without recurrence because of a sample size ≤ 5 patients. We acknowledge that numbers for these analyses are small and therefore these findings should be interpreted with caution. * Within both LEMA and LUCID cohorts, on occasion mixed histological subtypes were observed within the same tumour (e.g. an adenocarcinoma with both lepidic and acinar components) (N=7). For LEMA, the relative composition of these tumours was known, allowing for determination of the predominant subtype and inclusion of these cases based on that subtype. For the LUCID cohort, this data was not available and, as such, the cases with mixed histological subtypes were excluded from this analysis. ^ *AIS = adenocarcinoma in situ, MIA = minimally invasive adenocarcinoma*. ^+^ Additional information about the location of metastasis was available only for the LEMA cohort. ^&^ Other locations of metastases include kidney, liver or multiple locations.
