# Supplementary material for: Recurrence prediction using circulating tumor DNA in patients with early-stage non-small cell lung cancer after treatment with curative intent: A retrospective validation study
Source: PLoS Med. 2025 Apr 15;22(4):e1004574. doi: 10.1371/journal.pmed.1004574 (PMC12021277; doi:10.1371/journal.pmed.1004574)
Supplement: S1 Fig — (A) Study design. (B) Flow diagram depicting the selection of the MRD patient cohort. (C) Flow diagram depicting the LEMA patient cohort, sample availability, and results of ctDNA analysis, categorized by clinical outcome. (PDF) [file pmed.1004574.s015.pdf]

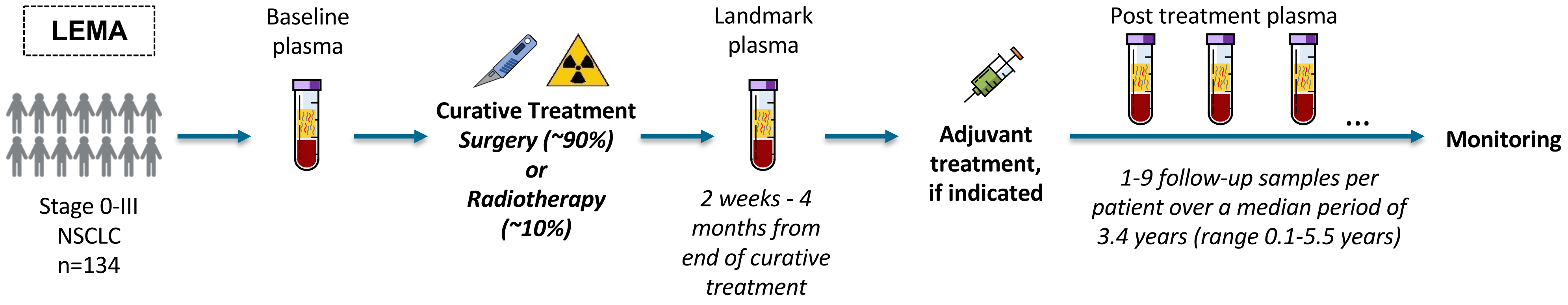

S1 Fig (A) Study design

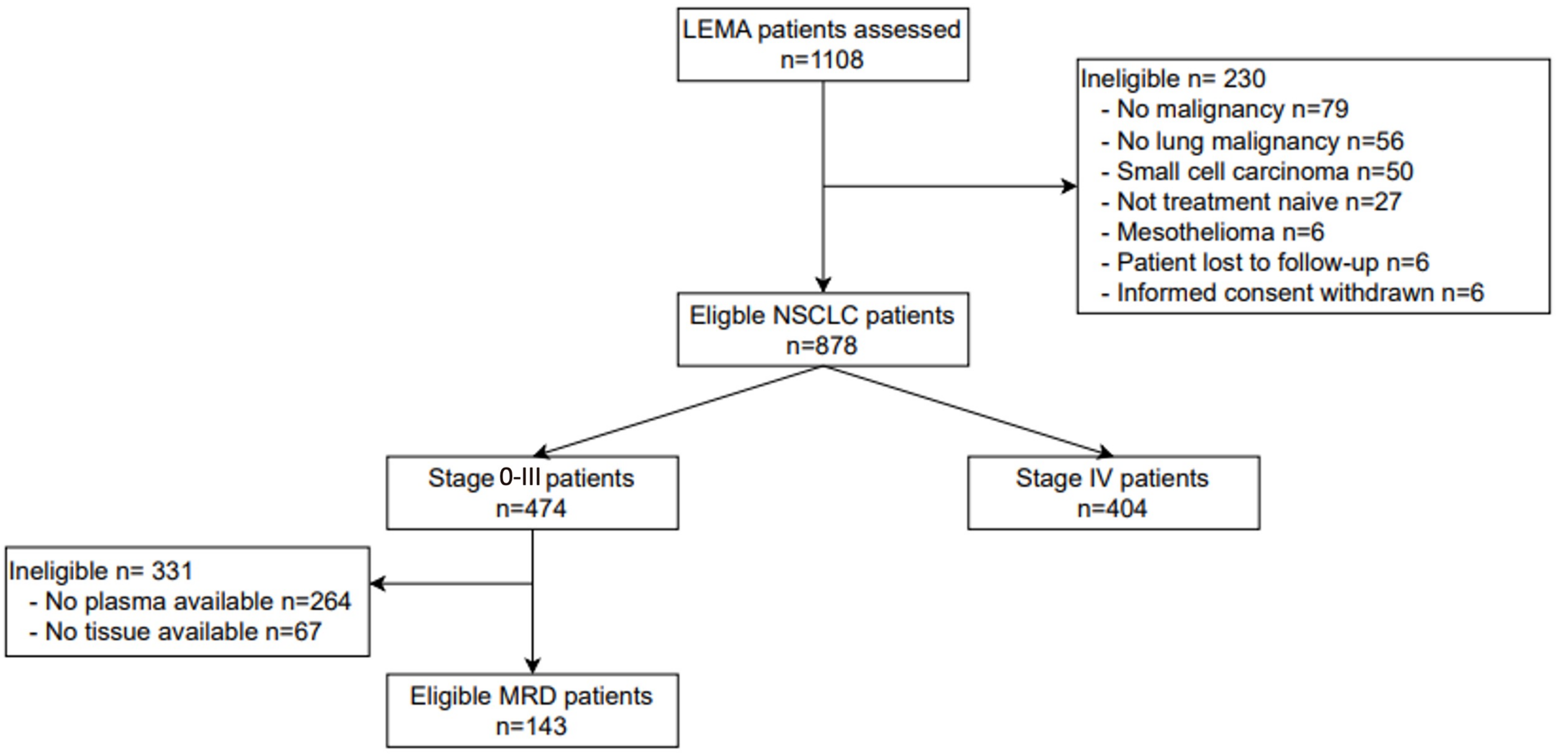

S1 Fig (B) Flow diagram depicting the selection of the MRD patient cohort

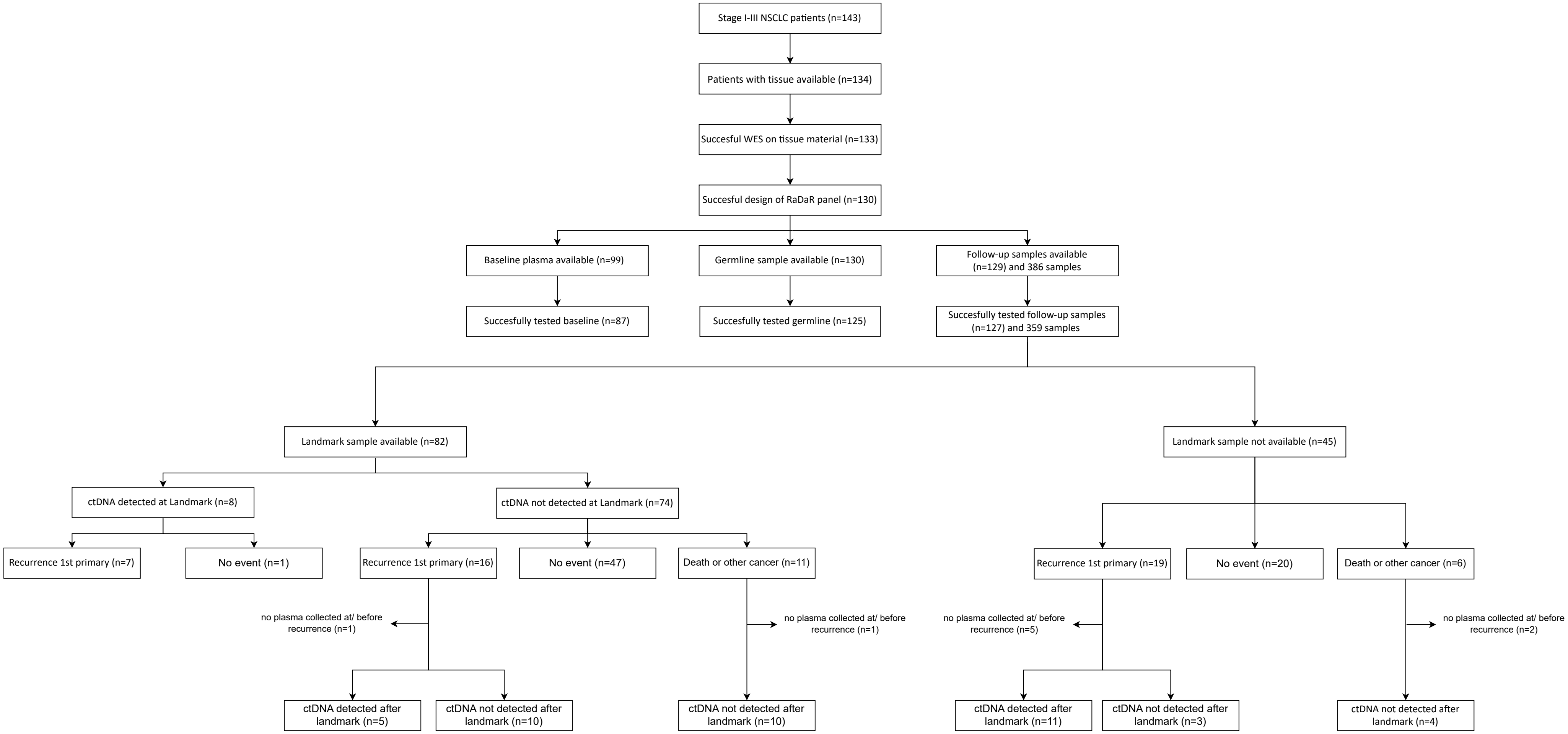

S1 Fig (C) Flow diagram depicting the LEMA patient cohort, sample availability and results of ctDNA analysis, categorised by clinical outcome
