# Supplementary material for: Recurrence prediction using circulating tumor DNA in patients with early-stage non-small cell lung cancer after treatment with curative intent: A retrospective validation study
Source: PLoS Med. 2025 Apr 15;22(4):e1004574. doi: 10.1371/journal.pmed.1004574 (PMC12021277; doi:10.1371/journal.pmed.1004574)
Supplement: S2 Fig — Longitudinal plasma monitoring in (A) stage 0 and I, (B) stage II, and (C) stage III LEMA patients. (D) Plasma monitoring in LEMA and LUCID, split by stage. Figures indicate when ctDNA was detected (red points) or not detected (white points). Clinical recurrence is indicated with an orange triangle. Time is measured from end of curative treatment (day 0) until end of follow-up (gray diamond). Treatment periods and type are indicated by coloured highlights. (PDF) [file pmed.1004574.s016.pdf]

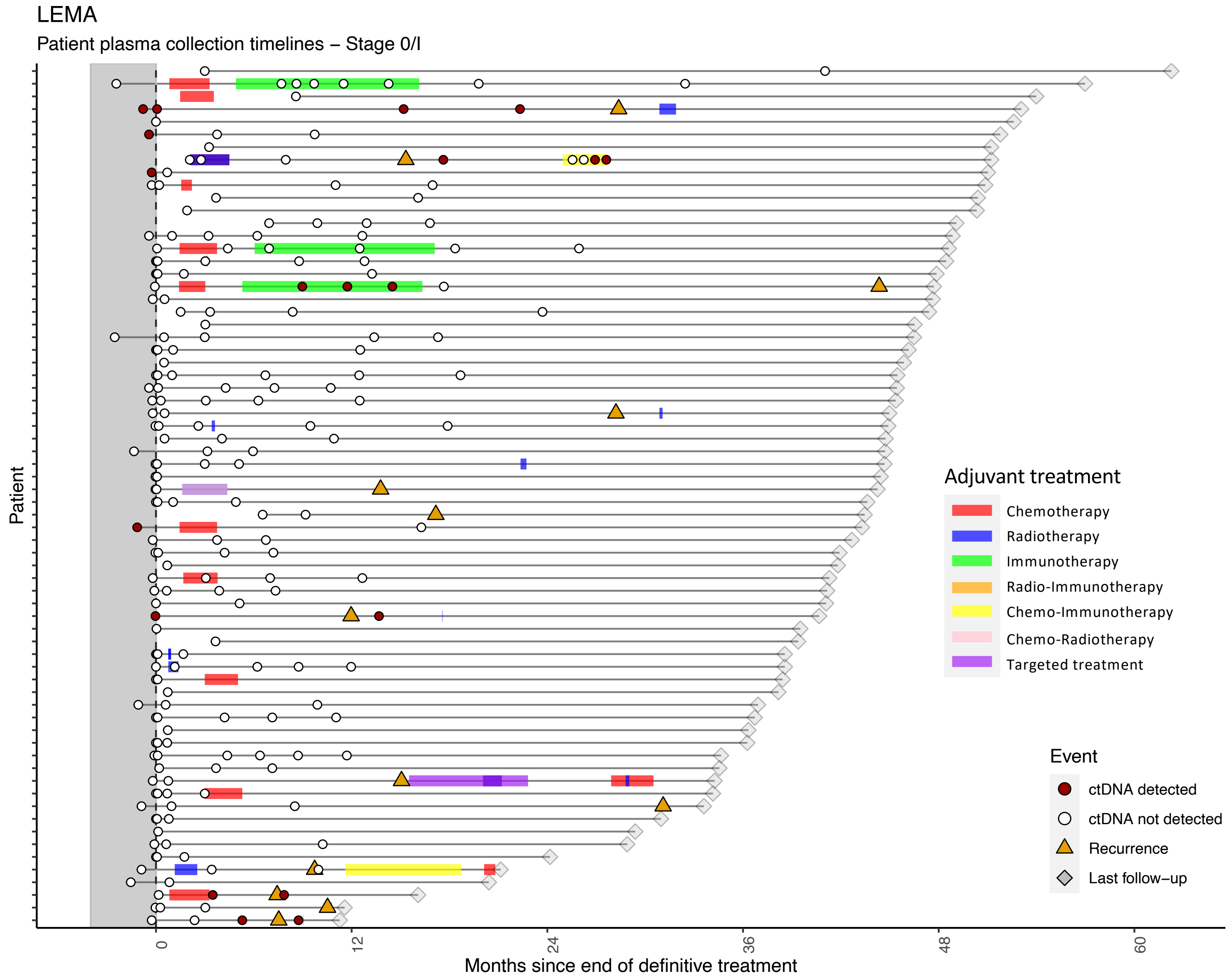

S2 Fig (A) Longitudinal plasma monitoring in stage 0 and I LEMA patients. Indicates when ctDNA was detected (red dots) or not detected (white dots). Clinical recurrence is indicated with an orange triangle. Time is measured from end of curative treatment (day 0) until end of follow-up (grey diamond). Treatment periods and type are indicated by coloured highlights.

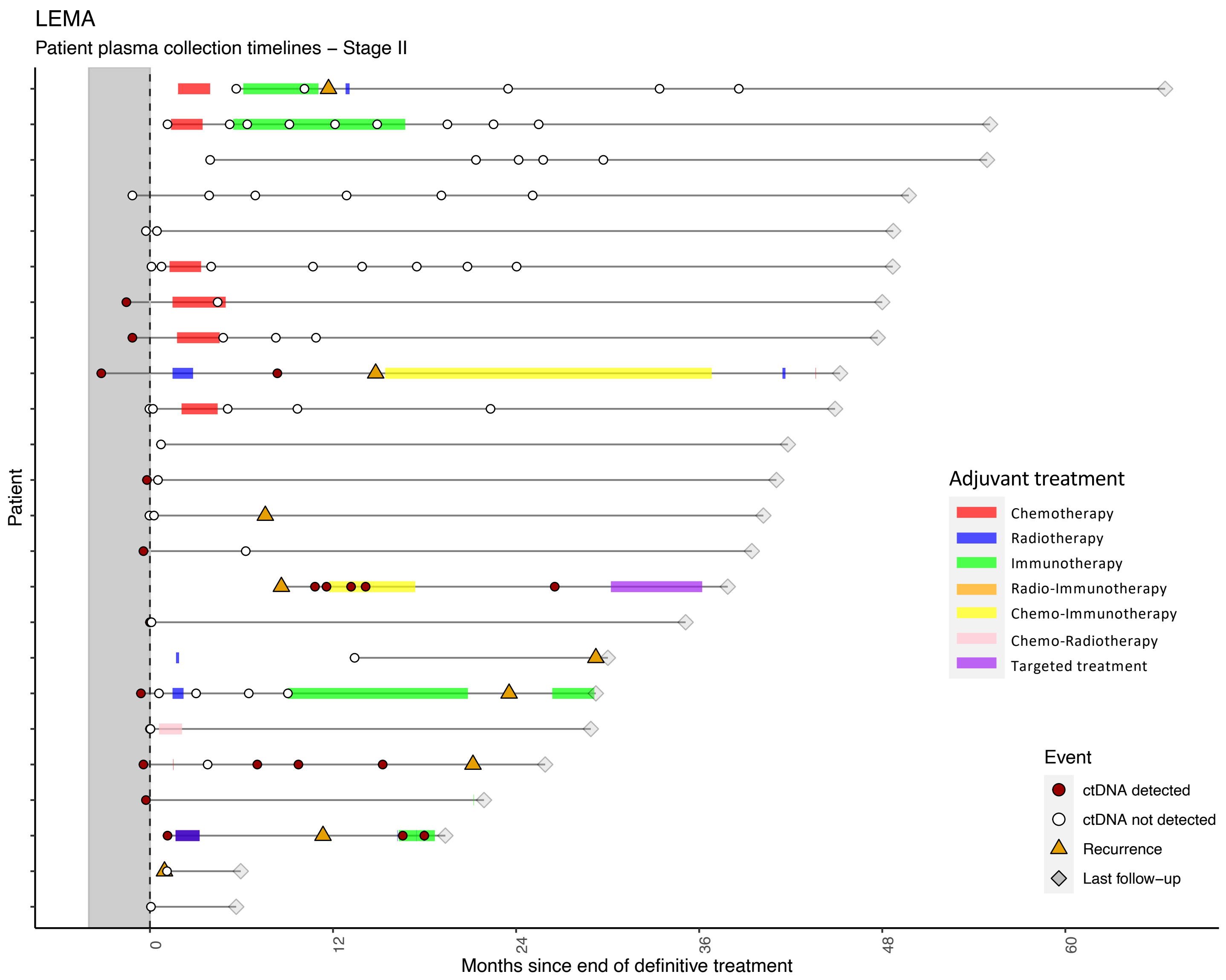

S2 Fig (B) Longitudinal plasma monitoring in stage II LEMA patients. Indicates when ctDNA was detected (red dots) or not detected (white dots). Clinical recurrence is indicated with an orange triangle. Time is measured from end of curative treatment (day 0) until end of follow-up (grey diamond). Treatment periods and type are indicated by coloured highlights.

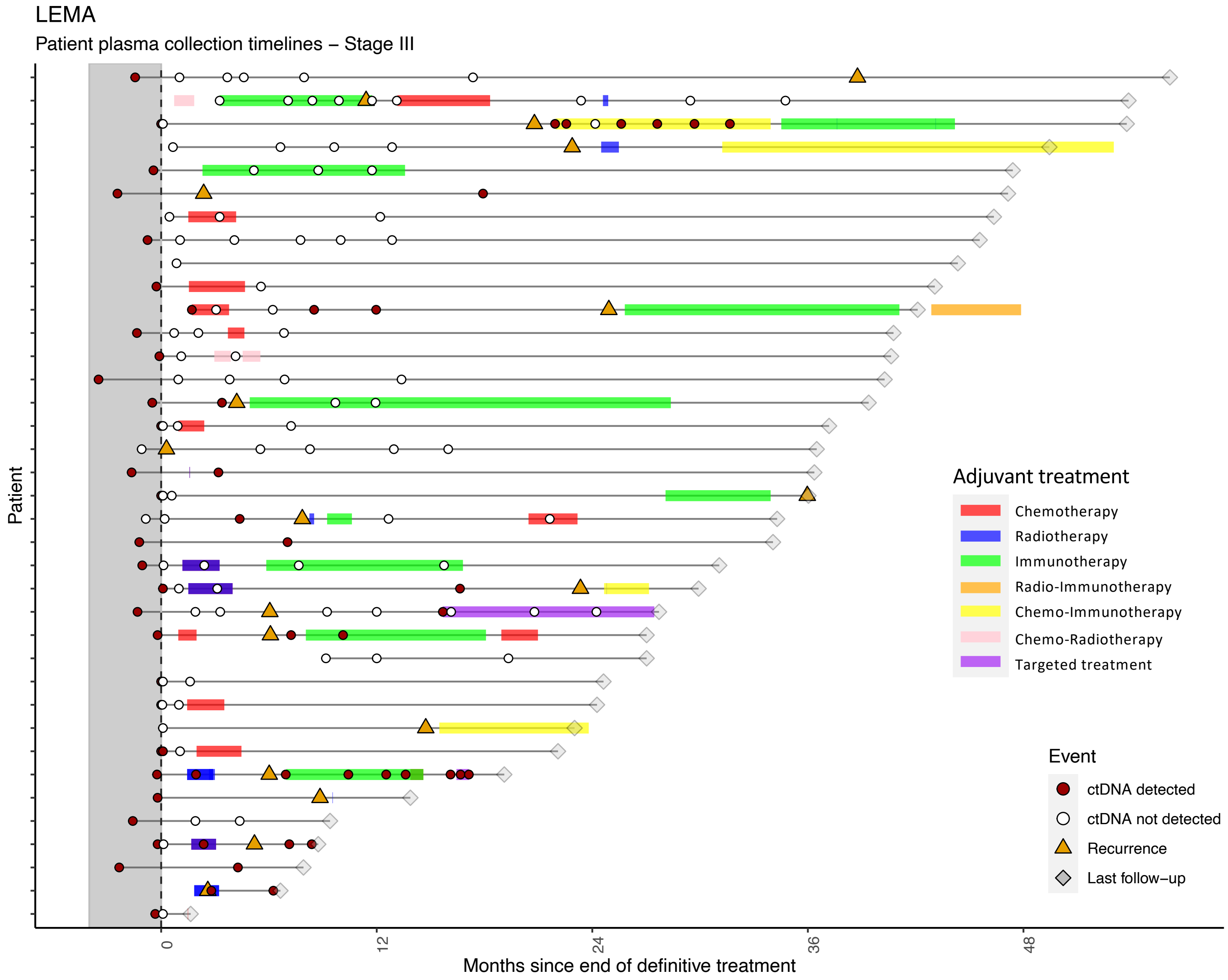

S2 Fig (C) Longitudinal plasma monitoring in stage III LEMA patients. Indicates when ctDNA was detected (red dots) or not detected (white dots). Clinical recurrence is indicated with an orange triangle. Time is measured from end of curative treatment (day 0) until end of follow-up (grey diamond). Treatment periods and type are indicated by coloured highlights.

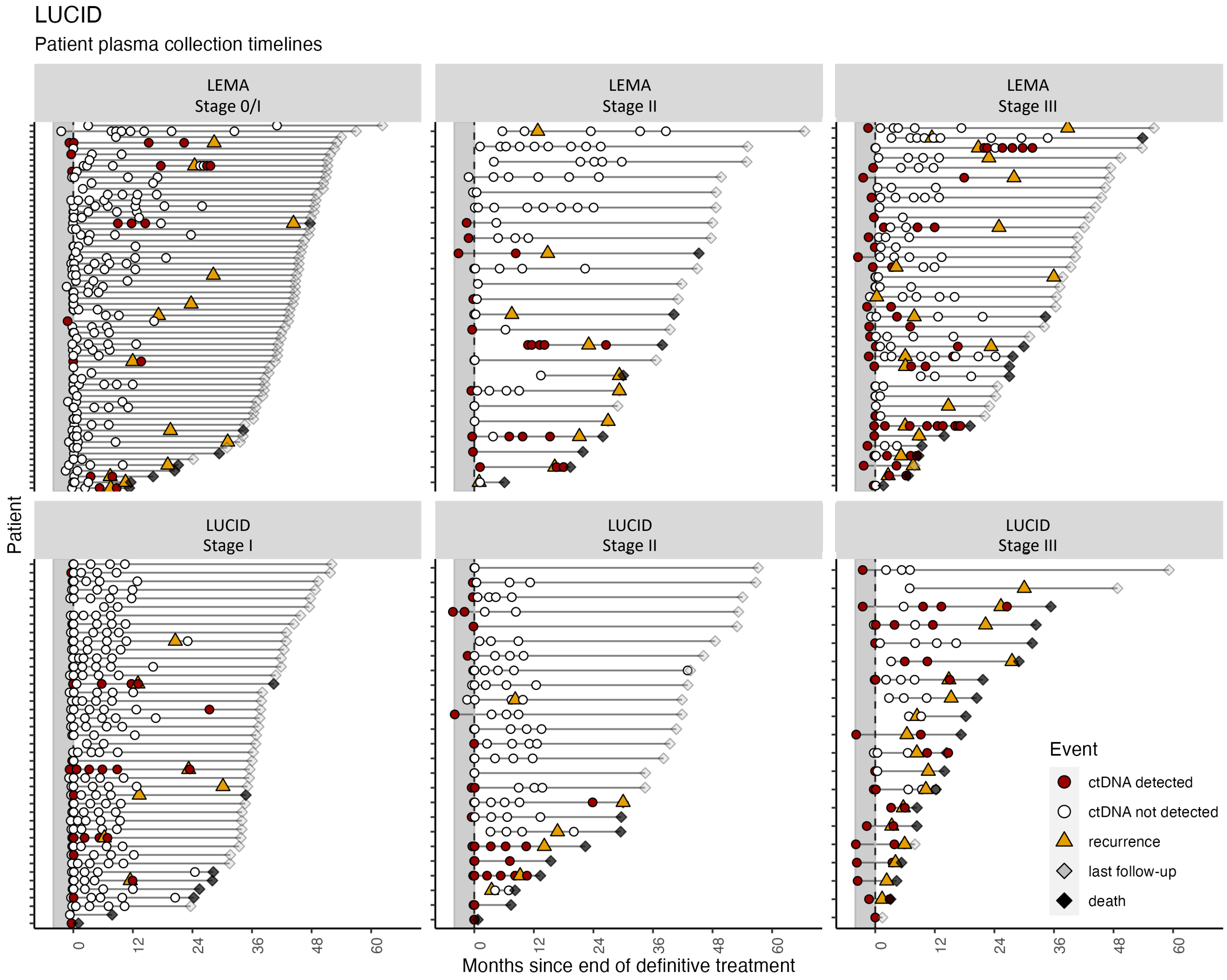

S2 Fig (D) Longitudinal plasma monitoring in LEMA and LUCID patients. Indicates when ctDNA was detected (red dots) or not detected (white dots). Clinical recurrence is indicated with an orange triangle. Time is measured from end of curative treatment (day 0) until end of follow-up (grey diamond).
