# Supplementary material for: Recurrence prediction using circulating tumor DNA in patients with early-stage non-small cell lung cancer after treatment with curative intent: A retrospective validation study
Source: PLoS Med. 2025 Apr 15;22(4):e1004574. doi: 10.1371/journal.pmed.1004574 (PMC12021277; doi:10.1371/journal.pmed.1004574)
Supplement: S3 Fig — Summary of ctDNA detection in the months following landmark, in patients with any stage (A) or stage II–III (B) disease. Time is considered as 6-month windows after landmark (0–6 months, 6–12 months, etc.). The top row indicates the raw count of patients with at least one ctDNA positive sample (red) or no ctDNA positive sample(s) (white), regardless of whether ctDNA and/or recurrence had occurred before that point. The bottom row shows the same data as a percentage of the total number of patients. (PDF) [file pmed.1004574.s017.pdf]

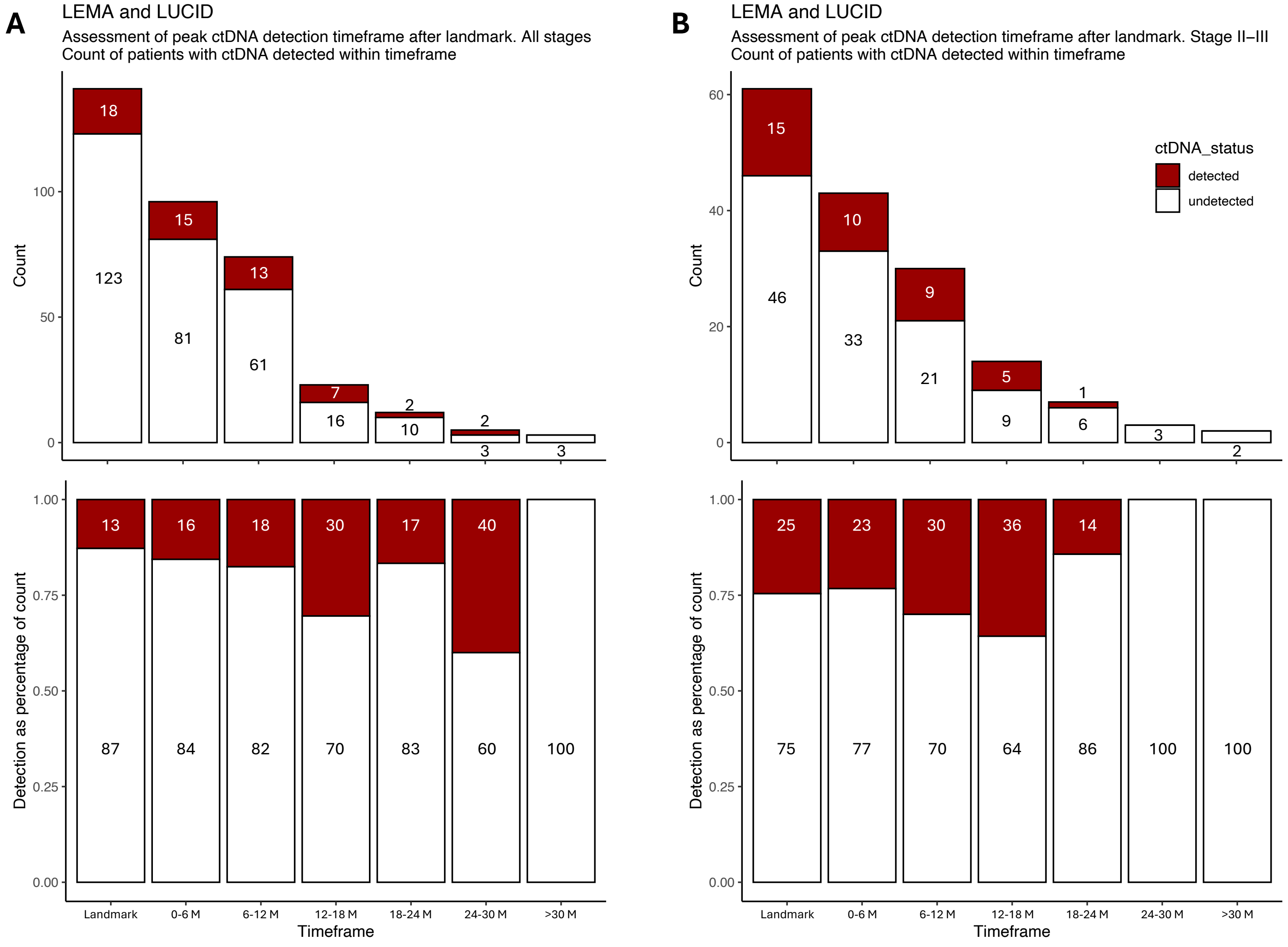

S3 Fig Summary of ctDNA detection in the months following landmark, in patients with any stage **(A)** or stage II-III **(B)** disease. Time is considered as six month windows after landmark (0-6 months, 6-12 months, etc). The top row indicates the raw count of patients with at least one ctDNA positive sample (red) or no ctDNA positive sample(s) (white), regardless of whether ctDNA and/or recurrence had occurred before that point. The bottom row shows the same data as a percentage of the total number of patients.
