# Supplementary material for: Recurrence prediction using circulating tumor DNA in patients with early-stage non-small cell lung cancer after treatment with curative intent: A retrospective validation study
Source: PLoS Med. 2025 Apr 15;22(4):e1004574. doi: 10.1371/journal.pmed.1004574 (PMC12021277; doi:10.1371/journal.pmed.1004574)
Supplement: S4 Fig — Associations are depicted as forest plots with hazard ratio (HR), 95% confidence interval and p-values indicated for each variable. The number of observations in each ‘category’ are indicated in parentheses. Data are based on the combined LEMA and LUCID cohorts. Multivariable analysis of clinical covariates and ctDNA detection at baseline (pre-treatment), with (A) recurrence-free survival (RFS) and (B) overall survival (OS). The equivalent data are presented considering detection at landmark (C, D), and all longitudinal sampling (E, D). (PDF) [file pmed.1004574.s018.pdf]

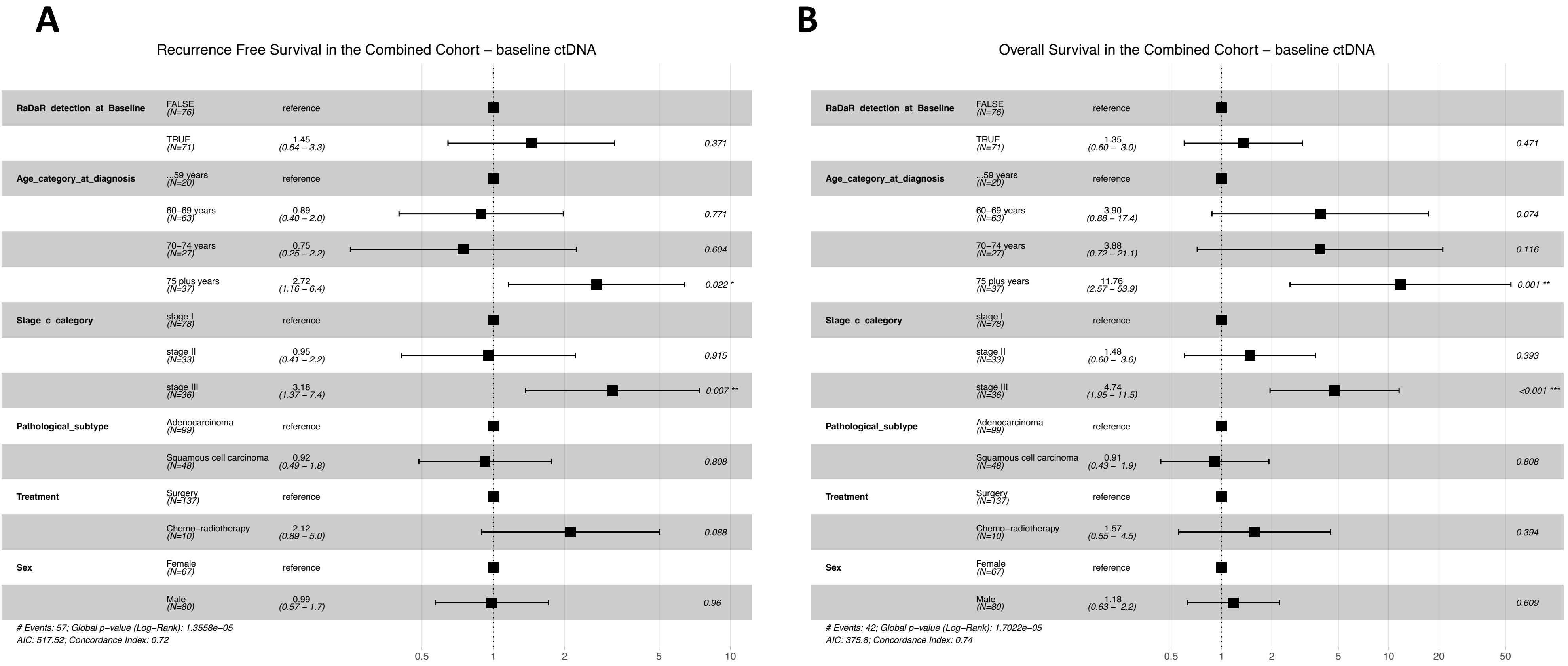

S4 Fig Multivariable analysis of survival and association with ctDNA detection at select timepoints

Cox regression analysis exploring the association of multiple covariables with survival. Associations are depicted as forest plots with hazard ratio (HR), 95% confidence interval and p-values indicated for each variable. The number of observations in each 'category' are indicated in parentheses. Data are based on the combined LEMA and LUCID cohorts.

Multivariable analysis of clinical covariates and ctDNA detection at baseline, with **(A)** recurrence free survival (RFS) and **(B)** overall survival (OS). The equivalent data are presented considering detection at landmark **(C and D)**, and all longitudinal sampling **(E and D)**.

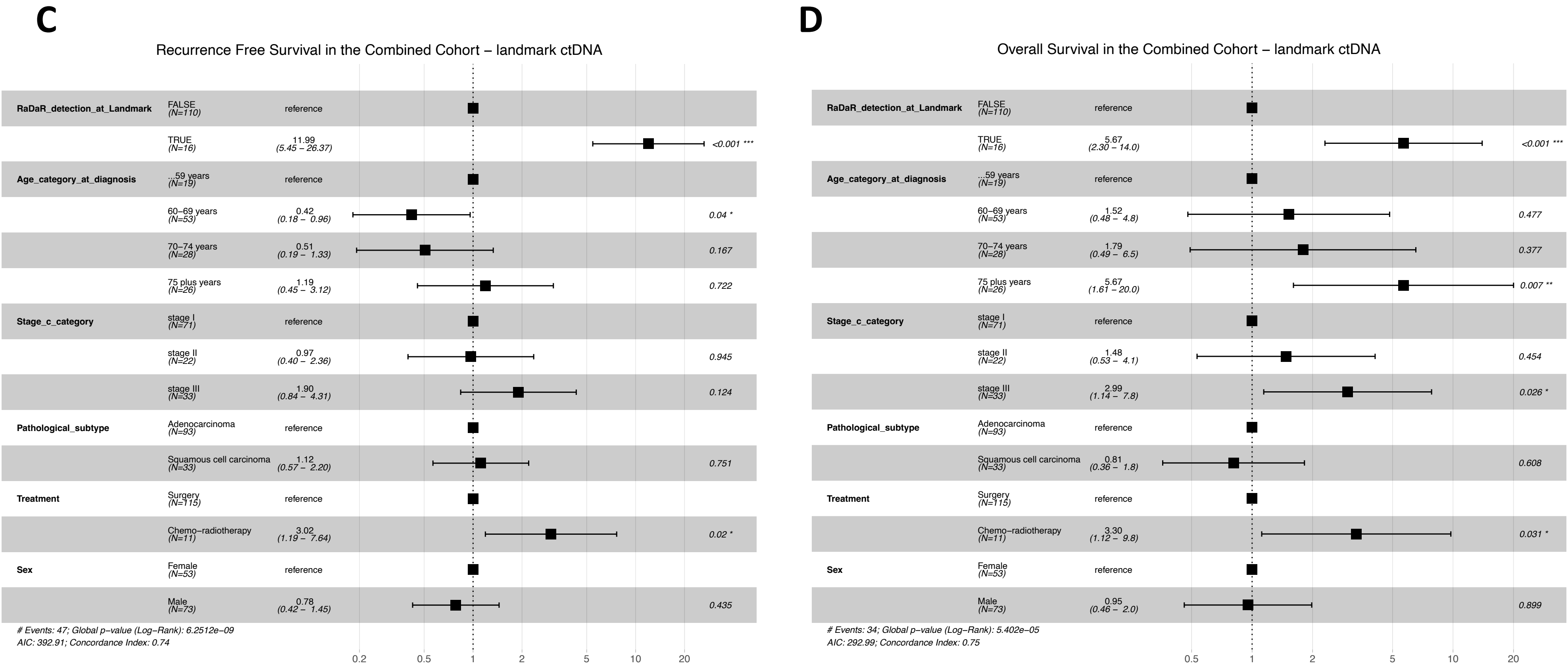

S4 Fig Multivariable analysis of survival and association with ctDNA detection at select timepoints

Cox regression analysis exploring the association of multiple covariables with survival. Associations are depicted as forest plots with hazard ratio (HR), 95% confidence interval and p-values indicated for each variable. The number of observations in each ‘category’ are indicated in parentheses. Data are based on the combined LEMA and LUCID cohorts.

Multivariable analysis of clinical covariates and ctDNA detection at baseline, with **(A)** recurrence free survival (RFS) and **(B)** overall survival (OS). The equivalent data are presented considering detection at landmark **(C and D)**, and all longitudinal sampling **(E and D)**.

E

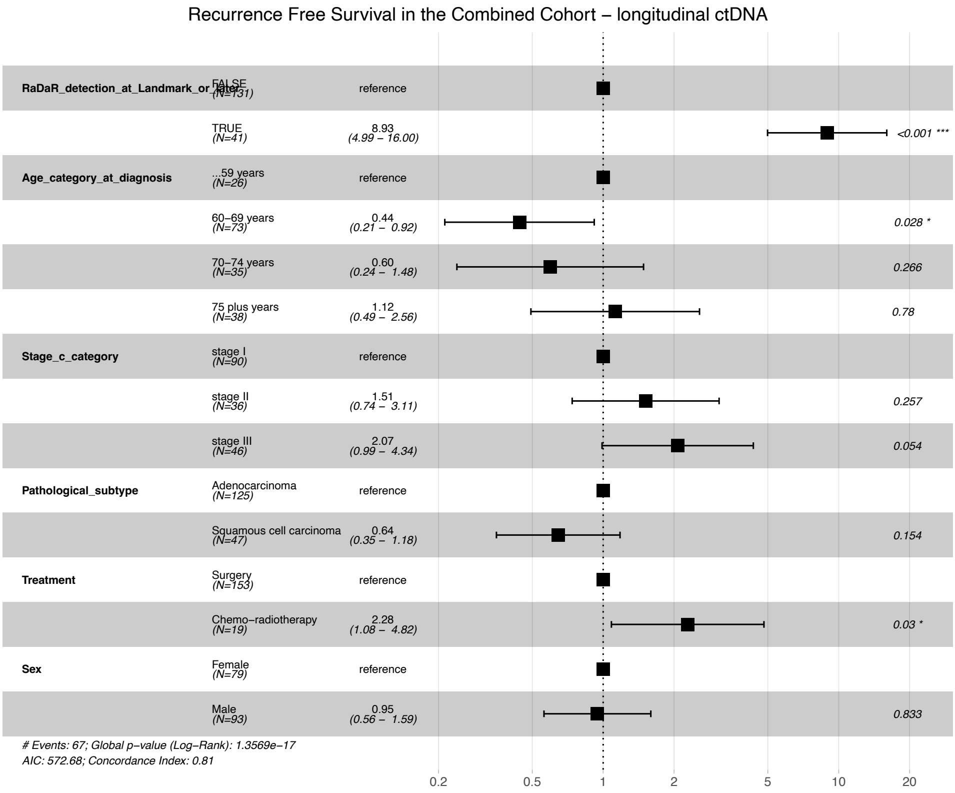

F

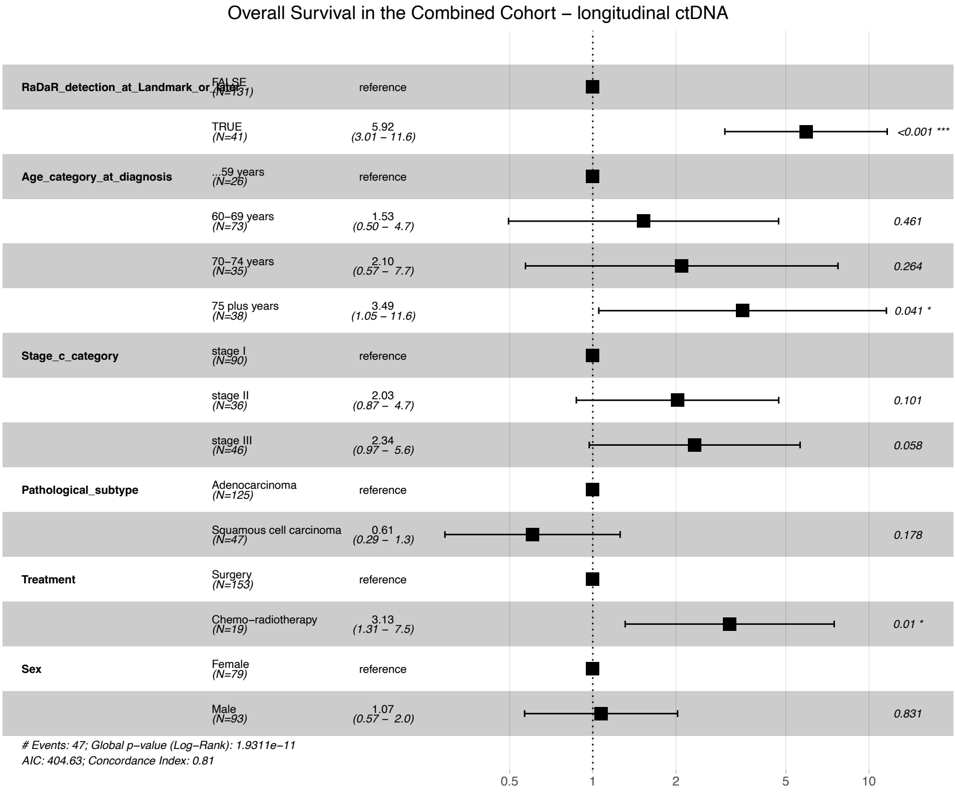

S4 Fig Multivariable analysis of survival and association with ctDNA detection at select timepoints

Cox regression analysis exploring the association of multiple covariables with survival. Associations are depicted as forest plots with hazard ratio (HR), 95% confidence interval and p-values indicated for each variable. The number of observations in each ‘category’ are indicated in parentheses. Data are based on the combined LEMA and LUCID cohorts.

Multivariable analysis of clinical covariates and ctDNA detection at baseline, with **(A)** recurrence free survival (RFS) and **(B)** overall survival (OS). The equivalent data are presented considering detection at landmark **(C and D)**, and all longitudinal sampling **(E and D)**.
