# Supplementary material for: Recurrence prediction using circulating tumor DNA in patients with early-stage non-small cell lung cancer after treatment with curative intent: A retrospective validation study
Source: PLoS Med. 2025 Apr 15;22(4):e1004574. doi: 10.1371/journal.pmed.1004574 (PMC12021277; doi:10.1371/journal.pmed.1004574)
Supplement: S5 Fig — Kaplan–Meier analysis showing the fraction of patients without events as a function of time. Patient subgroups are defined based on ctDNA detection at eVAF <0.01% or <0.008% (blue) vs. ctDNA not detected (yellow) at the pre-treatment time point. The number of patients remaining at risk are shown below each graph. (A) Recurrence-free survival (RFS) and (B) overall survival (OS) for combined LEMA and LUCID patients split by ctDNA detection at eVAF < 0.01% vs. ND. (C, D) eVAF < 0.008% vs ND. (E, F) eVAF < 0.01% in LUAD patients only. ND, not detected; LUAD, lung adenocarcinoma. (PDF) [file pmed.1004574.s019.pdf]

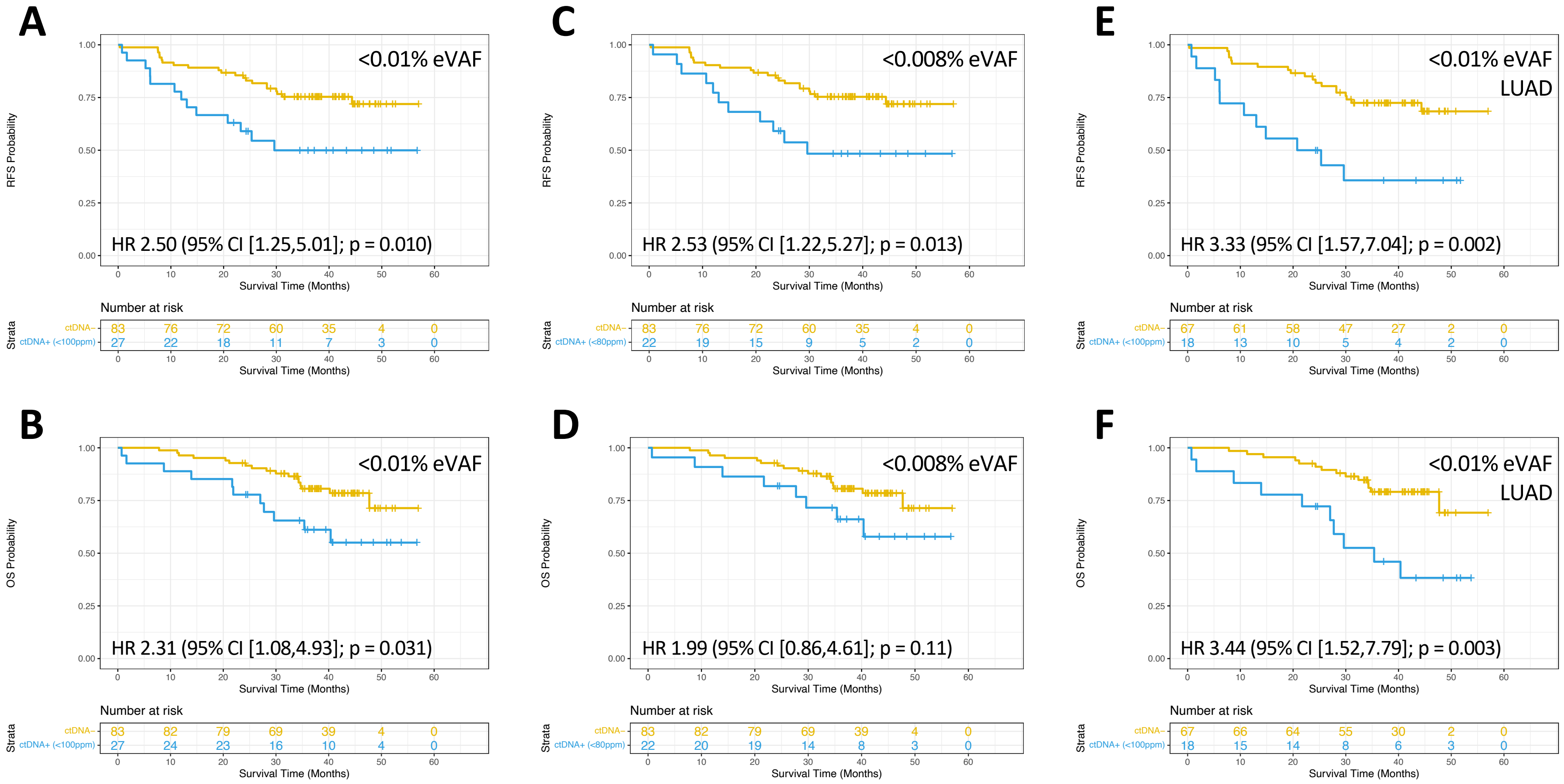

S5 Fig Survival analysis based on pre-treatment ctDNA detection at low eVAF vs. ctDNA not detected

Kaplan-Meier analysis showing the fraction of patients without events as a function of time. Patient subgroups are defined based on ctDNA detection at eVAF <0.01% or <0.008% (blue) vs. ctDNA not detected (yellow) at the pre-treatment timepoint. The number of patients remaining at risk are shown below each graph.

(A) Recurrence-free survival (RFS) and **(B)** overall survival (OS) for combined LEMA and LUCID patients split by ctDNA detection at eVAF <0.01% vs. ND **(C and D)** eVAF <0.008% vs ND. **(E and F)** eVAF < 0.01% in LUAD patients only. *ND = not detected; LUAD = lung adenocarcinoma*
