# Supplementary material for: Recurrence prediction using circulating tumor DNA in patients with early-stage non-small cell lung cancer after treatment with curative intent: A retrospective validation study
Source: PLoS Med. 2025 Apr 15;22(4):e1004574. doi: 10.1371/journal.pmed.1004574 (PMC12021277; doi:10.1371/journal.pmed.1004574)
Supplement: S6 Fig — Kaplan–Meier analysis showing the fraction of patients without events as a function of time. Patient subgroups are defined based on ctDNA detection in samples collected within 1–3 days after curative treatment. Patients with ctDNA detected are shown by blue lines, and those with ctDNA not detected are shown by yellow lines. The number of patients remaining at risk are shown below each graph. (A) Recurrence-free survival (RFS) and (B) overall survival (OS) for LEMA patients split by ctDNA detection within 1–3 days after the end of curative treatment. (C, D) for LUCID patients. (E, F) for the combined cohorts. (PDF) [file pmed.1004574.s020.pdf]

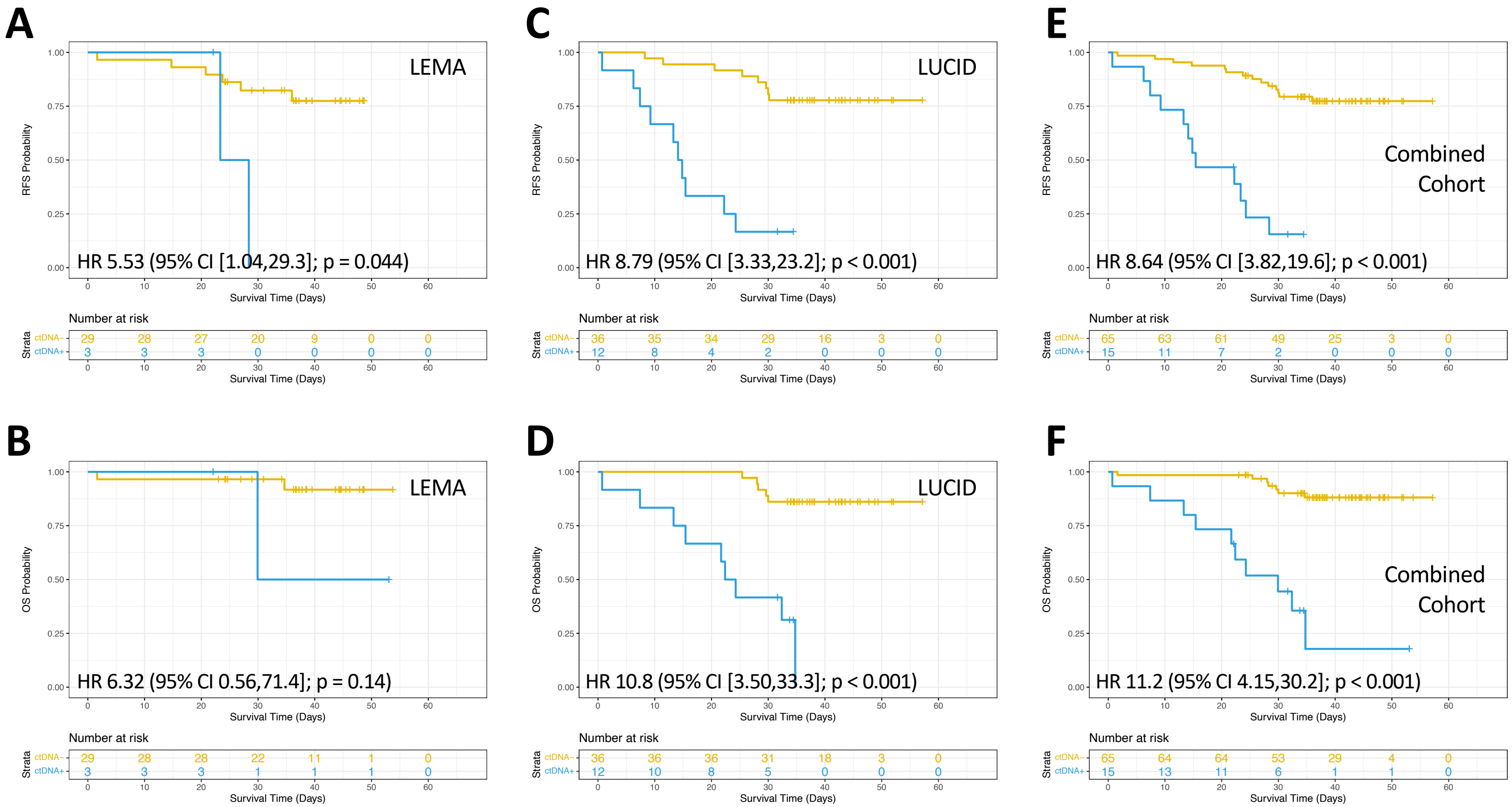

S6 Fig Survival analysis based on ctDNA detection in plasma collected within 1-3 days after curvative treatment

Kaplan-Meier analysis showing the fraction of patients without events as a function of time. Patient subgroups are defined based on ctDNA detection in samples collected within 1-3 days after curative treatment. Patients with ctDNA detected are shown by blue lines, and those with ctDNA not detected are shown by yellow lines. The number of patients remaining at risk are shown below each graph.

(A) Recurrence-free survival (RFS) and **(B)** overall survival (OS) for LEMA patients split by ctDNA detection within 1-3 days after the end of curative treatment. **(C and D)** for LUCID patients. **(E and F)** for the combined cohorts
