# Supplementary material for: Recurrence prediction using circulating tumor DNA in patients with early-stage non-small cell lung cancer after treatment with curative intent: A retrospective validation study
Source: PLoS Med. 2025 Apr 15;22(4):e1004574. doi: 10.1371/journal.pmed.1004574 (PMC12021277; doi:10.1371/journal.pmed.1004574)
Supplement: S7 Fig — Recurrence free survival analysis of the combined cohort for patients split by ctDNA detection at any time point ≥2 weeks after the end of curative treatment, categorized by stage 0/I (A, n = 98), stage II (B, n = 39) and stage III (C, n = 52). Shown are equivalent data for Overall survival analysis (D, E, F), respectively. (PDF) [file pmed.1004574.s021.pdf]

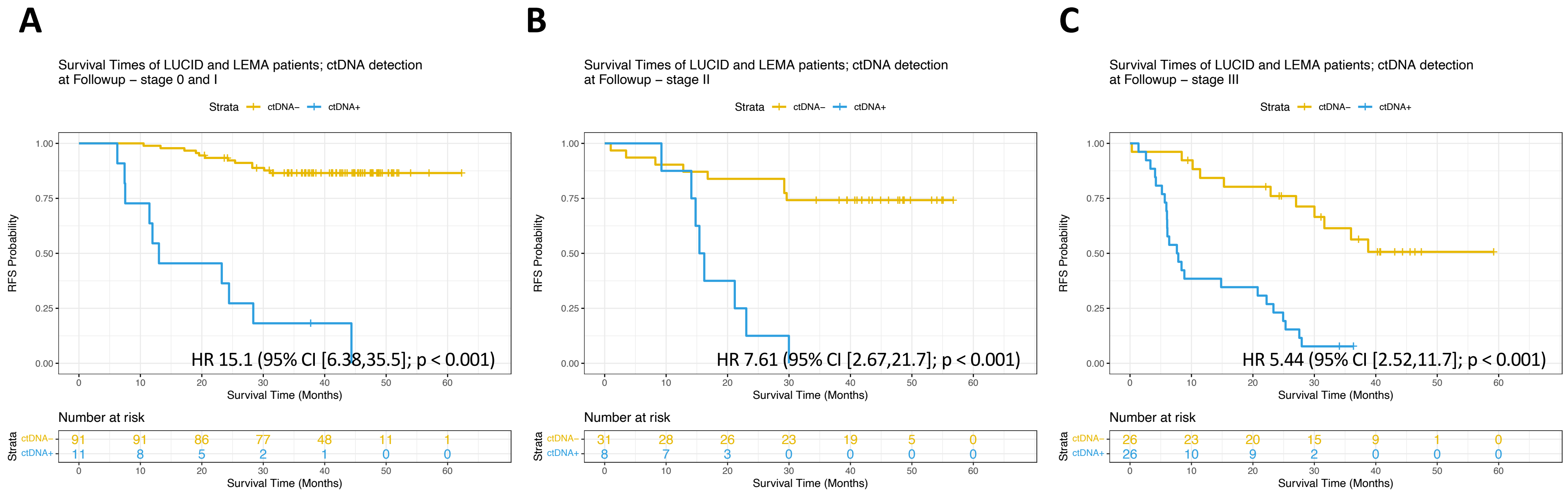

S7 Fig Survival analysis based on ctDNA detection categorized by stage in the combined dataset

Recurrence free survival analysis of the combined cohort for patients split by ctDNA detection at any timepoint ≥2 weeks after the end of curative treatment, categorized by stage 0/I (**A,** n=98), stage II **(B**, n=39) and stage III (**C**, n=52)

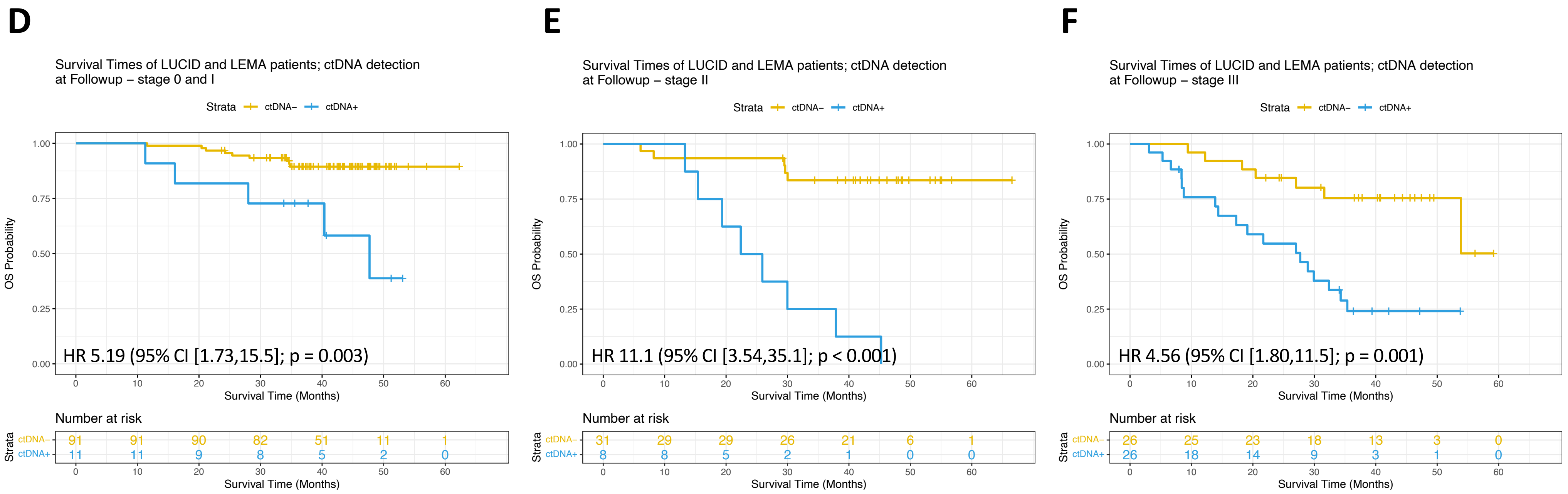

S7 Fig Survival analysis based on ctDNA detection categorized by stage in the combined dataset

Overall survival analysis of the combined cohort for patients split by ctDNA detection at any timepoint ≥2 weeks after the end of curative treatment, categorized by stage 0/I (**D**, n=98), stage II (**E**, n=39) and stage III (**F**, n=52).
