# Supplementary material for: Recurrence prediction using circulating tumor DNA in patients with early-stage non-small cell lung cancer after treatment with curative intent: A retrospective validation study
Source: PLoS Med. 2025 Apr 15;22(4):e1004574. doi: 10.1371/journal.pmed.1004574 (PMC12021277; doi:10.1371/journal.pmed.1004574)
Supplement: S8 Fig — Recurrence survival analysis of the combined cohort for patients split by ctDNA detection at pretreatment (A, B), or at any time point ≥2 weeks after the end of curative treatment (C, D), categorized by tumor histology; Adenocarcinoma (A, n = 102; C, n = 132), Squamous Cell Carcinoma (B, n = 48; D, n = 47). The equivalent Overall survival analysis data are also shown, with ctDNA detection at pretreatment (E, F), or at any time point ≥2 weeks after the end of curative treatment (G, H), categorized by Adenocarcinoma (E, n = 102; G, n = 132), Squamous Cell Carcinoma (F, n = 48; H, n = 47). (PDF) [file pmed.1004574.s022.pdf]

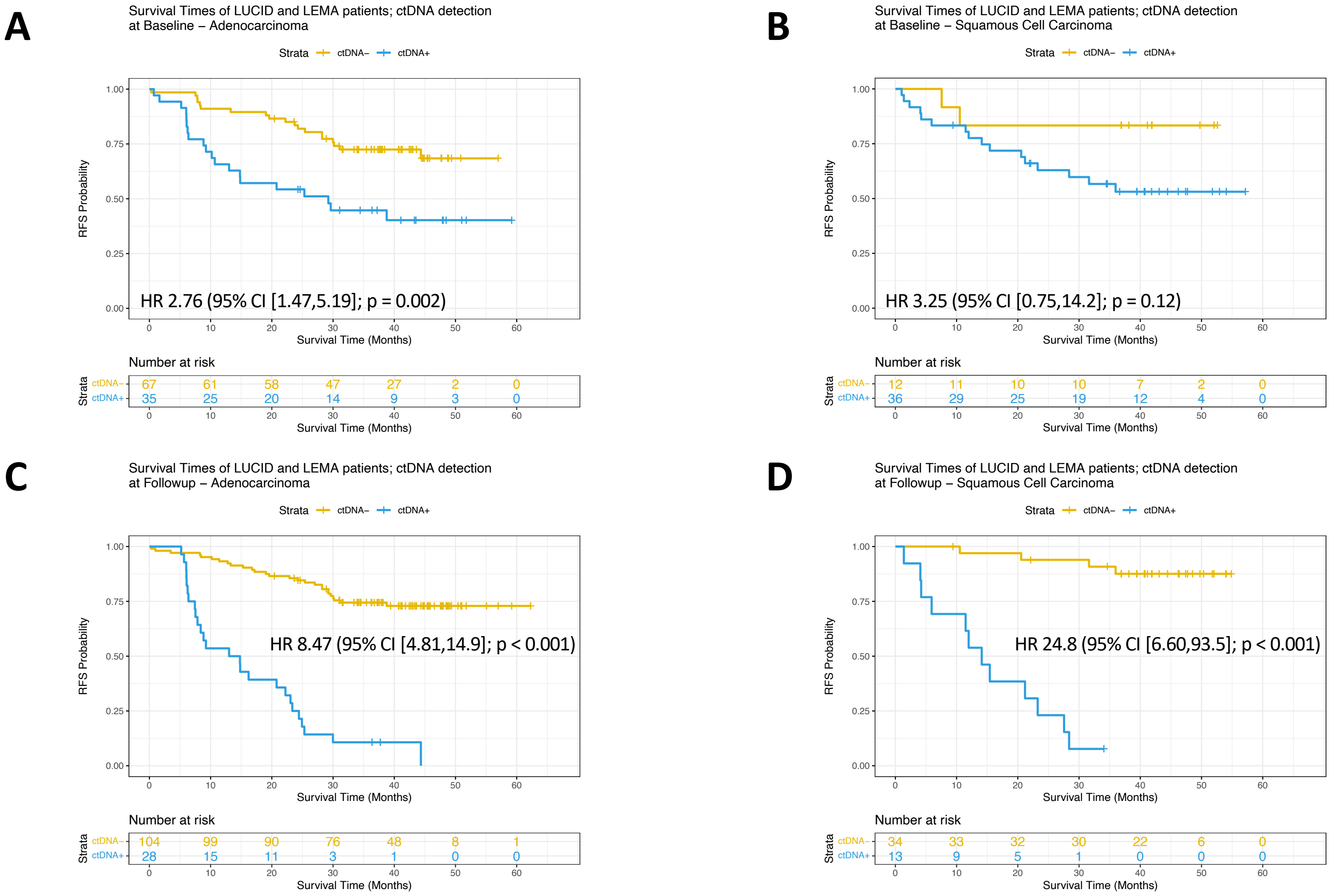

S8 Fig Survival analysis based on ctDNA detection categorized by tumor histology

Recurrence survival analysis of the combined cohort for patients split by ctDNA detection at pretreatment **(A, B)**, or at any timepoint ≥2 weeks after the end of curative treatment **(C, D)**, categorized by tumor histology; Adenocarcinoma (**A**, n=102; **C**, n=132), Squamous Cell Carcinoma (**B**, n=48; **D**, n=47)

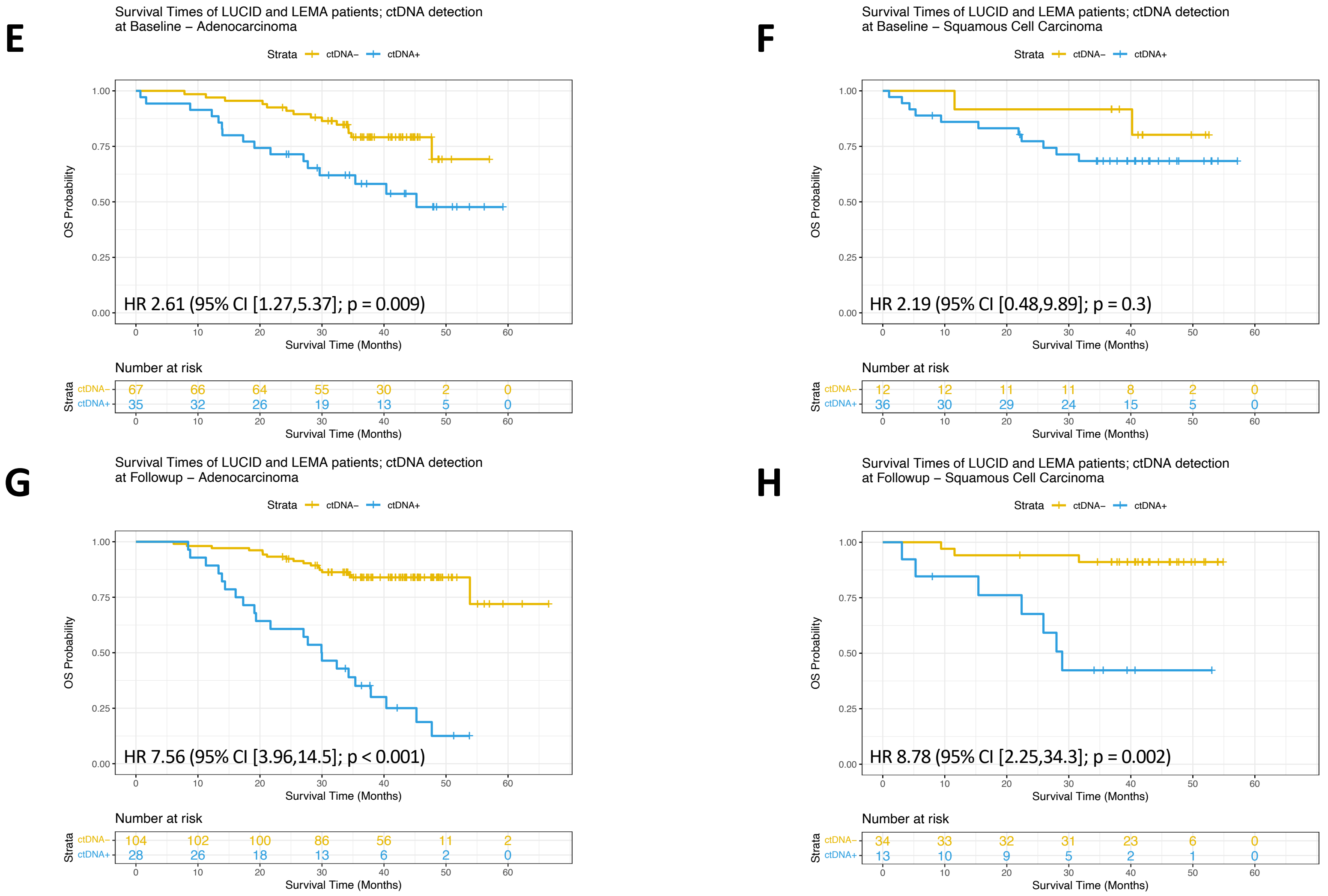

S8 Fig Survival analysis based on ctDNA detection categorized by tumor histology

Overall survival analysis of the combined cohort for patients split by ctDNA detection at pretreatment **(E, F)**, or at any timepoint ≥2 weeks after the end of curative treatment **(G, H)**, categorized by tumor histology; Adenocarcinoma (**E**, n=102; **G**, n=132), Squamous Cell Carcinoma (**F**, n=48; **H**, n=47)
