# Supplementary material for: Recurrence prediction using circulating tumor DNA in patients with early-stage non-small cell lung cancer after treatment with curative intent: A retrospective validation study
Source: PLoS Med. 2025 Apr 15;22(4):e1004574. doi: 10.1371/journal.pmed.1004574 (PMC12021277; doi:10.1371/journal.pmed.1004574)
Supplement: S9 Fig — Kaplan–Meier analysis showing the fraction of patients without events as a function of time. Patient subgroups are defined based on ctDNA detection at different time windows. Patients with ctDNA detected are shown by blue lines, and those with ctDNA not detected are shown by yellow lines. The number of patients remaining at risk are shown below each graph. (A) RFS and (B) OS including only patients with positive ctDNA pretreatment, split by ctDNA detection at any time point ≥14 days after the end of curative treatment (n = 69). (C) RFS and (D) OS including only patients with positive ctDNA pretreatment, split by ctDNA detection at the landmark time point, which is the first plasma sample available in the window of ≥2 weeks and ≤4 months after the end of curative treatment (n = 49). (PDF) [file pmed.1004574.s023.pdf]

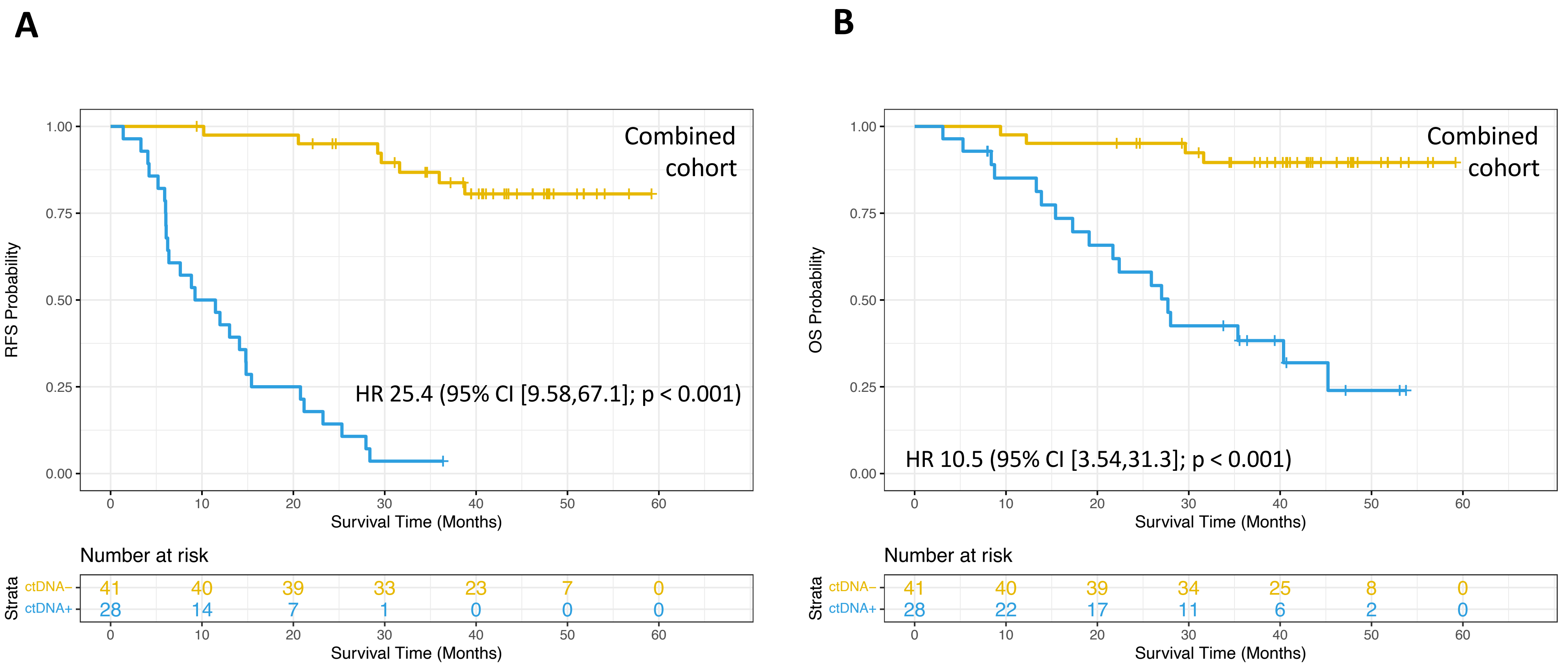

S9 Fig Survival analysis based on ctDNA detection in patients with positive ctDNA pretreatment in the combined dataset
Kaplan-Meier analysis showing the fraction of patients without events as a function of time. Patient subgroups are defined based on ctDNA detection at different time windows. Patients with ctDNA detected are shown by blue lines , and those with ctDNA not detected are shown by yellow lines. The number of patients remaining at risk are shown below each graph.
(A) RFS and **(B)** OS including only patients with positive ctDNA pretreatment, split by ctDNA detection at any timepoint ≥14 days after the end of curative treatment (n=69)

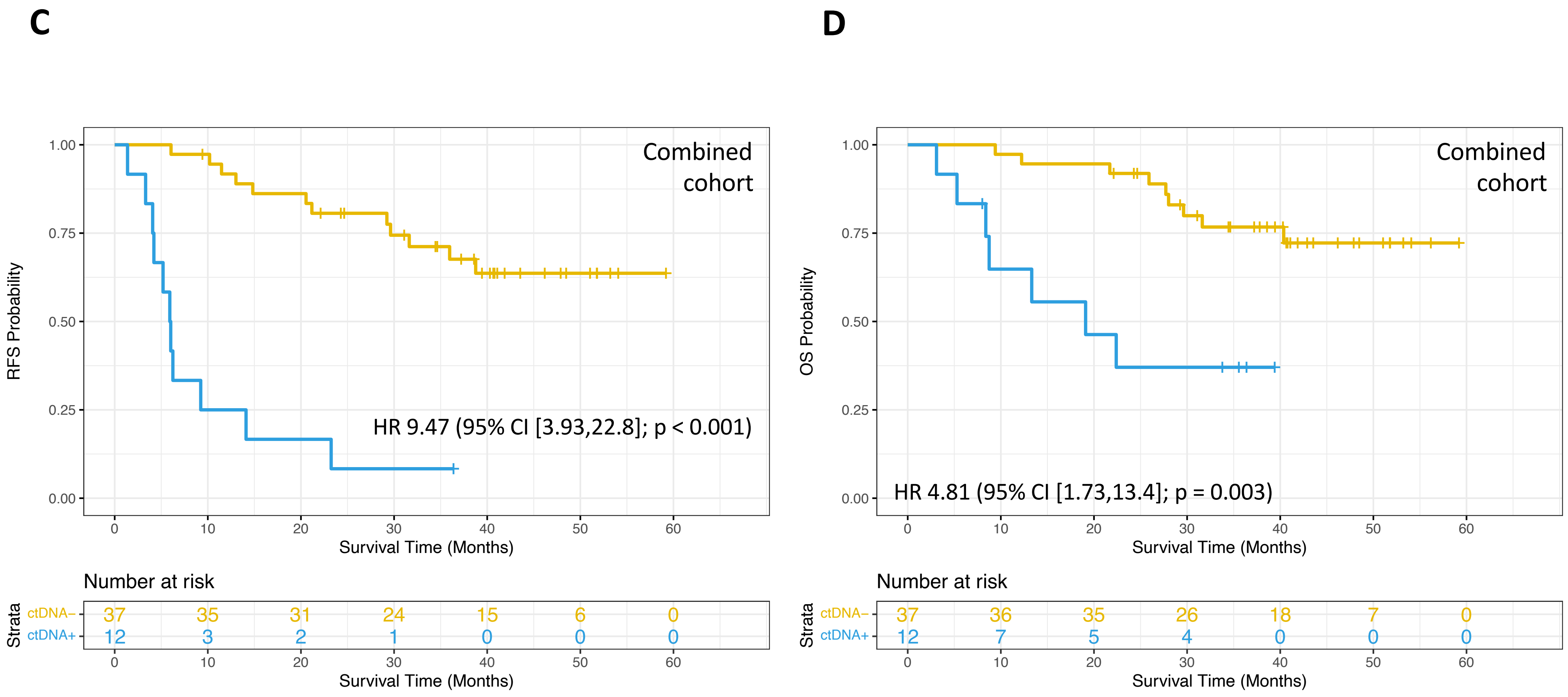

S9 Fig Survival analysis based on ctDNA detection in patients with positive ctDNA pretreatment in the combined dataset
(C) RFS and **(D)** OS including only patients with positive ctDNA pretreatment, split by ctDNA detection at the landmark timepoint, which is the first plasma sample available in the window of ≥ 2 weeks and ≤ 4 months after the end of curative treatment (n=49)
