# Supplementary material for: Recurrence prediction using circulating tumor DNA in patients with early-stage non-small cell lung cancer after treatment with curative intent: A retrospective validation study
Source: PLoS Med. 2025 Apr 15;22(4):e1004574. doi: 10.1371/journal.pmed.1004574 (PMC12021277; doi:10.1371/journal.pmed.1004574)
Supplement: S10 Fig — (A) RFS and (B) OS stratified by, split by ctDNA detection at the landmark and pretreatment time points (n = 105). (PDF) [file pmed.1004574.s024.pdf]

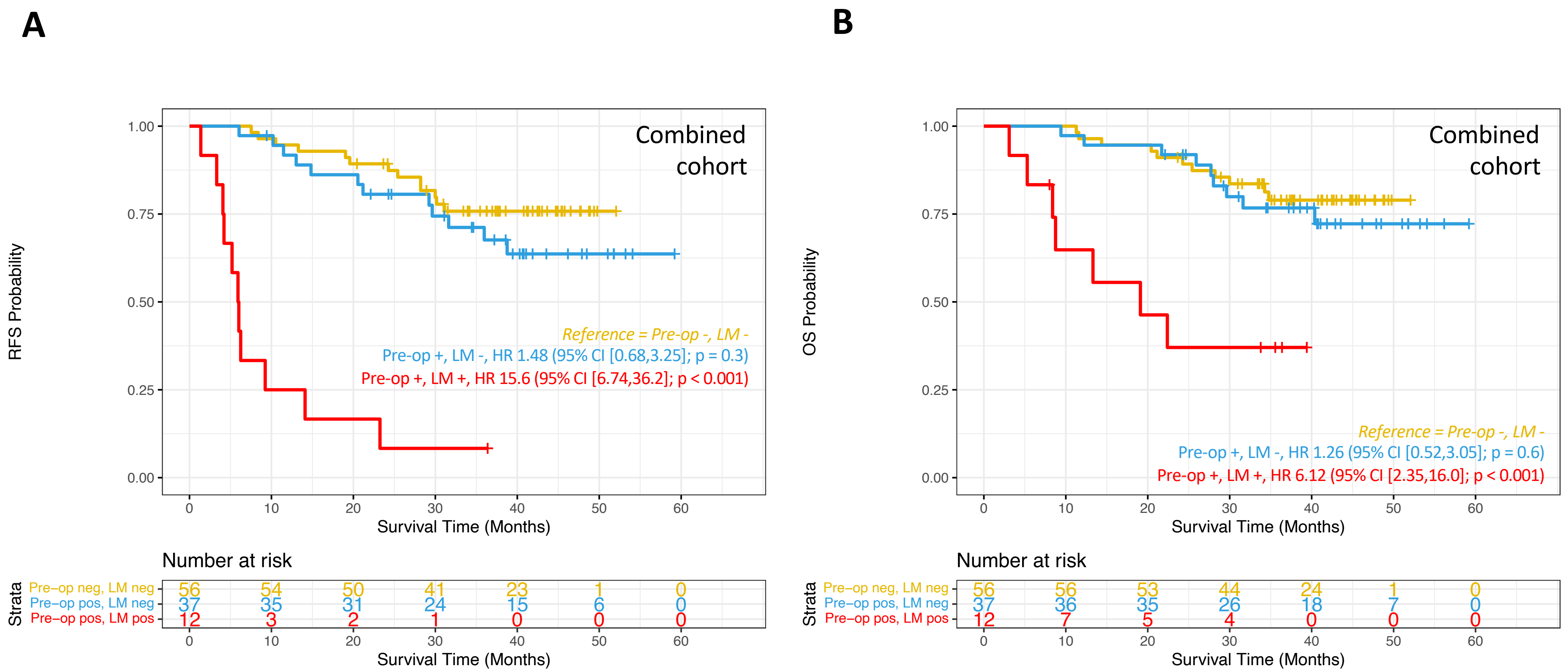

S10 Fig Survival analysis of the predictive value of pretreatment ctDNA in patients that were ctDNA negative at the landmark timepoint in the combined dataset

(A) RFS and **(B)** OS stratified by detection at the landmark and pretreatment timepoints (n=105)
