# Supplementary material for: Recurrence prediction using circulating tumor DNA in patients with early-stage non-small cell lung cancer after treatment with curative intent: A retrospective validation study
Source: PLoS Med. 2025 Apr 15;22(4):e1004574. doi: 10.1371/journal.pmed.1004574 (PMC12021277; doi:10.1371/journal.pmed.1004574)
Supplement: S11 Fig — Evaluation of tumor variants that were successfully included in the ctDNA panel vs. the assay input in copies. The dashed vertical lines indicate the minimum (2,000 copies) and maximum (20,000 copies) input amounts for RaDaR. The dashed horizontal lines indicate the minimum (8) and maximum (48) number of variants targeted per panel. Diagonal lines represent the lower limit of detection (LoD). Only samples of patients with negative ctDNA while developing recurrence (i.e., potential false negative samples; FN, represented by the red points) and samples of patients with positive ctDNA while not developing recurrence (i.e., potential false positive samples; FP, represented by the blue points) are shown. (A) Samples included in the landmark timeframe. (B) Samples collected during the follow-up period starting ≥14 days after the end date of curative treatment. (PDF) [file pmed.1004574.s025.pdf]

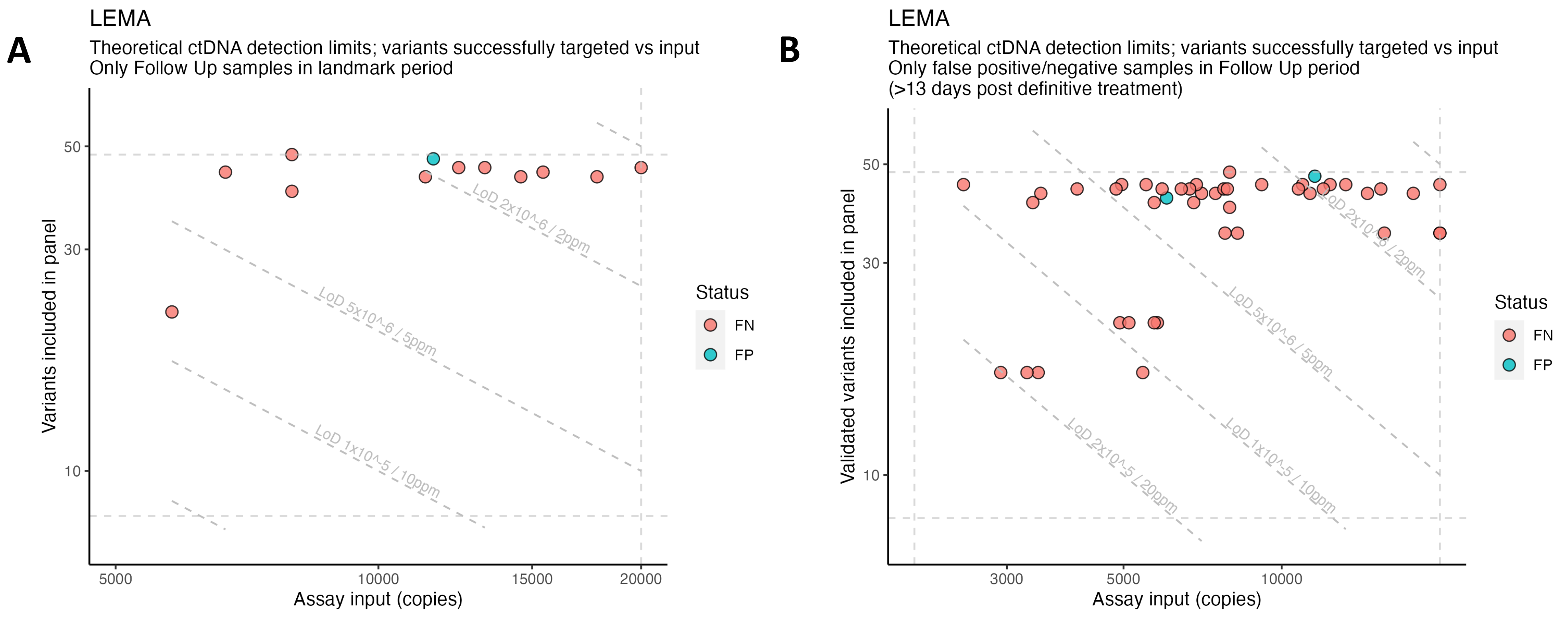

S11 Fig Theoretical ctDNA detection limits

Evaluation of tumor variants that were succesfully included in the ctDNA panel versus the assay input in copies. The dashed vertical lines indicate the minimum (2,000 copies) and maximum (20,000 copies) input amounts for RaDaR. The dashed horizontal lines indicate the minimum (8) and maximum (48) number of variants targeted per panel. Diagonal lines represent the lower limit of detection (LoD). Only samples of patients with negative ctDNA while developing recurrence (*i.e.* potential false negative samples; FN, represented by the red dots) and samples of patients with positive ctDNA while not developing recurrence (*i.e.* potential false positive samples; FP, represented by the blue dots) are shown.

(A) Samples included in the landmark timeframe

(B) Samples collected during the follow-up period starting ≥14 days after the end date of curative treatment
