# Supplementary material for: Recurrence prediction using circulating tumor DNA in patients with early-stage non-small cell lung cancer after treatment with curative intent: A retrospective validation study
Source: PLoS Med. 2025 Apr 15;22(4):e1004574. doi: 10.1371/journal.pmed.1004574 (PMC12021277; doi:10.1371/journal.pmed.1004574)
Supplement: S12 Fig — Exploration of the relationship between tumor volume (mm3) and ctDNA levels (eVAF, %) at baseline. Note, tumor volumetric data were only available for the LUCID cohort. Disease stage is indicated by point color. (PDF) [file pmed.1004574.s026.pdf]

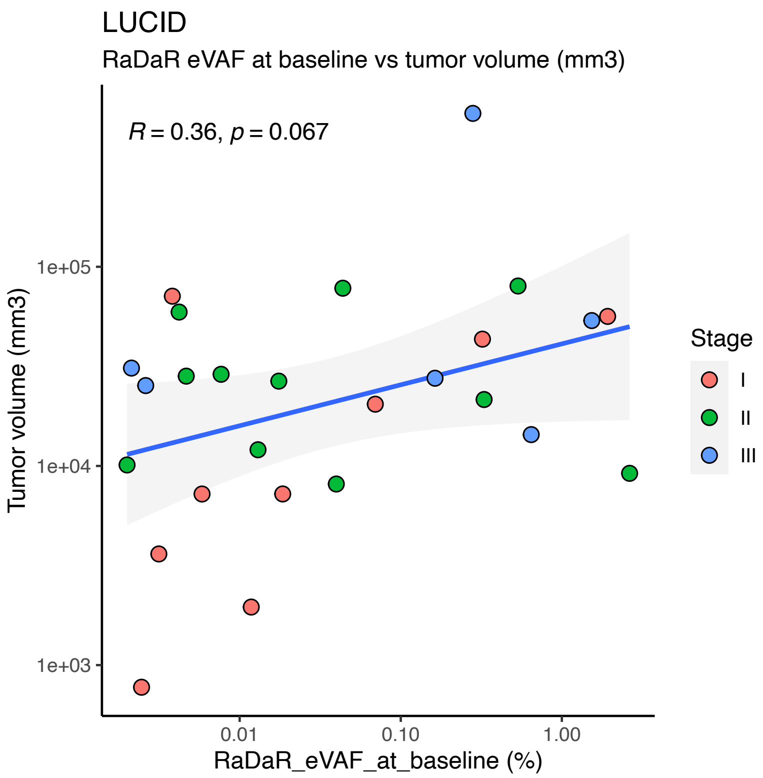

S12 Fig Comparison of tumor volume with ctDNA levels at baseline

Exploration of the relationship between tumor volume (mm3) and ctDNA levels (eVAF, %) at baseline. Note, tumor volumetric data were only available for the LUCID cohort. Disease stage is indicated by point color.
