# Supplementary material for: Recurrence prediction using circulating tumor DNA in patients with early-stage non-small cell lung cancer after treatment with curative intent: A retrospective validation study
Source: PLoS Med. 2025 Apr 15;22(4):e1004574. doi: 10.1371/journal.pmed.1004574 (PMC12021277; doi:10.1371/journal.pmed.1004574)
Supplement: S1 Methods — Also includes a discussion of the value of tumor-informed vs. tumor-agnostic approaches to ctDNA detection, and impact on clinical performance. (DOCX) [file pmed.1004574.s027.docx]

# S1 Methods

Sample collection
The wider LEMA study aimed to collect blood samples during the following time windows: baseline (day -60 to 0 relative to the start of first line treatment), first follow-up (2-42 days after completion of curative intended treatment) and in follow-up (every 3 months ± 1 month). However, strict sample collection proved challenging since blood was only collected in combination with a regular blood withdrawal during scheduled visits at the outpatient clinic. Therefore, sampling often did not precisely adhere to the above schedule.

Blood specimens were collected using either 10 mL K2‐EDTA tubes or 10 mL cell‐stabilising tubes (CST; STRECK, Omaha, NE, USA). Plasma cell-free DNA (cfDNA) was obtained from the K2‐EDTA tube within 4 hours by a two‐step centrifugation protocol carried out at room temperature: 20 min at 380 g followed by 10 min at 20,000 g. CST were centrifuged at room temperature for 10 min at 1700 g and 10 min at 20,000 g within 7 days. Plasma cfDNA was extracted using the Qiamp Circulating Nucleic Acid kit (Qiagen), and stored in 1–4 mL aliquots at −80 °C. Buffy coat samples, isolated from whole blood after the first centrifugation step, had germline DNA extracted using the QIAamp DNA Blood Mini Kit or QIAsymphony DSP Circulating DNA kit (Qiagen).

Tissue processing and analysis by whole exome sequencing
DNA was extracted from FFPE tissue sections using the QIAamp® DNA FFPE Tissue Kit (Qiagen) with modifications and DNA repair. Whole exome sequencing (WES) of sheared (200-300bp) DNA was carried out following the Human IDT Target Enrichment Protocol (Integrated DNA Technologies; IDT). Mutation calling was carried out using Mutect2 (MuTect2 v3.831,32).

RaDaR personalized ctDNA sequencing assay analysis
The RaDaR assay is based upon personalized multiplex PCR amplification of cfDNA.

Tumour-specific variants, identified by exome sequencing of primary tumour, were ranked and prioritized for inclusion into custom panels, incorporating 48 amplicons per patient, targeting patient-specific variants. Variant prioritization and selection criteria take into consideration the variant set most suitable for the sensitive detection of ctDNA in the patient for whom the assay was designed as well as ensure that the selected primer pairs are well suited for multiplex PCR, allowing the efficient amplification of the target ctDNA. For each patient, a single individualized panel of primers (IDT) was created, covering patient-specific variants, and combined with a fixed primer panel covering common population-specific single nucleotide polymorphisms, for internal sample quality control during NGS testing. Between 500 and 2,000 amplifiable copies of tumour DNA, as well as 500 amplifiable copies of DNA extracted from buffy coat from the respective patient, were used for panel qualification, as previously described.^1^ Results were used to confirm amplification of the target regions and the presence of selected single nucleotide variants (SNVs) in tumour DNA. Absent variants are typically WES false positives or variants that have failed to amplify, sequence or align to the target region. In addition, target SNVs were excluded if signal was observed in the matched buffy coat DNA, to reduce the potential impact of germline mutations, mosaicism or variants arising from clonal haematopoiesis of indeterminate potential. The median number of tumour-confirmed variants was 44 (range: 11–49), corresponding to a median of 92% confirmed variants (range: 23–100%; Supplementary Table S2), with 74% of samples having at least 40 confirmed variants. Panels with eight or more tumour-confirmed variants detected at sufficient read depth were considered as qualified.

After panel qualification, assays were applied to plasma cfDNA. The median input, as measured using a custom ddPCR assay, was 7,760 amplifiable copies per sample (range: 760–20,000 copies; Supplementary table S3). High-depth NGS of amplified libraries was carried out on the NovaSeq® 6000 system (Illumina) using PE150 sequencing, generating a median of 16,853,267 reads (excluding non-mappable reads and reads with Phred score <20) per sample [interquartile range (IQR) 11,435,245 to 26,315,472]. The median coverage for each variant was 218,700 reads per sample.

To obtain evidence for the presence or absence of ctDNA at the sample level, the statistical significance of the observed mutant counts for each of the variants remaining after panel qualification, was assessed using a statistical framework incorporating the entire set of personalized variants. This framework compared the sequencing counts of each variant to a model of noise for each individual variant and was locked before the analyses described in this study. A sample was classified as ‘ctDNA positive’ when its cumulative statistical score was above a pre-set threshold defined during the assay’s analytical development and validation of the locked assay[1].

A statistical model was used to assess the significance of the observed counts for each variant and the information was integrated over the entire set of personalized variants from an individual sample to obtain evidence of ctDNA presence (ctDNA positive) or absence (ctDNA negative) at the sample level. The tumour fraction for each sample was estimated and reported as an estimated VAF (eVAF; Supplementary table S3).

The same version of RaDaR (version 1) was used for both the LEMA and LUCID studies.

*Discussion of the value of tumor-informed vs tumor-agnostic approaches to ctDNA detection, and impact on clinical performance*

There is much discussion about the value of tumor-informed vs tumor-agnostic ctDNA assays. The former require up-front sequencing of tissue which has cost implications, requires access to sufficient quantity and quality of tissue, and at least a few days between tissue profiling and the assay being ready for use. However, tumor-informed approaches in-theory have greater sensitivity for ctDNA detection at low levels. This should translate to greater clinical performance (sensitivity and specificity). Ideally, we’d have been able to do a formal comparison between methods as part of this study, but this was not possible. We can however, comment on the theory behind these approaches and perform rudimentary analyses to predict the difference in analytical sensitivity between select methods.

Tumor agnostic approaches that detect genetic changes vary in size and methodology. Comprehensive Genomic Profiling (CGP) assays that target tens to hundreds of genes (e.g. the Illumina TSO500 assay that targets ~500 genes) might be expected to detect fewer variants in plasma – possibly single digits, depending on cancer type and disease stage. Conversely, the RaDaR assay can target tens of variants, identified by ‘up front’ tissue sequencing across the breadth of the exome. As depicted in S11 Fig, the LoD of an assay is conceptually the product of the number of variants targeted and the amount of DNA used as input to the assay. As such, the more variants that can be practically targeted, the greater the expected sensitivity.

To explore this, we scrutinised the LEMA dataset. Initially we wondered how many variants would have been identified in tissue had we used a TSO500-like gene panel for somatic profiling, instead of whole exome sequencing (WES). To this end, we filtered the WES data for variants lying in genes included in the gene panel. We found that the majority of patients would have had <10 variants identified, compared with several hundred identified by WES, as indicated in the below figure.


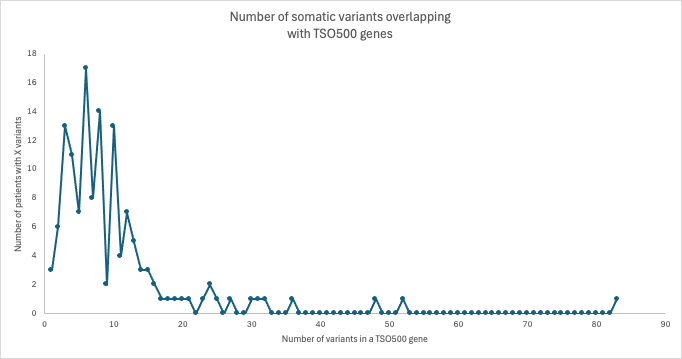


During previous analytical experiments, the ‘limit of detection’ (LoD) of the RaDaR assay was explored - an *in-silico* approach to LoD determination using bootstrap analysis was performed to estimate the LoD95 when targeting different numbers of variants for different DNA input levels. Select findings are presented below. This allows us to estimate the sensitivity of the RaDaR assay were we to target n=6 variants (which is the mode of the distribution shown above), vs. if we used a full RaDaR panel of n=48 variants.

|  |  |
| --- | --- |
| Panel Size | Input - 20,000 copies |
| 48 variants | 0.0014% |
| 6 variants | 0.0073% |

*Estimated limit of detection of RaDaR panels based on*

*targeting different numbers of variants*

As can be seen, at a DNA input of 20,000 copies the analytical sensitivity of RaDaR is predicted to be approximately 5-fold improved when targeting 6 vs 48 variants.

Of note, 2 of the 8 (25%) landmark positive LEMA patients, described in the manuscript, had ctDNA detected at levels below 0.0073% eVAF and thus would likely have been missed had a TSO500 like panel been used. This would have reduced the landmark clinical sensitivity, specificity, PPV and NPV from 30.4%, 98.3%, 87.5% and 78.4% to 21.7%, 98.3%, 83.3% and 76.3% respectively. Similarly, when considering all longitudinal samples (landmark or later), 17 of 63 (27%) ctDNA positive timepoints were detected at eVAF <0.0073% and thus might have been missed.

The above is an oversimplification as the LoD of a ‘TSO500 like’ panel being used in a truly agnostic fashion (i.e. no up-front tissue sequencing), would be inferior than that suggested by the described *in-silico* analysis. This is because the underlying, statistically driven, algorithm that calls a sample as positive or negative will differ between the two types of assay. In tumor informed approaches, knowing what you are looking for allows more confidence in low-level (VAF) calls and thus the calling threshold, and analytical sensitivity can be improved accordingly. Conversely, in tumor agnostic approaches, you don’t necessarily know what you are looking for and, as such, confidence in the observation of a low-level variants is reduced.

Finally, it is noteworthy that many tumor agnostic approaches currently/soon to be in use detect alternative targets (e.g. epigenetic changes), further complicating the comparison of agnostic vs informed methods.

#

# References

1. Flach S, Howarth K, Hackinger S, et al. Liquid BIOpsy for MiNimal RESidual DiSease Detection in Head and Neck Squamous Cell Carcinoma (LIONESS)—a personalised circulating tumour DNA analysis in head and neck squamous cell carcinoma. Br J Cancer [Internet] 2022;126(8):1186–95. Available from: https://doi.org/10.1038/s41416-022-01716-7
