# Supplementary material for: Recurrence prediction using circulating tumor DNA in patients with early-stage non-small cell lung cancer after treatment with curative intent: A retrospective validation study
Source: PLoS Med. 2025 Apr 15;22(4):e1004574. doi: 10.1371/journal.pmed.1004574 (PMC12021277; doi:10.1371/journal.pmed.1004574)

Supplementary figures appendix – Longitudinal plots

Longitudinal monitoring of ctDNA in plasma
True positives (1 of 3)
i.e. patients that did recur
and had ctDNA signal ≥14 days post treatment

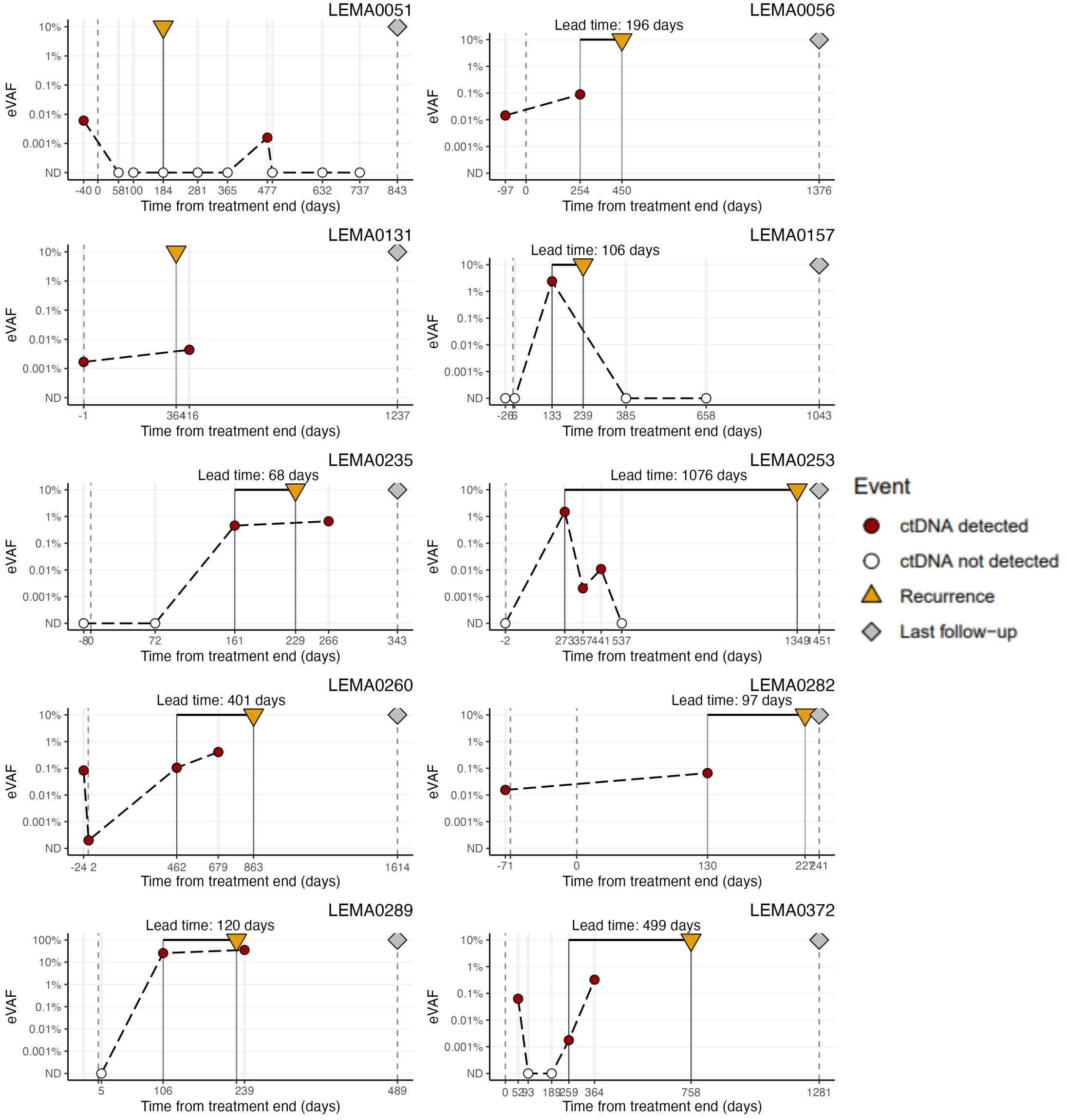

Longitudinal monitoring of ctDNA in plasma
True positives (2 of 3)

i.e. patients that did recur
and had ctDNA signal ≥14 days post treatment

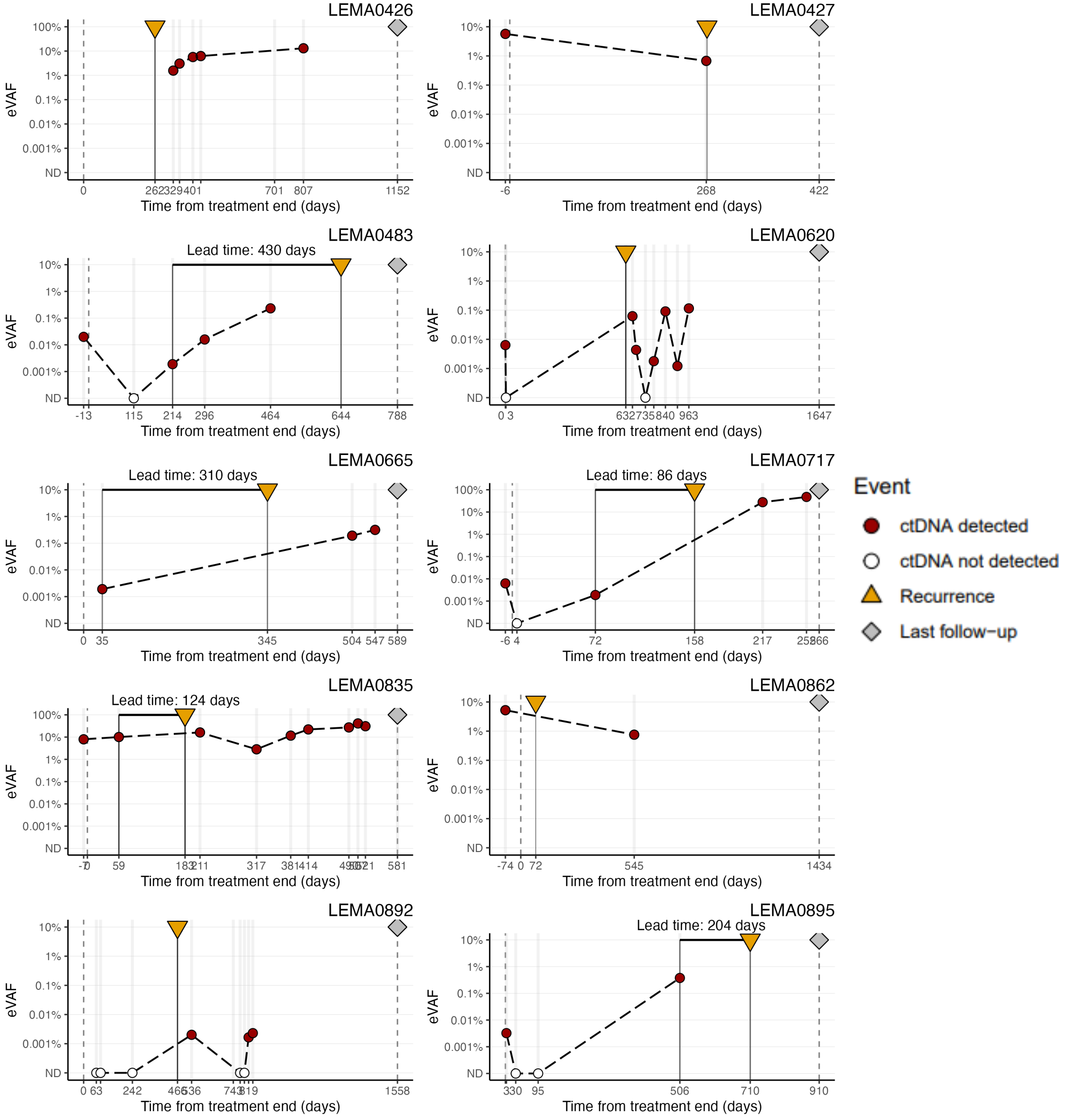

Longitudinal monitoring of ctDNA in plasma
True positives (3 of 3)
i.e. patients that did recur
and had ctDNA signal ≥14 days post
treatment

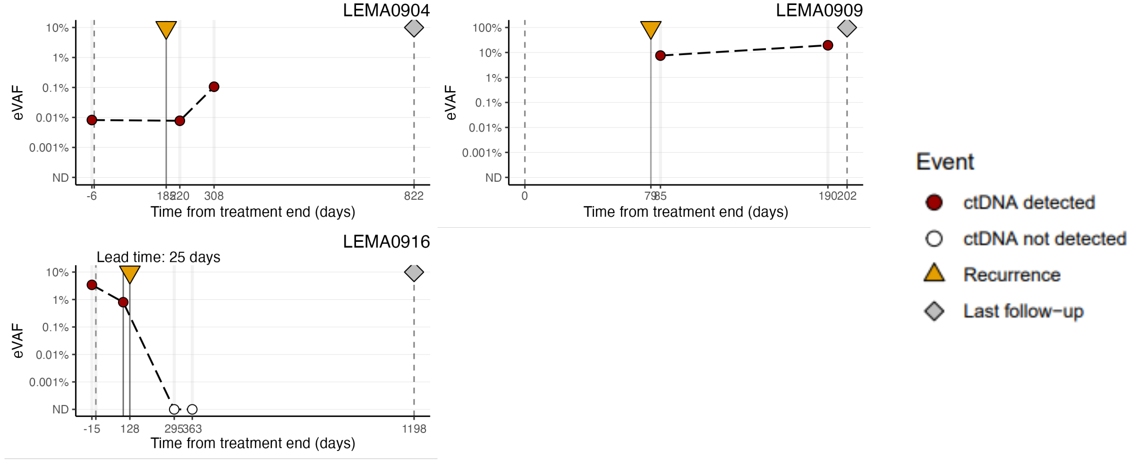

Longitudinal monitoring of ctDNA in plasma
Potential false negatives (1 of 2)

i.e. patients that recurred but no ctDNA signal ≥14 days post treatment

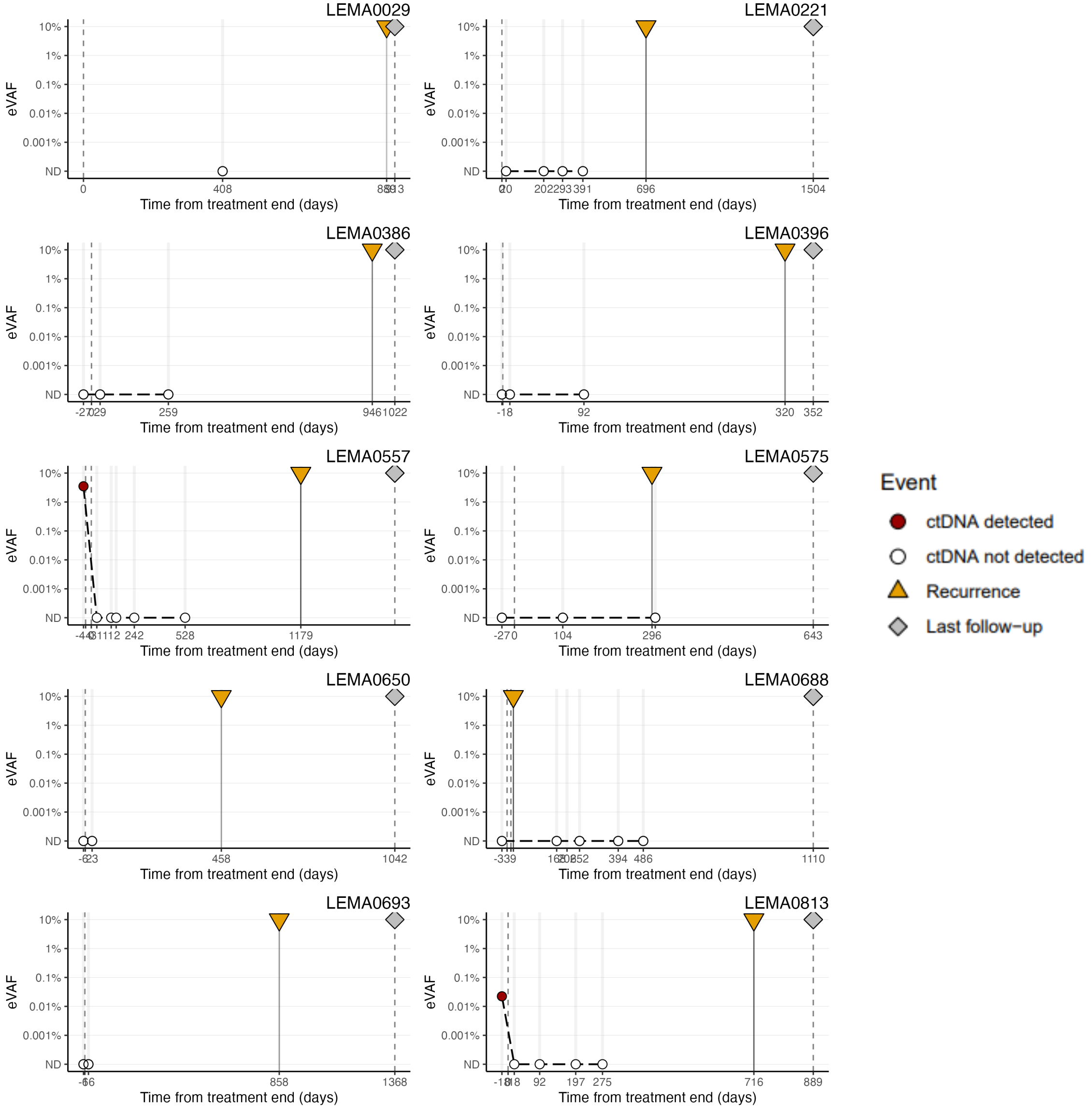

Longitudinal monitoring of ctDNA in plasma
Potential false negatives (2 of 2)

i.e. patients that recurred but no ctDNA signal ≥14 days post treatment

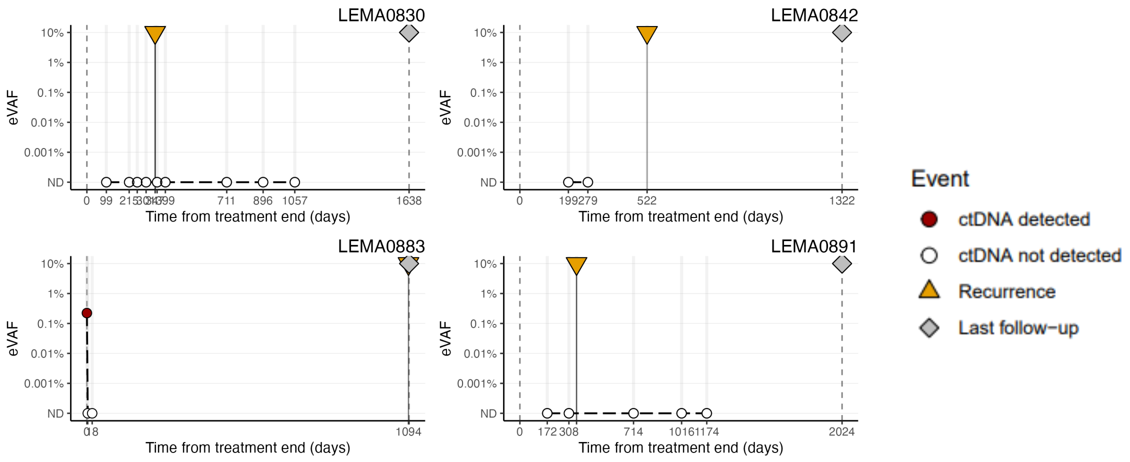

Longitudinal monitoring of ctDNA in plasma
Potential false positives
i.e. patients that did not recur but had ctDNA signal ≥14 days post treatment
IO, Immunotherapy
CTx, Chemotherapy
RTx, Radiotherapy

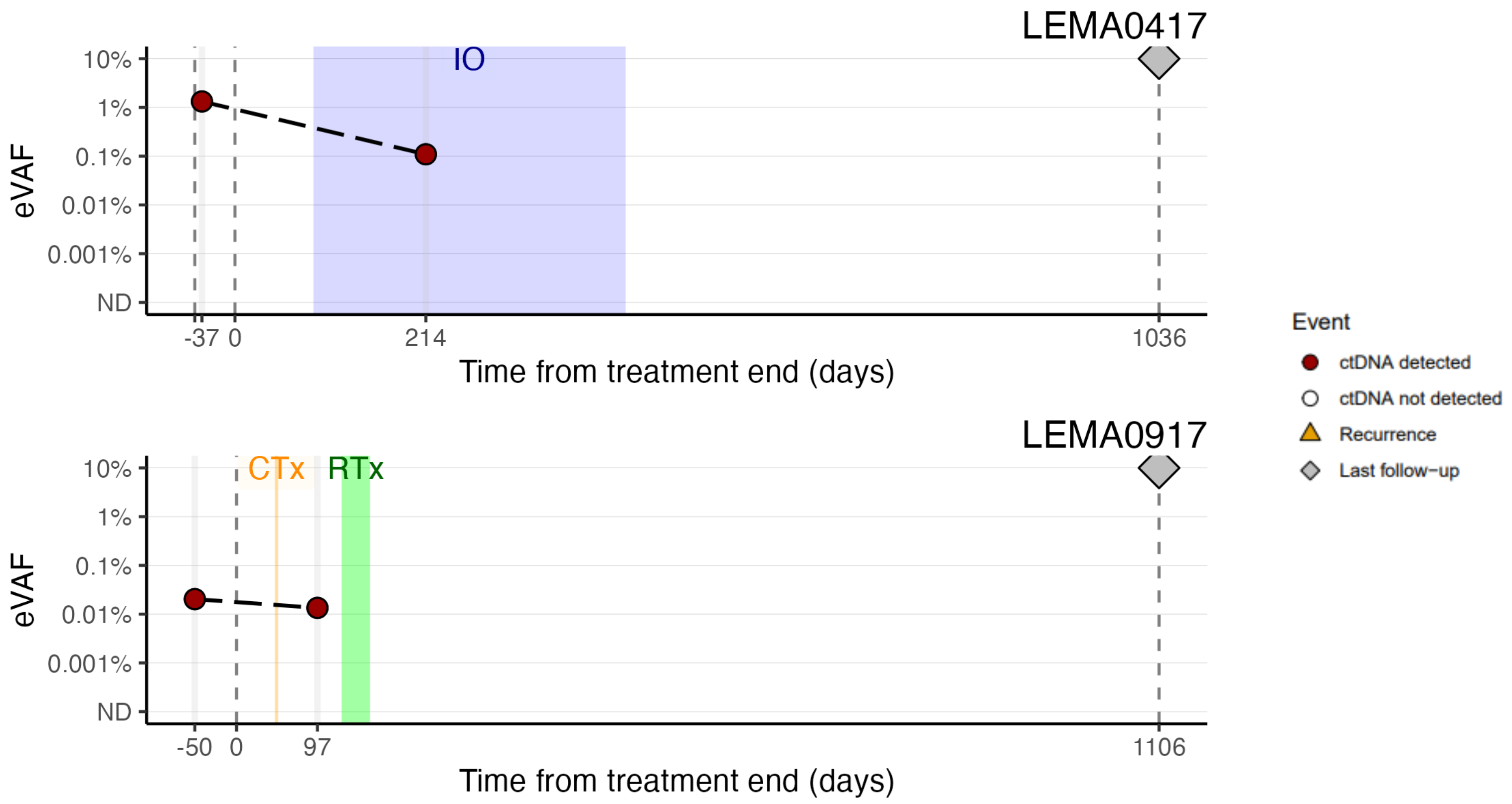

Longitudinal monitoring of ctDNA in plasma
True negatives (1 of 8)
i.e. patients that did not recur and had no ctDNA signal ≥14 days post treatment

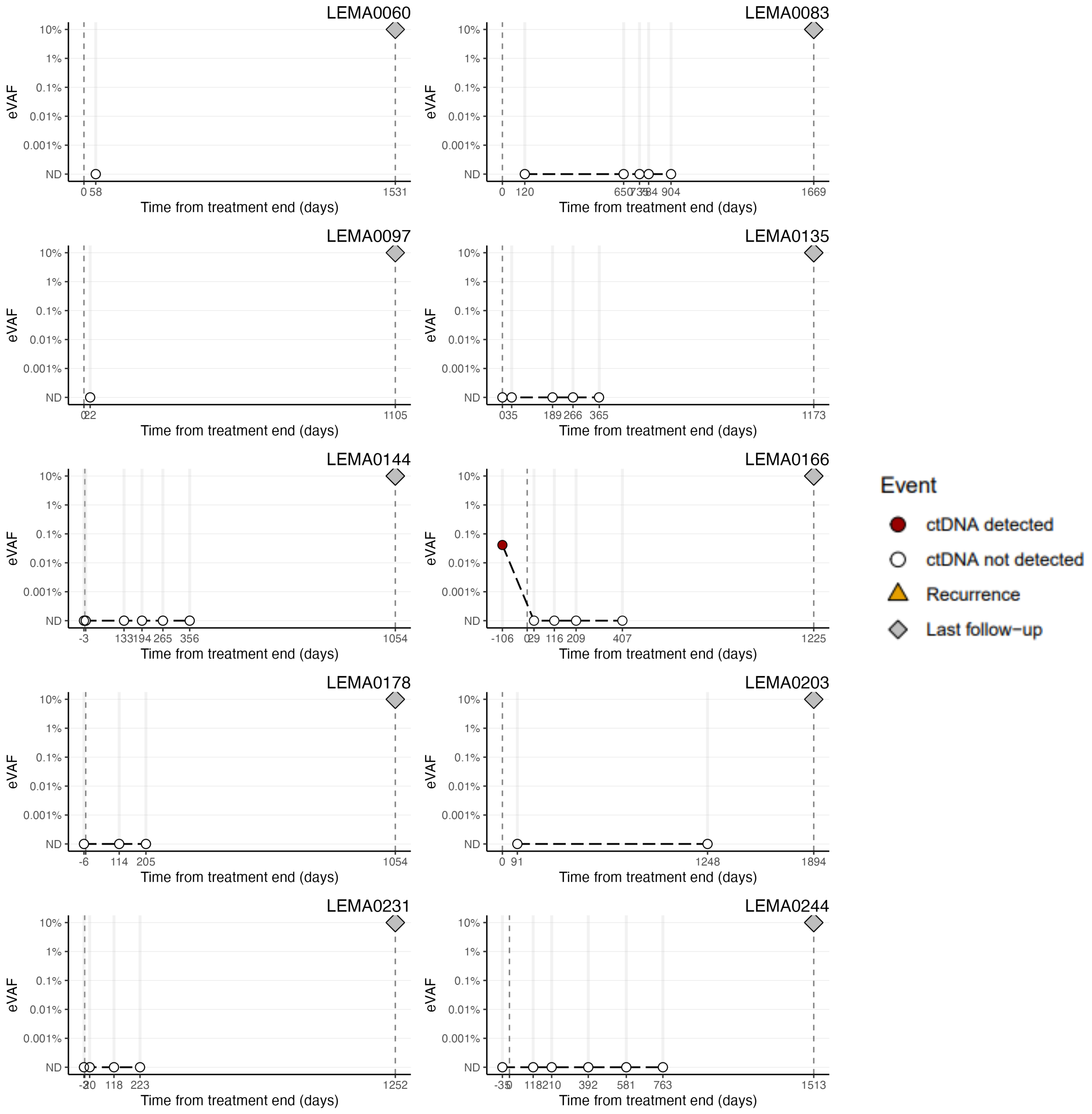

Longitudinal monitoring of ctDNA in plasma
True negatives (2 of 8)
i.e. patients that did not recur and had no ctDNA signal ≥14 days post treatment

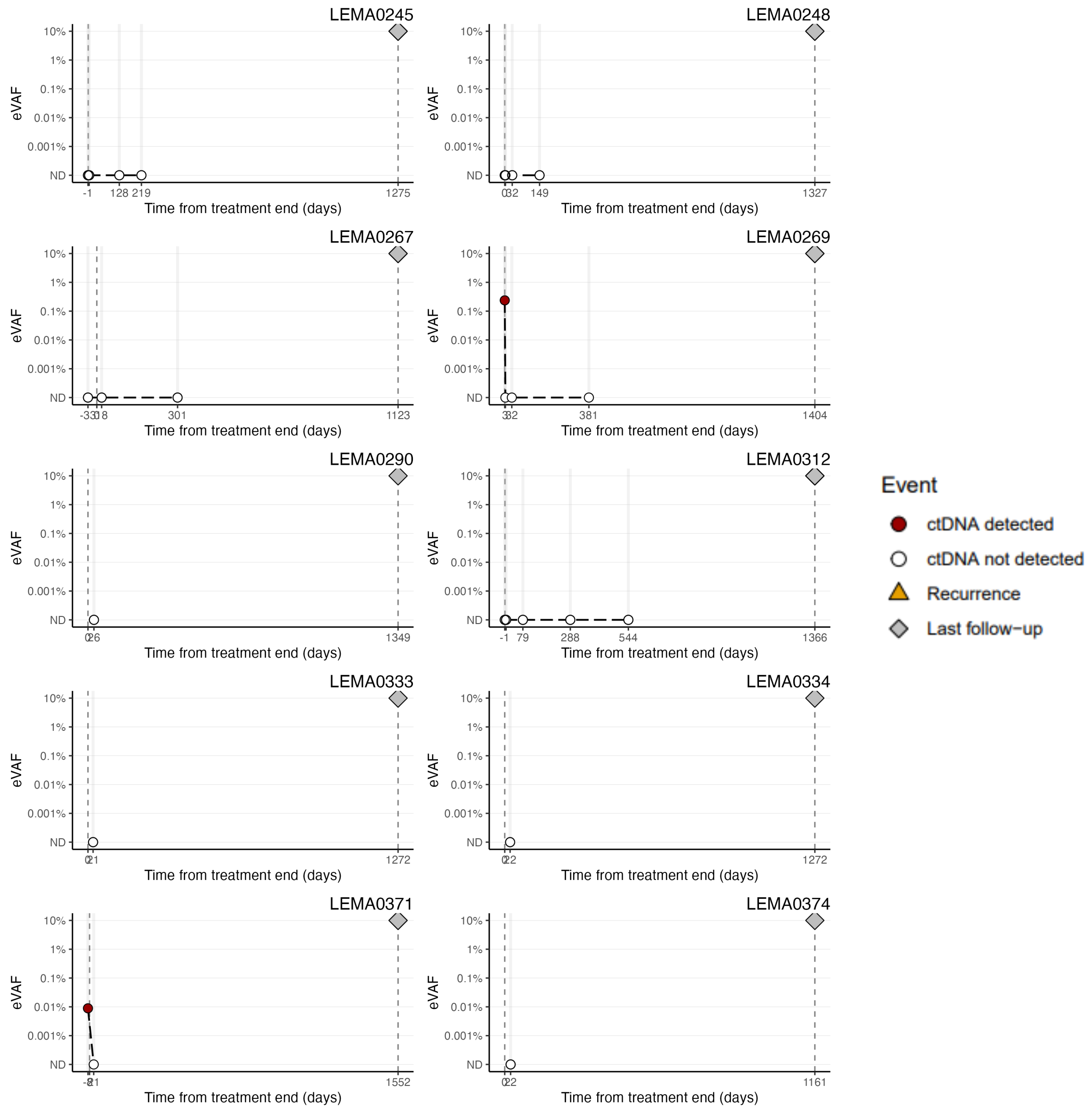

Longitudinal monitoring of ctDNA in plasma
True negatives (3 of 8)
i.e. patients that did not recur and had no ctDNA signal ≥14 days post treatment

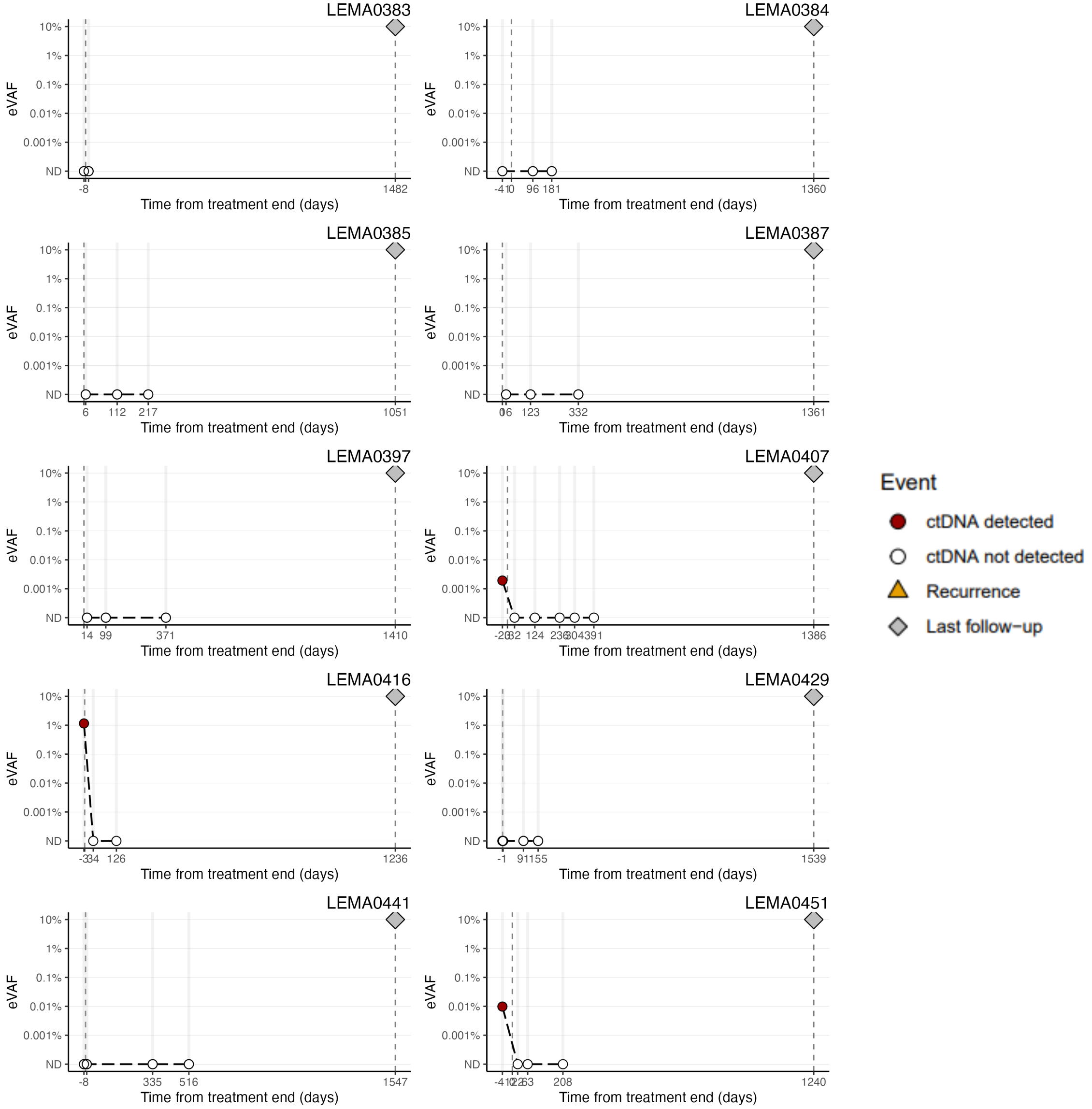

Longitudinal monitoring of ctDNA in plasma
True negatives (4 of 8)
i.e. patients that did not recur and had no ctDNA signal ≥14 days post treatment

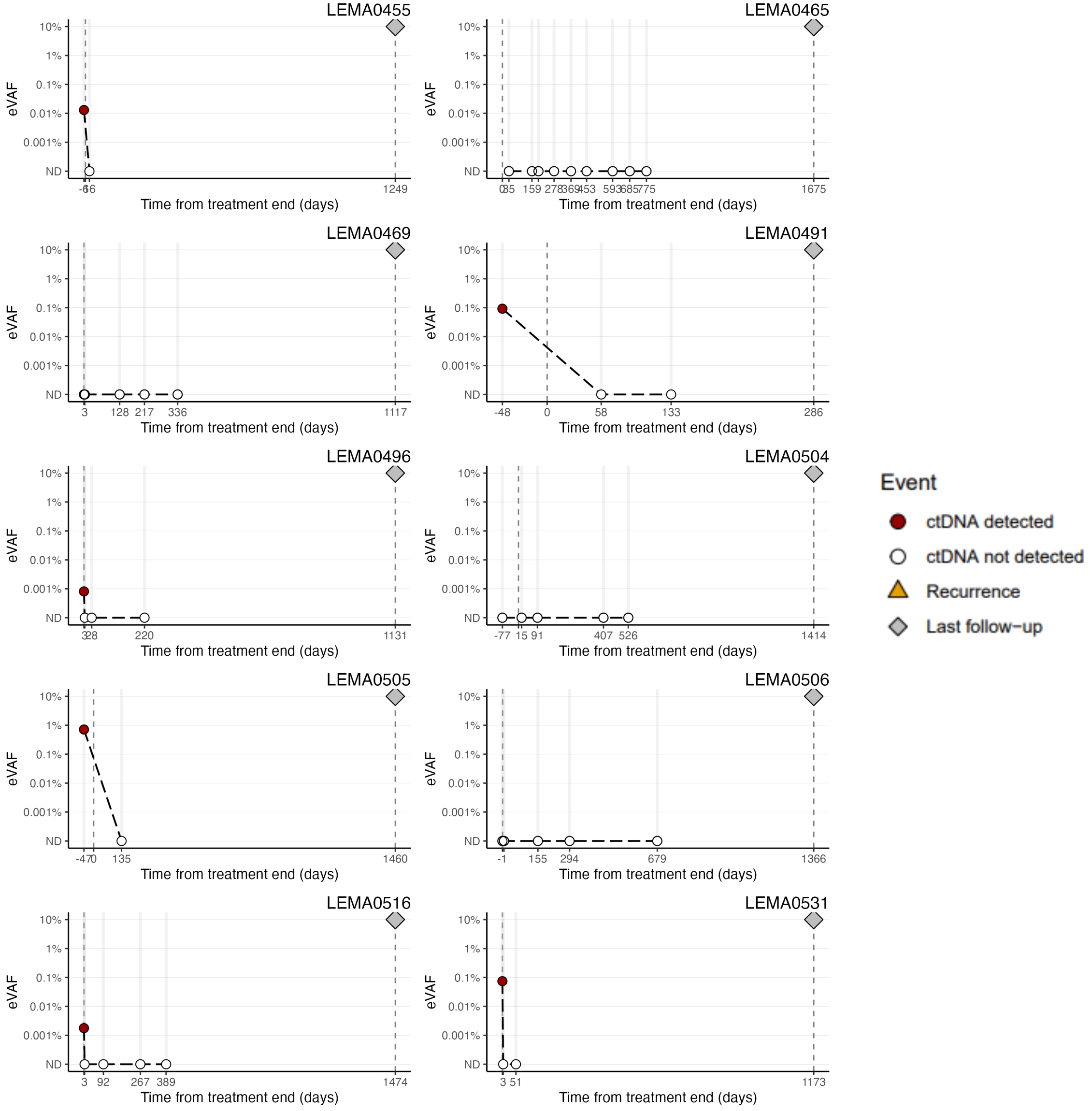

Longitudinal monitoring of ctDNA in plasma
True negatives (5 of 8)
i.e. patients that did not recur and had no ctDNA signal ≥14 days post treatment

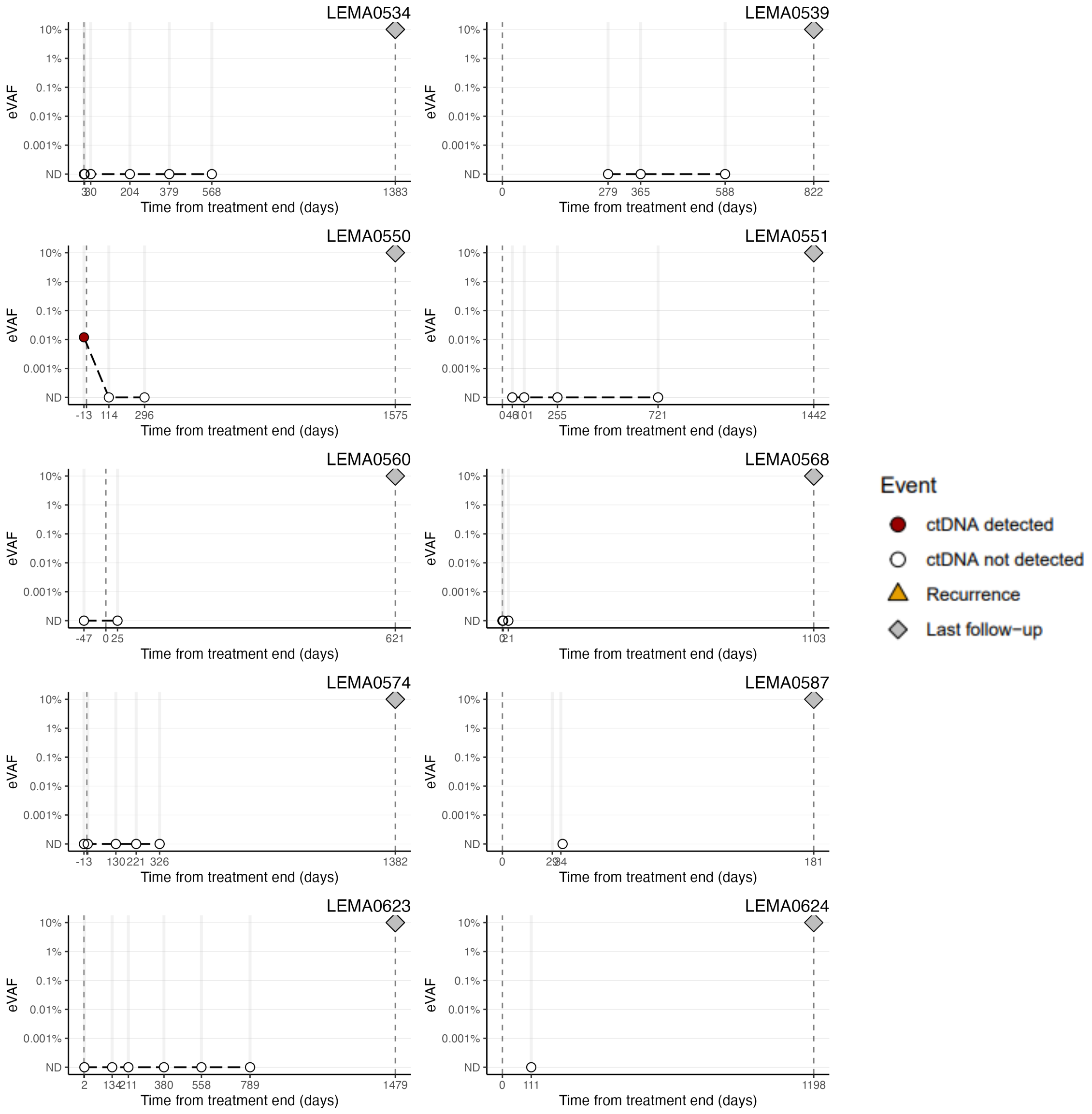

Longitudinal monitoring of ctDNA in plasma
True negatives (6 of 8)
i.e. patients that did not recur and had no ctDNA signal ≥14 days post treatment

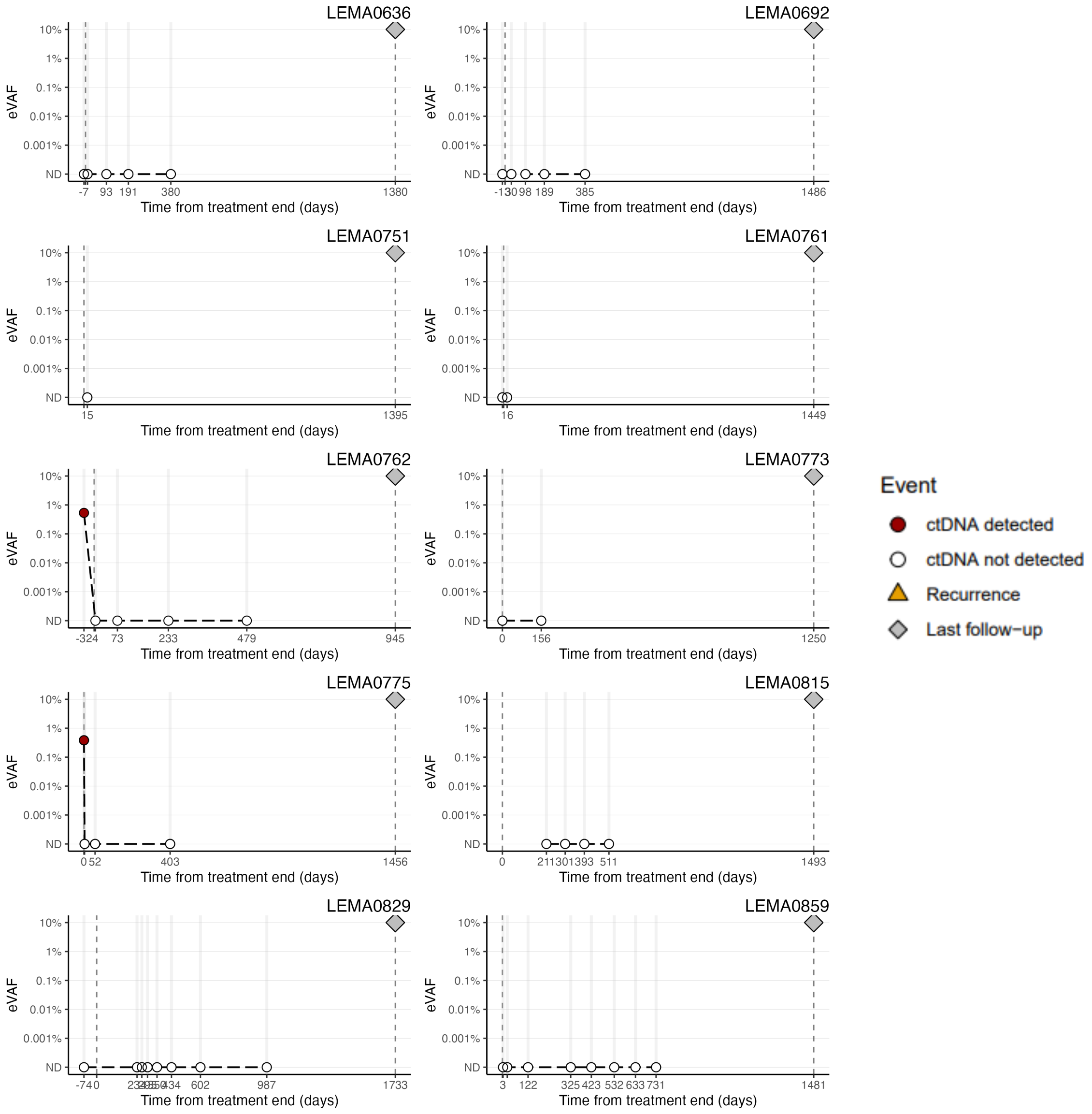

Longitudinal monitoring of ctDNA in plasma
True negatives (7 of 8)
i.e. patients that did not recur and had no ctDNA signal ≥14 days post treatment

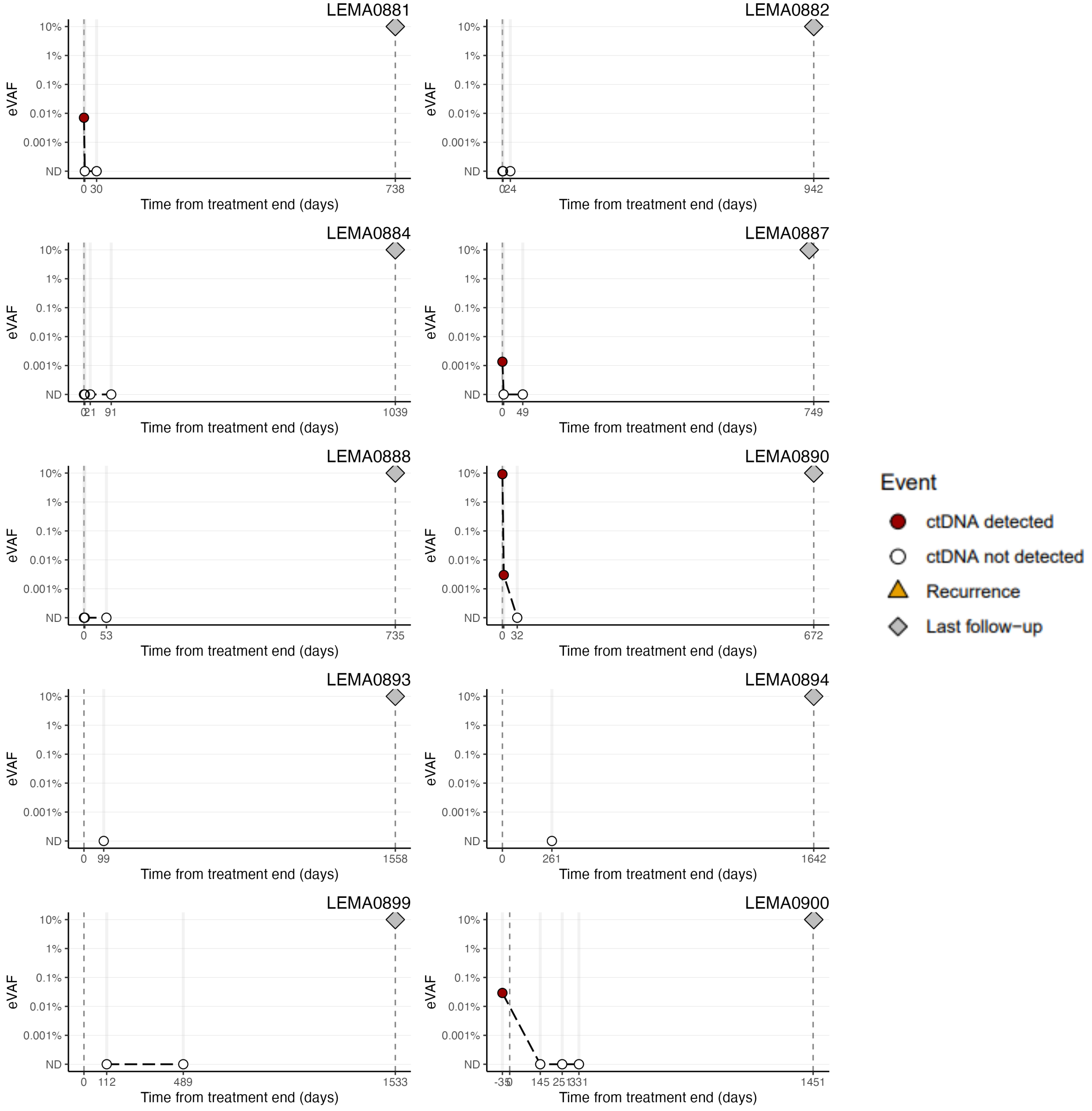

Longitudinal monitoring of ctDNA in plasma
True negatives (8 of 8)
i.e. patients that did not recur and had no ctDNA signal ≥14 days post treatment

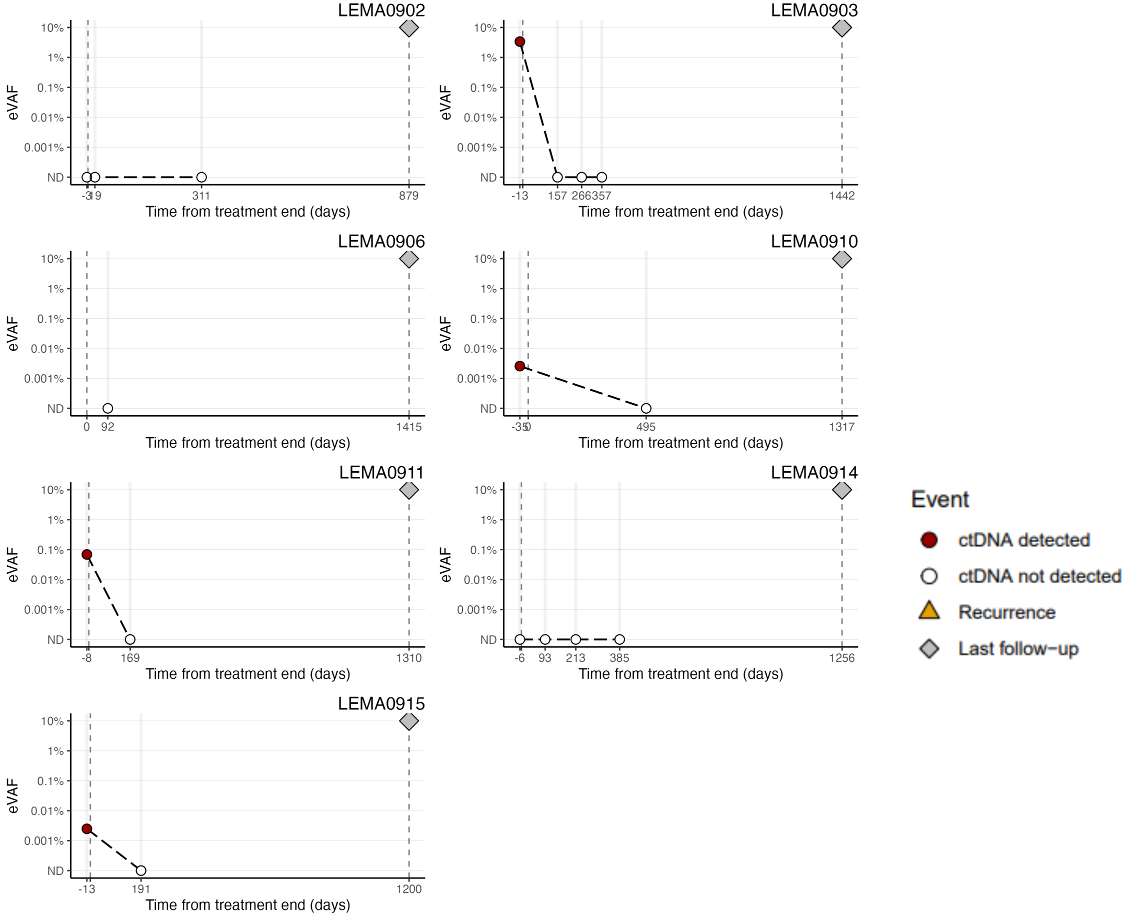

Supplement: S1 Appendix — Figures are grouped according to detection status during the observation timeframe (≥days post-treatment), and how that related to recurrence status, i.e., true positives, false negatives, false positives, and true negatives. (PDF) [file pmed.1004574.s028.pdf]
