# Supplementary material for: Recurrence prediction using circulating tumor DNA in patients with early-stage non-small cell lung cancer after treatment with curative intent: A retrospective validation study
Source: PLoS Med. 2025 Apr 15;22(4):e1004574. doi: 10.1371/journal.pmed.1004574 (PMC12021277; doi:10.1371/journal.pmed.1004574)
Supplement: S1 Text — (DOCX) [file pmed.1004574.s029.docx]

**S1 Text**

**Full list of collaborators**

Milou M.F. Schuurbiers, M.D.^1Φ^, Christopher G. Smith, Ph.D.^2Φ^, Koen J. Hartemink, M.D.^3^, Robert C. Rintoul, M.B..B.Ch., Ph.D.^4,5,6^, Davina Gale, Ph.D.^6,7∇^, Kim Monkhorst, Ph.D.^8^, Bas L.R. Mandos^1^, Anna L. Paterson, M.D., Ph.D.^9^, Daan van den Broek, M.D., Ph.D.^10^, Nitzan Rosenfeld, Ph.D.^6,7,11*^, Michel M. van den Heuvel, M.D., Ph.D.^1*^ on behalf of the LEMA Study Group^^^ and the LUCID Study Group^^^

^Φ^These authors contributed equally to this work

^*^These authors jointly supervised this work

^∇^Current address: AstraZeneca, Cambridge, United Kingdom

^^^

**LEMA Study Group:**

Robert Schouten^12^, Sjaak Burgers^12^, Joop van den Brand^13^, Germaine Liebrechts-Akkerman^14^, Anne van Lindert^15^, Stefan Willems^16^

**LUCID Study Group**

Katrin Heider^6,7∇^, Andrea Ruiz-Valdepenas^6,7^, Viona Rundell^17^, Jerome Wulff^17^, Jenny Castedo^6,5^, Susan Harden^18^, Helena Rayment^17^, David Gilligan^5,18^, Doris Rassl^6,5^

Affiliations

1. Department of Pulmonary Diseases, Radboud University Medical Center, Nijmegen, Netherlands
2. NeoGenomics, Babraham Research Park, Cambridge, United Kingdom
3. Department of Surgery, The Netherlands Cancer Institute, Amsterdam, Netherlands
4. Department of Oncology, University of Cambridge, Cambridge, United Kingdom
5. Royal Papworth Hospital NHS Foundation Trust, Cambridge, United Kingdom
6. Cancer Research UK Cambridge Centre, Cambridge, United Kingdom
7. Cancer Research UK Cambridge Institute, University of Cambridge, Cambridge, United Kingdom
8. Department of Pathology, The Netherlands Cancer Institute, Amsterdam, Netherlands
9. Department of Histopathology, Cambridge University Hospitals, Cambridge, United Kingdom
10. Department of Laboratory Medicine, The Netherlands Cancer Institute, Amsterdam, Netherlands
11. Barts Cancer Institute, Queen Mary University of London, London, United Kingdom
12. Department of Thoracic Oncology, The Netherlands Cancer Institute, Amsterdam, Netherlands
13. Department of Pulmonary Diseases, Meander Medical Center, Amersfoort, Netherlands
14. Department of Pathology, Meander Medical Center, Amersfoort, Netherlands
15. Department of Pulmonary Diseases, University Medical Center Utrecht, Utrecht, Netherlands
16. Department of Pathology, University Medical Center Utrecht, Utrecht, Netherlands
17. Cambridge Clinical Trials Unit – Cancer Theme, Cambridge, United Kingdom
18. Addenbrooke’s Hospital, Cambridge, United Kingdom
