# Supplementary material for: Recurrence prediction using circulating tumor DNA in patients with early-stage non-small cell lung cancer after treatment with curative intent: A retrospective validation study
Source: PLoS Med. 2025 Apr 15;22(4):e1004574. doi: 10.1371/journal.pmed.1004574 (PMC12021277; doi:10.1371/journal.pmed.1004574)
Supplement: S14 Table — Summary of studies of post-treatment ctDNA in patients with early-stage NSCLC, and its predictive ability for recurrence detection. Studies are split by assay type; either tumor-informed or tumor-agnostic. Sensitivity, specificity, positive predictive value (PPV), and negative predictive value (NPV) are provided for each study (patient number shown in parenthesize), as well as median lead time from initial ctDNA detection to recurrence. Some values are inferred or omitted where data is unavailable or unclear. Some data are based on conference proceedings, without peer-review, and so should be interpreted with caution. This list is not exhaustive, but highlights the wide array of studies and assays that have explored ctDNA in this setting, and the diversity in predictive performance (though many values are based on relatively small numbers). References are provided at the end of this Supporting information document. PPV, Positive Predictive Value; NPV, Negative Predictive Value. (DOCX) [file pmed.1004574.s014.docx]

**S14 Table** Summary of select studies of ctDNA utility for longitudinal (post treatment) MRD detection.

| **Assay type** | **Study** | **Total patients in study** | **Stages** | **Assay** | **Sensitivity** | **Specificity** | **PPV** | **NPV** | **median lead time (months)** | **Comments** |
| --- | --- | --- | --- | --- | --- | --- | --- | --- | --- | --- |
| Tumor informed | Abbosh et al, 2017(1) | 25 | I-III | Signatera | 92.9 (13/14) | 90.9 (10/11) | 92.9 (13/14) | 90.9 (10/11) | 2.3 |  |
|  | Abbosh et al, 2020(2) | 78 | I-III | AMP | 82.2 (37/45) | 95.7 (22/23) | 97.4 (37/38) | 73.3 (22/30) | 5 | Based on conference proceedings |
|  | Abbosh et al, 2023(3) | 70 | I-III | AMP | 84.3 (59/70) | 95.1 (58/61) | 95.2 (59/62) | 84.1 (58/69) | 3.9 | 2/42 (95.2% specificity) patients with no recurrence were ctDNA+. 1/19 (94.7% specificity) patients with second primary were ctDNA+ |
|  | Black et al, 2023(4) | 178 | I-III | NeXT Personal | 94 | 89 | 85 | 96 | 7.4 | Based on conference proceedings. Patient numbers unknown |
|  | Chaudhuri et al, 2017(5) | 37 | I-III | CAPP-Seq | 100 (20/20) | 100 (17/17) | 100 (20/20) | 100 (17/17) | 5.2 | Only patients with ctDNA detected pretreatment. 64% patients had stage III disease. 18% had stage I |
|  | Chen et al, 2023(6) | 151 | IA-III | PROPHET (in house) | 84.2 (16/19) | 92.3 (84/91) | 69.6 (16/23) | 96.6 (84/87) | 9.8 | 64.5% of pts were stage III or higher |
|  | Gale et al, 2021 (LUCID)(7) | 88 | I-III | RaDaR | 64.3 (18/28) | 95.9 (47/49) | 90.0 (18/20) | 82.5 (47/57) | 7 |  |
|  | Isbell et al, 2024(8) | 46 | I-III | Foresight CLARITY (PhasED-Seq) | 66.7 (12/18) | 100 (27/27) | 100 (12/12) | 81.8 (27/33) | - | Based on conference proceedings. **Landmark analysis** |
|  | John et al, 2024 (ADAURA)(9) | 220 | IB-IIIA | RaDaR | 64.6 (62/96) | 95.2 (118/124) | 91.2 (62/68) | 77.6 (118/152) | 4.7 | Half of cohort treated with adjuvant Osimertinib (as ADAURA study), half placebo |
|  | Lebow et al, 2023(10) | 17 | I-III | Signatera | 100 (9/9) | 100 (8/8) | 100 (9/9) | 100 (8/8) | 5.4 |  |
|  | LEMA (current manuscript) | 130 | I-iII | RaDaR | 60.5 (23/38) | 97.4 (76/78) | 92.0 (23/25) | 83.5 (76/91) | 6.8 |  |
|  | LEMA and LUCID combined (current manuscript) | 218 | I-III | RaDaR | 62.1 (41/66) | 96.9 (123/127) | 91.1 (41/45) | 83.1 (123/148) | 6.8 |  |
|  | Martin et al, 2024(11) | 108 | I-II | Signatera | 80 (8/10) | 95.9 (94/98) | 66.7 (8/12) | 97.9 (94/96) | 5.5 | Samples collected prospectively but analysis done retrospectively |
|  | Moding et al, 2020(12) | 65 | IIB-IIIB | CAPP-Seq, chemoradiation only patients | 100 (6/6) | 100 (6/6) | 100 (6/6) | 100 (6/6) | - | Excludes patients with no tissue or pre-treatment plasma result. Here excluding patients previously reported in Chaudhuri et al, 2017 |
|  |  |  |  | CAPP-Seq, CICI patients, pre-CICI timepoint | 100 (8/8) | 73.3 (11/15) | 66.7 (8/12) | 100 (11/11) | 4.1 | Excludes patients with no tissue or pre-treatment plasma result |
|  | Tan et al, 2021(13) | 57 | IA-IIIB | Signatera | 46.7 (7/15) | 100 (42/42)) | 100 (7/7) | 84.0 (42/50) | 2.8 |  |
|  | Widman et al, 2024(14) | 14 | IA-IIIA | MRD-EDGE | 100 (5/5) | 66.7 (6/9) | 62.5 (5/8) | 100 (6/6) | - | Several samples drawn during adjuvant therapy |
| Tumor agnostic | Li et al, 2021(15) | 123 | I-IIIA | 425 cancer gene panel | 60.7 (17/28) | 78.0 (71/91) | 45.9 (17/37) | 86.6 (71/82) | 8.7 |  |
|  | Peng et al, 2020(16) | 77 | I-III | cSMART | 54.3 (19/35) | 73.8 (31/42) | 63.3 (19/30) | 66.0 (31/47) | 12.6 |  |
|  | Qiu et al, 2021(17) | 91 | I-III | ATG-seq | 79.4 (27/34) | 86.0 (49/57) | 77.1 (27/35) | 87.5 (49/56) | 2.9 |  |
|  | Wang et al, 2022(18) | 128 | I-III | GeneseeqPrimeTM (425 cancer gene panel) | 73.5 (25/34) | 83.1 (69/83) | 64.1 (25/39) | 88.5 (69/78) | 4.8 |  |
|  | Xia et al, 2022(19) | 329 | I-III | Genecast MRD - lung | 30 (21/70) | 98.1 (254/259) | 80.8 (21/26) | 83.8 (254/303) | - |  |
|  | Zhang et al, 2022(20) | 216 | I-III | In-house (338 gene panel) | 87.2 (41/47) | 97.4 (184/189) | 89.1 (41/46) | 96.8 (184/190) | 3.4 | Fixed panel but used a combination of tumor-informed and tumor-naïve calling |

Summary of studies of post-treatment ctDNA in patients with early-stage NSCLC, and its predictive ability for recurrence detection. Studies are split by assay type; either tumor-informed or tumor-agnostic. Sensitivity, specificity, positive predictive value (PPV) and negative predictive value (NPV) are provided for each study (patient number shown in parenthesise), as well as median lead time from initial ctDNA detection to recurrence. Some values are inferred or omitted where data is unavailable or unclear. Some data are based on conference proceedings, without peer-review, and so should be interpreted with caution.

This list is not exhaustive, but highlights the wide array of studies and assays that have explored ctDNA in this setting, and the diversity in predictive performance (though many values are based on relatively small numbers).

*PPV = Positive Predictive Value, NPV = Negative Predictive Value.*

References

1.	Abbosh C, Birkbak NJ, Wilson GA, Jamal-Hanjani M, Constantin T, Salari R, et al. Phylogenetic ctDNA analysis depicts early-stage lung cancer evolution. Nature [Internet]. 2017;545(7655):446–51. Available from: https://doi.org/10.1038/nature22364

2.	Abbosh C, Frankell A, Garnett A, Harrison T, Weichert M, Licon A, et al. Abstract CT023: Phylogenetic tracking and minimal residual disease detection using ctDNA in early-stage NSCLC: A lung TRACERx study. Cancer Res [Internet]. 2020 Aug 15;80(16_Supplement):CT023–CT023. Available from: https://doi.org/10.1158/1538-7445.AM2020-CT023

3.	Abbosh C, Frankell AM, Harrison T, Kisistok J, Garnett A, Johnson L, et al. Tracking early lung cancer metastatic dissemination in TRACERx using ctDNA. Nature [Internet]. 2023;616(7957):553–62. Available from: https://doi.org/10.1038/s41586-023-05776-4

4.	Black JRM, Frankell AM, Veeriah S, Colopi M, Hill M, Abbott CW, et al. LBA55 An ultra-sensitive and specific ctDNA assay provides novel pre-operative disease stratification in early stage lung cancer. Annals of Oncology [Internet]. 2023 Oct 1;34:S1294. Available from: https://doi.org/10.1016/j.annonc.2023.10.049

5.	Chaudhuri AA, Chabon JJ, Lovejoy AF, Newman AM, Stehr H, Azad TD, et al. Early Detection of Molecular Residual Disease in Localized Lung Cancer by Circulating Tumor DNA Profiling. Cancer Discov [Internet]. 2017 Dec 4;7(12):1394–403. Available from: https://doi.org/10.1158/2159-8290.CD-17-0716

6.	Chen K, Yang F, Shen H, Wang C, Li X, Chervova O, et al. Individualized tumor-informed circulating tumor DNA analysis for postoperative monitoring of non-small cell lung cancer. Cancer Cell [Internet]. 2023; Available from: https://www.sciencedirect.com/science/article/pii/S1535610823002866

7.	Gale D, Heider K, Ruiz-Valdepenas A, Hackinger S, Perry M, Marsico G, et al. Residual ctDNA after treatment predicts early relapse in patients with early-stage non-small cell lung cancer. Annals of Oncology [Internet]. 2022 May 1;33(5):500–10. Available from: https://doi.org/10.1016/j.annonc.2022.02.007

8.	Isbell JM, Goldstein JS, Hamilton EG, Liu SY, Eichholz J, Buonocore DJ, et al. Ultrasensitive circulating tumor DNA (ctDNA) minimal residual disease (MRD) detection in early stage non-small cell lung cancer (NSCLC). Journal of Clinical Oncology [Internet]. 2024 May 29;42(16_suppl):8078. Available from: https://doi.org/10.1200/JCO.2024.42.16_suppl.8078

9.	John T, Grohe C, Goldman JW, Kato T, Laktionov KK, Bonanno L, et al. Molecular residual disease (MRD) analysis from the ADAURA trial of adjuvant (adj) osimertinib in patients (pts) with resected EGFR-mutated (EGFRm) stage IB–IIIA non-small cell lung cancer (NSCLC). Journal of Clinical Oncology [Internet]. 2024 May 29;42(16_suppl):8005. Available from: https://doi.org/10.1200/JCO.2024.42.16_suppl.8005

10.	Lebow ES, Shaverdian N, Eichholz JE, Kratochvil LB, McCune M, Murciano-Goroff YR, et al. ctDNA-based detection of molecular residual disease in stage I-III non-small cell lung cancer patients treated with definitive radiotherapy. Front Oncol [Internet]. 2023;13. Available from: https://www.frontiersin.org/articles/10.3389/fonc.2023.1253629

11.	Martin TK, Dinerman A, Sudhaman S, Budde G, Palsuledesai CC, Krainock M, et al. Early real-world experience monitoring circulating tumor DNA in resected early-stage non&#x2013;small cell lung cancer. J Thorac Cardiovasc Surg [Internet]. 2024 Jul 19; Available from: https://doi.org/10.1016/j.jtcvs.2024.01.017

12.	Moding EJ, Liu Y, Nabet BY, Chabon JJ, Chaudhuri AA, Hui AB, et al. Circulating tumor DNA dynamics predict benefit from consolidation immunotherapy in locally advanced non-small-cell lung cancer. Nat Cancer [Internet]. 2020;1(2):176–83. Available from: https://doi.org/10.1038/s43018-019-0011-0

13.	Tan AC, Lai GGY, Saw SPL, Chua KLM, Takano A, Ong BH, et al. Detection of circulating tumor DNA with ultradeep sequencing of plasma cell-free DNA for monitoring minimal residual disease and early detection of recurrence in early-stage lung cancer. Cancer [Internet]. 2024 May 15;130(10):1758–65. Available from: https://doi.org/10.1002/cncr.35263

14.	Widman AJ, Shah M, Frydendahl A, Halmos D, Khamnei CC, Øgaard N, et al. Ultrasensitive plasma-based monitoring of tumor burden using machine-learning-guided signal enrichment. Nat Med [Internet]. 2024;30(6):1655–66. Available from: https://doi.org/10.1038/s41591-024-03040-4

15.	Li N, Wang BX, Li J, Shao Y, Li MT, Li JJ, et al. Perioperative circulating tumor DNA as a potential prognostic marker for operable stage I to IIIA non–small cell lung cancer. Cancer [Internet]. 2022 Feb 15;128(4):708–18. Available from: https://doi.org/10.1002/cncr.33985

16.	Peng M, Huang Q, Yin W, Tan S, Chen C, Liu W, et al. Circulating Tumor DNA as a Prognostic Biomarker in Localized Non-small Cell Lung Cancer. Front Oncol [Internet]. 2020;10. Available from: https://www.frontiersin.org/articles/10.3389/fonc.2020.561598

17.	Qiu B, Guo W, Zhang F, Lv F, Ji Y, Peng Y, et al. Dynamic recurrence risk and adjuvant chemotherapy benefit prediction by ctDNA in resected NSCLC. Nat Commun [Internet]. 2021;12(1):6770. Available from: https://doi.org/10.1038/s41467-021-27022-z

18.	Wang S, Li M, Zhang J, Xing P, Wu M, Meng F, et al. Circulating tumor DNA integrating tissue clonality detects minimal residual disease in resectable non-small-cell lung cancer. J Hematol Oncol [Internet]. 2022;15(1):137. Available from: https://doi.org/10.1186/s13045-022-01355-8

19.	Xia L, Mei J, Kang R, Deng S, Chen Y, Yang Y, et al. Perioperative ctDNA-Based Molecular Residual Disease Detection for Non–Small Cell Lung Cancer: A Prospective Multicenter Cohort Study (LUNGCA-1). Clinical Cancer Research [Internet]. 2022 Aug 2;28(15):3308–17. Available from: https://doi.org/10.1158/1078-0432.CCR-21-3044

20.	Zhang JT, Liu SY, Gao W, Liu SYM, Yan HH, Ji L, et al. Longitudinal Undetectable Molecular Residual Disease Defines Potentially Cured Population in Localized Non–Small Cell Lung Cancer. Cancer Discov [Internet]. 2022 Jul 6;12(7):1690–701. Available from: https://doi.org/10.1158/2159-8290.CD-21-1486
